# The SIDER2 elements, interspersed repeated sequences that populate the *Leishmania *genomes, constitute subfamilies showing chromosomal proximity relationship

**DOI:** 10.1186/1471-2164-9-263

**Published:** 2008-06-02

**Authors:** Jose M Requena, Cristina Folgueira, Manuel C López, M Carmen Thomas

**Affiliations:** 1 Centro de Biología Molecular "Severo Ochoa" (CSIC-UAM), Universidad Autónoma de Madrid, 28049 Madrid, Spain; 2 Departamento de Biología Molecular, Instituto de Parasitología y Biomedicina "López-Neyra", CSIC, 18000 Granada, Spain

## Abstract

**Background:**

Protozoan parasites of the genus *Leishmania *are causative agents of a diverse spectrum of human diseases collectively known as leishmaniasis. These eukaryotic pathogens that diverged early from the main eukaryotic lineage possess a number of unusual genomic, molecular and biochemical features. The completion of the genome projects for three *Leishmania *species has generated invaluable information enabling a direct analysis of genome structure and organization.

**Results:**

By using DNA macroarrays, made with *Leishmania infantum *genomic clones and hybridized with total DNA from the parasite, we identified a clone containing a repeated sequence. An analysis of the recently completed genome sequence of *L. infantum*, using this repeated sequence as bait, led to the identification of a new class of repeated elements that are interspersed along the different *L. infantum *chromosomes. These elements turned out to be homologues of SIDER2 sequences, which were recently identified in the *Leishmania major *genome; thus, we adopted this nomenclature for the *Leishmania *elements described herein. Since SIDER2 elements are very heterogeneous in sequence, their precise identification is rather laborious. We have characterized 54 LiSIDER2 elements in chromosome 32 and 27 ones in chromosome 20. The mean size for these elements is 550 bp and their sequence is G+C rich (mean value of 66.5%). On the basis of sequence similarity, these elements can be grouped in subfamilies that show a remarkable relationship of proximity, i.e. SIDER2s of a given subfamily locate close in a chromosomal region without intercalating elements. For comparative purposes, we have identified the SIDER2 elements existing in *L. major *and *Leishmania braziliensis *chromosomes 32. While SIDER2 elements are highly conserved both in number and location between *L. infantum *and *L. major*, no such conservation exists when comparing with SIDER2s in *L. braziliensis *chromosome 32.

**Conclusion:**

SIDER2 elements constitute a relevant piece in the *Leishmania *genome organization. Sequence characteristics, genomic distribution and evolutionarily conservation of SIDER2s are suggestive of relevant functions for these elements in *Leishmania*. Apart from a proved involvement in post-trancriptional mechanisms of gene regulation, SIDER2 elements could be involved in DNA amplification processes and, perhaps, in chromosome segregation as centromeric sequences.

## Background

Repetitive DNA sequences constitute a substantial proportion of eukaryotic genomes. For example, in mammals they account for nearly half of the genome, and in some plants they constitute up to 90% of the genome [1]. Most of these repeated DNAs are, or were originated from, transposable elements (TEs, also known mobile elements) through transposing and duplicating events. On the basis of mechanisms of their transposition, TEs can be divided into two classes: retrotransposons, which proliferate via reverse transcription, and DNA transposons, which move strictly through DNA intermediates. Frequently, genomes harbour few active TEs; instead, genomes contains multiple repetitive elements representing remnants (or dead elements) derived from TEs [2]. Although repetitive DNA elements have been often considered as "selfish" or "parasitic" DNAs, the now growing evidence is that these elements are involved in shaping genomes and are playing important role in epigenetic regulation of genome expression [1,3].

Protozoan parasites of the genus *Leishmania *are causative agents of a complex of diseases known as leishmaniasis. The burden associated with these diseases remains important: 1.5–2 million new cases per year and 350 million people at risk in 88 countries [4]. Apart from its impact in human health, *Leishmania *parasites and related trypanosomes (i.e. *Trypanosoma cruzi *and *Trypanosoma brucei*) are being extensively studied because of peculiar molecular and cellular characteristics. The genome of *Leishmania major *was sequenced [5], and more recently the genome sequences for two other *Leishmania *species (*Leishmania infantum *and *Leishmania braziliensis*) have been also deciphered [6]. The comparison of these sequences reveals marked conservation of the genome architecture within the *Leishmania *genus, showing similar gene content and a remarkable degree of synteny [7]. The organization of protein-coding genes into long, strand-specific, polycistronic clusters is a conspicuous feature of the *Leishmania *species, also observed in the *T. brucei *and *T. cruzi *genomes [8]. This peculiar gene organization seems to be related to the lack of transcriptional control by RNA polymerase II promoters; rather, transcription initiation appears to begin in a low fidelity manner transcribing long polycistronic precursor transcripts [9]. Despite having diverged 200 to 500 million years ago, the genomes of *L. major*, *T. brucei *and *T. cruzi *are highly synthenic. For example, 68 and 75% of the genes in *T. brucei *and *L. major *remain in the same gene order [8]. In spite of this conservation in chromosome organization, the genomes of these trypanosomes differ in the content of repeated sequences. Unlike *Leishmania*, the genomes of *T. brucei *and *T. cruzi *are riddled with interspersed elements [10-12].

The *Leishmania *genome is relatively poor in repeated sequences. The first repetitive DNA sequence characterized in *Leishmania *corresponded to the telomeric repeats [13]. Afterwards, multiple tandem repeats of a 60-bp sequence, named Lmet2, were found on at least six chromosomes of parasites of the *L. donovani *complex, being absent from other *Leishmania *species [14]. Piarroux et al [15] characterized a low copy, repetitive DNA sequence from *L. infantum *that was located exclusively at a large chromosome; this sequence was detected in many other *Leishmania *species. A repeated sequence with features of minisatellite DNA was characterized in the *L. infantum *genome; this element, called LiSTIR1, is 81-bp long and G+C rich and it was found interspersed at the subtelomeric regions of four chromosomes [16]. A 348-bp long element, designated LiR3, was found tandemly repeated within the non-transcribed spacers of the rDNA locus of *L. infantum *[17]. Conserved repeats, named LCTAS, have been characterized to be adjacent to telomeres in *L. braziliensis*, *L. major*, *L. mexicana *and *L. lainsoni *[18]. Also, several subtelomeric repetitive sequences have been characterized, showing to be responsible for size differences among the three *L. major *homologues for chromosome 1 [19]. Similar repeats have been found as tandemly arranged clusters at subtelomeric regions in chromosomes 1, 19 and 22 of *L. infantum*. Interestingly, these repeats are transcribed by RNA polymerase II into noncoding RNAs in a developmentally regulated manner [20]. Non-LTR retrotransposons are abundant in the genome of *T. brucei *and *T. cruzi*; by contrast, retroelements are absent from the *L. major *genome, where only remnants of degenerated *ingi*/L1Tc-related elements (or DIREs) are detectable (the *L. major *haploid genome contains 52 DIREs). Evolutionary analyses indicate that the trypanosomatid ancestor contained active transposable elements that have been retained in the genus *Trypanosoma*, but were lost in the *L. major *evolutionary line [21]. Recently, in an outstanding work, Bringaud et al [22] have found that the *L. major *contains two classes of short interspersed repeated sequences, SIDER1 (785 copies) and SIDER2 (1073 copies), which displays hallmarks of trypanosomatid retroposons. Members of the SIDER1 family show high sequence similarity with a conserved 450–550-bp element, located in the 3'UTR of several *Leishmania *amastigote-specific transcripts, that is implicated in stage-specific translational control [23,24]. SIDER2 elements, also located predominantly within 3'UTRs, have a demonstrated role in mRNA degradation [22]. Thus, it was postulated that *Leishmania *have recycled the retroposon remnants to regulatory sequences to globally modulate the expression of a number of genes [22].

In the course of studying repetitive DNA in the *L. infantum *genome, we identified and characterized a family of repeated sequences, which are interspersed along the different chromosomes. These sequence elements are present in different *Leishmania *species and, here, we show a detailed analysis of these elements in the *L. infantum *chromosomes 20 and 32, and in the *L. braziliensis *and *L. major *chromosome 32. During the preparation of this manuscript, the existence of this class of sequences in the *L. major *genome was reported [22], and, consequently, we adopted the proposed name (SIDER, Short Interspersed Degenerated Retroposon) for the elements identified in this work.

## Results

### Identification of a new family of repeated sequences in *L. infantum*

**Figure 1:**
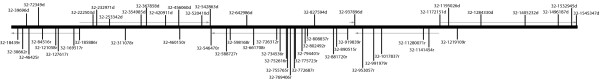
**Genomic organization of SIDER2 elements in the *L. infantum *chromosome 32**. The position and the orientation of the elements were deduced from the sequence of contig LinJ32_20070420_V3 [25]. The extent and directions of the transcriptional units are denoted by horizontal grey arrows. See table 1 for further details of the SIDER2 elements mapping to this chromosome.

As an approach to isolate and identify repetitive sequences in the *L. infantum *genome, we hybridized genomic DNA macroarrays of *L. infantum *(JPC strain) with labelled total genomic DNA of this parasite. A clone, named pGLi5-G8g, was selected for further analysis on the basis of its strong hybridization signal. Sequence analysis showed that the 2280-bp long insert locate on *L. infantum *chromosome 32 (EMBL accession number AM937229). However, the most striking observation, derived from the BLAST analysis, was that sequences, homologous to the 5'-end region of this clone, were also present in many additional locations in all the 36 *L. infantum *chromosomal contigs. A thoughtful search along the *L. infantum *chromosome 32 (contig LinJ32_20070420_V3; [25]), using iterative rounds of BLAST searches, led us to the identification up to 54 sequence elements. We named these elements as LiSIDER2s, following the nomenclature coined by Bringraud and coworkers in a recent publication describing the existence of this class of sequences in the *L. major *genome; SIDER stands for short interspersed degenerated retroposon [22]. The different LiSIDER2s found in chromosome 32 are listed in Table 1. These elements have two salient features: a size around 550 bp and a high G+C content (mean value 66.5%). Based on the *L. infantum *database (GeneDB), we have calculated that the G+C content for the *L. infantum *chromosome 32 is 58.8, very similar to the G+C content for the whole *L. infantum *genome [7]. All LiSIDER2 elements have G+C content higher than the mean value for the entire genome, and some of them exceed 70%. A physical location of the LiSIDER2 elements on *L. infantum *chromosome 32 is shown in figure 1. These elements were found in both plus and minus strands of the chromosome and they showed a quite even distribution along the chromosome. However, it is noticeable that most of the elements have the same orientation as the polycistronic transcription units in which they are located.

**Table 1:** SIDER2 elements present in the *L. infantum *chromosome 32 and comparison with their homologues in *L. major*

***L. infantum *SIDER2 element**				***L. major *SIDER2 homologue**		
**Name^a^**	**Subfamily**	**Size (%G+C)**	**79-bp signature^b^**	**Name^c^**	**Size (%G+C)**	**Sequence identity (%)**

32-18439r	A	560 (68.4)	2b	32-16072r	546 (67.4)	83
32-30662r	A	560 (68.9)	2b	32-28347r	549 (67.2)	82
32-39696d	A	570 (70.4)	2b	32-37464d	541 (64.3)	69
32-46425r	A	570 (70.0)	2a	32-44074r	547 (67.8)	81
32-72349d	A	568 (70.4)	2b	32-70108d	550 (69.3)	82
32-84516r	A	546 (70.5)	2b	32-82363r	550 (68.7)	81
32-121058r	A	550 (69.6)	2b	32-119100r	518 (64.5)	76
32-127617r	A	515 (69.7)	2b	32-125634r	515 (63.5)	74
32-169517r	B	564 (64.0)	2a	32-168081r	568 (64.1)	87
32-185886r	B	564 (64.2)	2a	32-184597r	569 (64.2)	87
32-222503d	C	594 (66.3)	2b	32-221358d	555 (68.7)	84
32-232971d	C	578 (67.0)	2b	32-231497d	582 (69.1)	85
32-253342d	C	578 (67.5)	2b	32-252118d	578 (68.9)	86
32-311078r		535 (61.7)	2b	32-308193r	601 (59.6)	79
32-354985d	D	586 (63.5)	2b	32-351852d	574 (62.5)	87
32-367858d	D	590 (63.6)	2b	32-364746d	574 (62.9)	86
32-420911d	E	540 (68.0)		32-413631d	561 (67.7)	84
32-456060d	E	556 (67.8)		32-448879d	566 (67.3)	85
32-460150r	E	555 (67.8)		32-452988r	562 (67.6)	86
32-520410d	F	661 (68.4)		32-515291d	663 (70.7)	78
32-542863d*	F	422 (68.5)		32-537679d	579 (71.7)	59
32-546470r	F	679 (68.3)		32-541585r	662 (71.0)	77
32-588727r	F	588 (66.7)		32-584100r	528 (70.6)	73
32-598168r	F	597 (68.2)		32-593579r	565 (69.4)	80
32-642986d*	F	461 (68.6)		32-638510d	542 (68.3)	62
32-661708r		552 (64.7)	2b	32-657337r	404 (62.8)	62
32-726312r	G	490 (66.9)	2b	32-722841r	453 (66.7)	83
32-734536r	G	489 (68.5)	2b	32-731206r	556 (66.7)	73
32-752616r	G	492 (67.9)	2b	32-749267r	540 (66.7)	78
32-755765r	G	491 (68.8)	2b	32-752467r	553 (66.2)	75
32-769406r		441 (61.0)		32-766145r	569 (57.8)	69
32-772687r		425 (60.9)		32-769534r	408 (60.5)	80
32-775723r*	H	265 (65.3)		32-772586r^d^	430 (66.5)	54
32-794401r	H	603 (63.2)	2b	32-790497r	577 (62.9)	86
32-802492r	H	550 (63.8)	2b	32-798632r	541 (64.5)	86
32-808837r	H	550 (64.0)	2b	32-804952r	541 (64.7)	87
32-827594d	H	591 (62.4)	2b	32-824177d	581 (63.0)	91
32-881720r	I	570 (66.7)	2b	32-877913r	581 (65.4)	90
32-890515r	I	573 (66.8)	2b	32-886987r	584 (65.2)	90
32-919839r	J	623 (70.0)		32-916263r	502 (69.5)	74
32-937896d	J	507 (70.6)		32-934324d	600 (69.7)	75
32-953057r	J	623 (70.0)		32-949068r	600 (69.5)	88
32-991979r	K	614 (65.3)	2b	32-988462r	594 (65.3)	86
32-1017837r	K	614 (65.2)	2b	32-1014737r	594 (65.3)	85
32-1128071r		506 (63.4)	2b	32-1125938r	519 (62.0)	88
32-1141454r		404 (60.4)		32-1167639d	367 (61.6)	52
32-1172151d	L	506 (67.8)		32-1169025d^d^	367 (70.6)	67
32-1195026d	L	602 (66.5)		32-1191646d	618 (68.0)	87
32-1219109r	L	602 (66.6)		32-1215657r	618 (67.8)	89
32-1284330d		458 (65.9)	2b	32-1278797d	559 (63.9)	74
32-1405232d		467 (64.9)	2b	32-1399746d	468 (63.9)	74
32-1496187d	M	497 (66.4)	2a	32-1509833d	411 (66.4)	60
32-1532945d*	M	365 (68.2)		32-1546424d	409 (65.8)	79
32-1545347d		513 (61.6)	2b	32-1558844d	457 (62.1)	68

^a^The elements are named with the chromosome number (i.e., 32) and the position in the chromosome, according to the contig LinJ32_20070420_V3 sequence; d and r denote that the element is located in the plus or minus DNA strand, respectively. The indicated nucleotide corresponds to either the start or end of the element, depending on its location in the plus or minus DNA strand, respectively.

^b^The presence of significant BLASTN matches with the "79-bp signature" LmSIDER2a (2a) or LmSIDER2b (2b) is indicated.

^c^The elements are named with the chromosome number (i.e., 32) and the position according to the contig LmjF32_01_20050601_V5.2 sequence; d and r denote that the element is located on the plus or minus strand, respectively.

*Truncated element.

**Figure 2:**
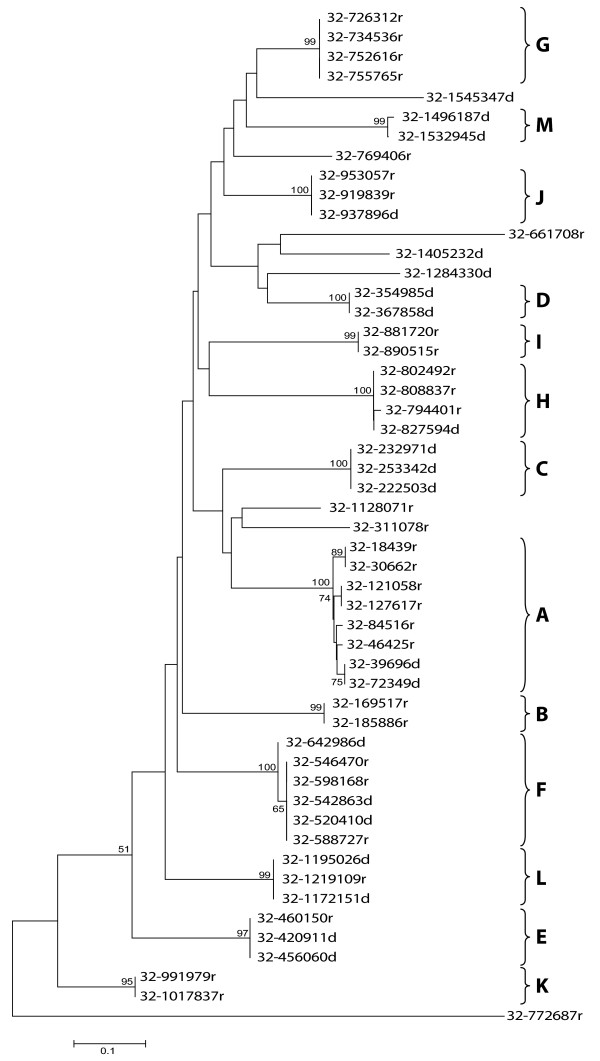
**Clustering by phylogenetic analysis of SIDER2 elements found in the *L. infantum *chromosome 32**. The alignment of the SIDER2 sequences was produced by ClustalW [see Additional file 1]. The phylogenetic tree was constructed with the program MEGA version 3.1 using the Neighbour-Joining method. The elements 32-775723r and 32-1141454r were excluded from the phylogenetic analysis because the program failed when calculating the Kimura distances for these sequences. Bootstrap values, expressed as percentages, were obtained from 1000 replicates. Only values higher than 50 are shown. The positions of the different subfamilies (A-M) are shown in the right margin. The scale represents number of changes per nucleotide.

Phylogenetic analyses (Fig. 2), based on the ClustalW alignment of the different LiSIDER2s [see Additional file 1], allowed us to group these elements into subfamilies. A subfamily was defined as a group of LiSIDER2s sharing sequence identity ≥ 85%. Thus, the 54 LiSIDER2s can be grouped into 13 subfamilies (named A to M), remaining 9 orphan elements (Table 1; Fig. 2). Remarkably, members of a given subfamily show a relationship of proximity, i.e. they are grouped close in the chromosome without intercalating non-familiar LiSIDER2s (Fig. 1). For example, elements of the subfamily A, composed of eight members, are located at the left hand of *L. infantum *chromosome 32 and no at other chromosomal regions.

**Figure 3:**
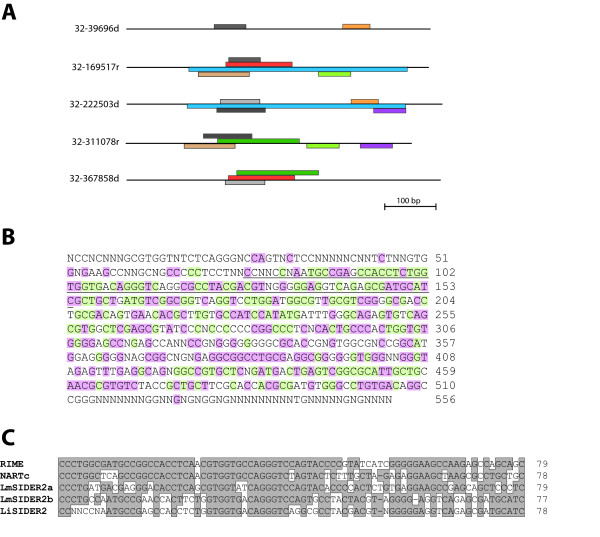
**Structural features of LiSIDER2 elements**. (A) The sequence homology between LiSIDER2s elements from different subfamilies is restricted to segments of varying sizes. Pairwise alignments among the indicated LiSIDER2s sequences were obtained using the BLAST two sequences tool available at the NCBI Web Page [45]. The retrieved matches are depicted by coloured boxes. (B) The 556-nucleotide long consensus sequence was derived from the ClustalW alignment of the 54 LiSIDER2 sequences identified in chromosome 32. A nucleotide was given a consensus status when was present in at least 50% of the sequences. Positions conserved in more than 60% of the sequences are shaded in red, and those conserved in more than 80% of the sequences are shaded in green. The underlined sequence corresponds to the "79-bp signature" sequence of trypanosomatid retroposons. (C) Comparison of the "79-bp signatures" present in the *T. brucei *RIME element, *T. cruzi *NARTc element, LmjSIDER2 elements (LmSIDER2a and LmSIDER2b), and LiSIDER2-32 consensus sequence. Conserved residues are boxed and shaded in grey; gaps (-) were introduced to maximize the alignments.

Another structural feature of LiSIDER2 elements, evidenced during the bioinformatics identification of the elements, was their composite nature. Thus, the elements from different subfamilies share only sequence blocks of variable size that are present in different combinations in each LiSIDER2. An example illustrating this observation is shown in Figure 3A. Nevertheless, a conserved consensus sequence for the LiSIDER2s can be derived from the alignment of the 54 elements present in chromosome 32 (Fig. 3B), suggesting a common origin for all elements. As suggested by Bringaud et al. [22], SIDER2 elements could be vestigial retroposons, derived from non-LTR retrotransposons of the *ingi*/L1Tc clade that remains active in the genomes of *T. brucei *and *T. cruzi *[21]. This hypothesis is based mainly upon the existence at the 5'-extremity of some LmjSIDER2 elements of the "79-bp signature", which constitutes the hallmark of trypanosomatid non-LTR retrotransposons and related elements [26]. Using the two "79-bp signatures" found in the *L. major *SIDER2 elements (LmSIDER2a and LmSIDER2b, [22]) for BLASTN searches, we found 35 matches in the *L. infantum *chromosome 32 sequence. Interestingly, 34 out of the 35 matches were coincident with the location of LiSIDER2 elements, indicating that this is not a fortuitous association. Thus, 34 (63%) out of the 54 SIDER2 elements, present in *L. infantum *chromosome 32, have a distinguishable "79-bp signature" that invariantly is located at, or close to, the 5'end of the element. For most of the LiSIDER2, the "79-bp signature" was found to be more similar to the LmSIDER2b sequence than to the LmSIDER2a one (Table 1). A comparison of the consensus "79-bp signature" present in the LiSIDER2s with that existing in other trypanosomatid elements is shown in figure 3C. For some LmjSIDER2 elements (18.9%), the presence of putative target site duplication (TSD) was noticed by Bringaud and co-workers [22]. However, after inspection of sequences immediately upstream and downstream of the different LiSIDER2s in chromosome 32, we did not find clear TSD sequences, even though when members of a subfamily were separately analyzed. Also, the presence of short adenosine-rich stretches was described at the 3'-end of some of the LmjSIDER2 elements. In the characterized LiSIDER2, adenosine runs were found to be present in about 28% of the elements, either at the 3'-end or in close proximity to it.

**Figure 4:**
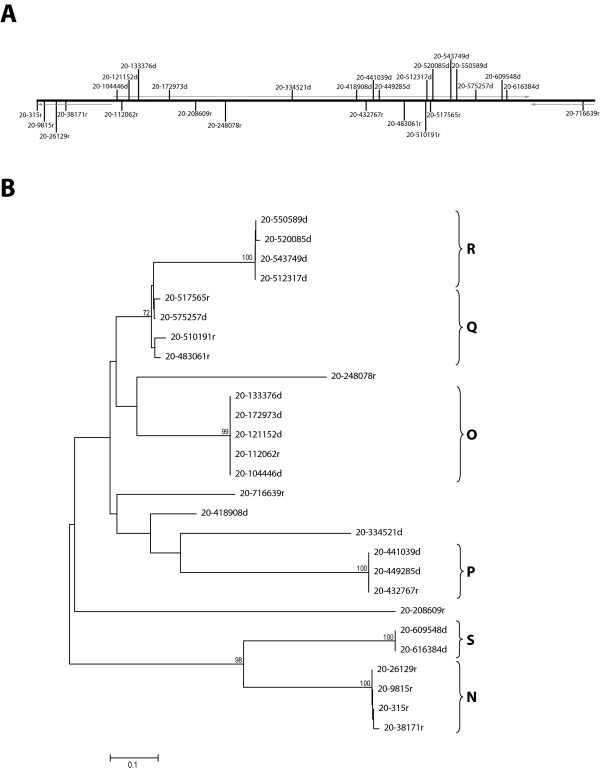
**Analysis of SIDER2 elements of the *L. infantum *chromosome 20**. (A) Genomic organization of LiSIDER2 elements, derived from the sequence of contig LinJ20_20070420_V3 [25]. (B) Phylogenetic tree of SIDER2 elements found in the *L. infantum *chromosome 20. Bootstrap values, expressed as percentages, were obtained from 1000 replicates (see legend to figure 2 for further details). The positions of the different subfamilies (N-S) are shown in the right margin. The scale represents number of changes per nucleotide.

In order to know whether or not this peculiar organization of LiSIDER2 elements is shared by the elements located in other *L. infantum *chromosomes, we carried out a systematic search of LiSIDER2s along the chromosome 20. We chose this chromosome, because we realized that sequences similar to LiSIDER2s had been previously described in the homologue chromosome in *L. major *[27]. As shown in Table 2, 27 elements were identified in the *L. infantum *chromosome 20. Similarly, these LiSIDER2s were found to have G+C-rich sequences, to have a size around 500-bp, and can be grouped in subfamilies according to sequence homology (N to S). In chromosome 20, we found that members of subfamilies Q and R are intercalated (Fig. 4A); however, it should be noted that these subfamilies are closely related each other in sequence (Fig. 4B; [see Additional file 2]). Another relevant finding was that two LiSIDER2s, which constitute subfamily S (Table 2), have an uncommon size (1270-bp), being the SIDER2-homologue region located at the 3'-end half of these elements. As occurred with LiSIDER2s of chromosome 32, most of LiSIDER2s in chromosome 20 are in the same orientation as the transcriptional units (Fig. 4A). Furthermore, 15 out of the 27 (56%) LiSIDER2-20 have a distinctive "79-bp signature" (Table 2).

**Table 2:** SIDER elements present in the *L. infantum *chromosome 20

**Name^a^**	**Subfamily**	**Size (%GC)**	**79-bp signature^b^**
20-315r	N	477 (66.3)	2b
20-9815r	N	554 (66.3)	2b
20-26129r	N	526 (65.8)	2b
20-38171r*	N	356 (67.4)	
20-104446d	O	576 (65.8)	2a
20-112062r	O	571 (65.5)	2a
20-121152d*	O	425 (65.2)	
20-133376d	O	661 (64.2)	2a
20-172973d	O	651 (64.7)	2a
20-208609r		384 (63.8)	2b
20-248078r		378 (61.4)	2b
20-334521d		221 (58.8)	
20-418908d		466 (65.7)	2a
20-432767r	P	561 (65.4)	
20-441039d	P	592 (65.0)	
20-449285d	P	603 (65.2)	
20-483061r	Q	466 (67.4)	
20-510191r*	Q	253 (70.0)	
20-512317d	R	451 (66.3)	2b
20-517565r	Q	334 (71.9)	
20-520085d	R	444 (66.4)	2b
20-543749d	R	455 (65.5)	2b
20-550589d	R	455 (65.5)	2b
20-575257d	Q	468 (68.6)	2b
20-609548d	S	1270 (70.0)	
20-616384d	S	1270 (69.8)	
20-716639r		556 (58.6)	

^a^The elements are named with the chromosome number (i.e., 32) and the position in the chromosome, according to the contig LinJ20_20070420_V3 sequence; d and r denote that the element is located in the plus or minus DNA strand, respectively.

^b^The presence of significant BLASTN matches with the "79-bp signature" LmSIDER2a (2a) or LmSIDER2b (2b) is indicated.

*Truncated element.

As deduced from BLAST analyses (data not shown), the rest of *L. infantum *chromosomes must be also populated by LiSIDER2 elements showing similar features as those described in chromosomes 20 and 32. Taking into account both the chromosomal size and the number of SIDER2s found in *L. infantum *chromosomes 20 and 32, we estimated that the *L. infantum *haploid content of SIDER2s would be around 1150 copies. This estimation is in agreement with the determination of 1073 copies of LmjSIDER2 in the *L. major *genome [22].

### Sequences homologous to LiSIDER2s are also present in the genome of other *Leishmania *species

Since the complete sequence of the *L. major *is known [5], we carried out the same bioinformatics analysis on the *L. major *database using as query sequences the different LiSIDER2 elements found in the *L. infantum *chromosome 32 (Table 1). In all cases, the best scores were observed with sequences located in the *L. major *chromosome 32. Table 1 summarizes molecular features of the SIDER2s found in the *L. major *chromosome 32. Remarkably, it was observed an extremely high conservation, both in sequence and genomic location, of the SIDER2s found in the *L. major *and *L. infantum *chromosomes 32. To avoid confusion, following the genetic nomenclature directions for kinetoplastids [28], we named the *L. major *elements as LmjSIDER2. In an independent study, Bringaud et al [22] identified 55 SIDER2s elements in the *L. major *chromosome. Except for small variations in the coordinates, there was a total correspondence between the 54 elements identified by us (Table 1) and those identified by Bringaud and colleagues. Our analysis failed to find the LmjSIDER2 starting at position 626445 [22].

Recently, the completion of the *L. braziliensis *genome sequence has been announced [6], and we considered of interest to search for the existence of these elements in this species. First analyses indicated that SIDER2 sequences indeed exist in the *L. braziliensis *genome, but the distribution of the elements in the chromosome 32 was not conserved regarding the conspicuous conservation of SIDER2 elements that exists between *L. infantum *and *L. major *chromosome 32. Thus, BLAST searches using the LiSIDER2 sequences from chromosome 32 showed that best scores were not with sequences from *L. braziliensis *chromosome 32. Rather, bestfits for each LiSIDER2-32 sequence were found with sequences distributed among the different *L. braziliensis *chromosomes, indicating that SIDER2s are not chromosome specific for all *Leishmania *species. However, the intrachromosomal organization of these elements in the *L. braziliensis *genome showed features similar to that found in the other two *Leishmania *species. Thus, most of the 48 LbSIDER2 elements, which were identified in the *L. braziliensis *chromosome 32, can be grouped, according to sequence homology, in subfamilies (a to k), whose members also show a relationship of proximity (Table 3).

**Table 3:** SIDER elements present in the *L. braziliensis *chromosome 32

**Name^a^**	**Subfamily**	**Size (%GC)**
32-21338r	a	507 (70.8)
32-33564r	a	429 (70.2)
32-34297r	a	531 (69.3)
32-46283d	a	503 (69.8)
32-52976r	a	401 (70.3)
32-56914r	a	532 (69.2)
32-82781d	a	526 (69.6)
32-190328r		438 (55.7)
32-218978d	a	465 (72.5)
32-257472d		565 (66.4)
32-335085r	b	422 (66.4)
32-356555d	b	388 (67.3)
32-380245d	b	422 (67.5)
32-397315d	b	340 (71.2)
32-447556d		499 (57.7)
32-528182d	c	459 (66.2)
32-558759d	c	629 (63.3)
32-581122r	c	634 (64.2)
32-632143r		585 (60.3)
32-640725r		483 (56.9)
32-693667d	d	983 (57.8)
32-695383d*	d	289 (60.2)
32-716506r	d	981 (58.0)
32-789725r	e	555 (70.6)
32-799255r	e	550 (71.1)
32-816512r	e	550 (71.1)
32-819658r	e	550 (70.7)
32-836061r	e	569 (71.9)
32-839556r	e	565 (71.3)
32-842876r	e	565 (71.7)
32-850687r	e	546 (71.4)
32-867036r	f	508 (67.7)
32-875142r	f	486 (67.7)
32-904165d		442 (53.9)
32-965250r		447 (57.1)
32-977561r		540 (60.2)
32-1033093d	g	570 (68.6)
32-1047782r	g	571 (67.6)
32-1091401r	h	604 (67.1)
32-1121626r	h	605 (67.1)
32-1308414d	i	523 (64.4)
32-1332630r	i	528 (64.2)
32-1396673d	i	496 (64.5)
32-1539141d	j	436 (64.2)
32-1547986d	j	436 (64.2)
32-1558134d*	j	173 (68.8)
32-1702406d	k	443 (63.9)
32-1714067d	k	443 (64.3)

^a^The elements are named with the chromosome number (i.e., 32) and the position according the contig LbrM32 (version 2.0) sequence; d and r denote that the element is located on the plus or minus strand, respectively.

*Truncated element.

In addition to the analysis of *Leishmania *genome databases, we performed searches looking for SIDER2 homologue elements in general databases (EMBL and GenBank). A large number of entries were retrieved; however, all entries contained *Leishmania *sequences and homologous sequences were not found in other organisms, with an intriguing exception. Thus, we found a significant homology between LiSIDER-32-121058d and the EMBL entry with accession number AM094505, which corresponds to a *Lutzomyia longipalpis *EST clone NSFM-162h01. Remarkably, this sandfly species acts as *Leishmania *transmission vector. On the other hand, BLAST searches in the *T. cruzi *and *T. brucei *genome databases (GeneDB) yielded not results, indicating that these elements are specific for the *Leishmania *genus. Among the retrieved entries from the EMBL and GenBank databases, there are sequences derived from *L. amazonensis *(U70540, AB029444, AY427440S3, DQ092336), *L. braziliensis *(DQ092335), *L. donovani *(Z94053, AC093553, AF067495, AF109296, AY028171, AY791850, DQ092337), *L. hoogstraali *(DQ092338), *L. infantum *(M93416, L27052, AJ628942, AM118098), *L. major *(Z54138, AY227807, AY328521, AY491007), *L. mexicana *(Z46971, AF350492, AJ131960, AJ427448, AJ548776, AY170465), and *L. tarentolae *(AY842846). Remarkably, there exist many entries corresponding to *L. chagasi *cDNAs (CV669830, CV667316, CV670663, CV663048, CV669851, CV669636, CV662260, CV666468, CV669797, CV666868, CV664167, CV669564, CV665051, CV663324, CV668078, CV668316) that have significant BLAST scores with SIDER2 sequences.

**Figure 5:**
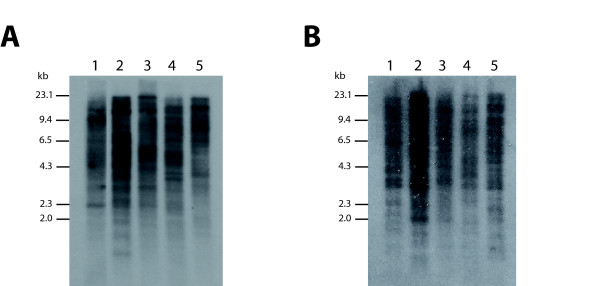
**Southern blot analyses of the genomic distribution of SIDER2 sequences in different *Leishmania *species**. Genomic DNA (*Sal*I-digested) of *L. infantum *(lane 1), *L. major *(lane 2), *L. tropica *(lane 3), *L. mexicana *(lane 4), and *L. braziliensis *(lane 5) was analyzed by Southern blotting using as probes either LiSIDER2-32-121058r (panel A) or LiSIDER2-20-575257d (panel B).

The bioinformatics analysis indicated that SIDER2 elements are widespread among the different *Leishmania *species. In order to obtain experimental evidence, Southern blots containing *Sal*I-digested genomic DNA from *L. infantum*, *L. major*, *L. tropica*, *L. mexicana *and *L. braziliensis *were probed with two different LiSIDER2s, LiSIDER2-32-121058r and LiSIDER2-20-575257d (Fig. 5). Complex hybridization patterns were obtained with each one of the probes, confirming the repeated nature of the SIDER2 elements. The hybridizations patterns are also in agreement with a scattered distribution of these elements in the *Leishmania *genome. Although, differences were observed in the signal intensity of particular bands among the different *Leishmania *species, the global hybridization signal was found very similar, suggesting that a similar number of SIDER2s elements must be present in the different species tested.

## Discussion

In a recent work, Bringaud and co-workers [22] identified two related families of small elements by a bioinformatics analysis of the *L. major *genome sequence using as bait the "79-bp signature" common to trypanosomatid retroposons [21]. These families, named LmSIDER1 and LmSIDER2, contain 785 and 1073 copies per haploid genome, respectively. These authors raised a compelling hypothesis: these elements are extinct retroposons that have been recycled to accomplish regulatory functions for gene expression in *Leishmania*. Here, we describe the existence of this class of elements in the genome of *L. infantum *and other *Leishmania *species. The starting point of our work was the isolation from a macroarray of a clone showing strong hybridization signal when *L. infantum *total DNA was used as probe. Sequencing of this clone indicated that it contains a genomic fragment of chromosome 32, but the bioinformatics analyses showed also that this clone would contain a repeated sequence because significant homology with different sequences located on the different *L. infantum *chromosomes was observed. After a thoughtful analysis, we identified a total of 54 elements in the *L. infantum *chromosome 32 and 27 elements in the chromosome 20. Sequence comparisons analysis between the repeated elements identified in this work with those described by Bringaud and co-workers in *L. major*, suggest that the elements described here belong to the SIDER2 family [22].

SIDER2 elements show outstanding features regarding genomic organization ([22]; this work): i) the elements are abundant and distributed along the different chromosomes in all *Leishmania *species; ii) the elements constitutes subfamilies related in sequence and genomic vicinity; iii) the *L. major *and *L. infantum *SIDER2s are highly conserved both in sequence and chromosomal location. This degree of conservation in chromosomal location is not maintained between the *L. major*/*L. infantum *and *L. braziliensis *SIDER2s (at least for chromosome 32). It should be kept in mind that *L. braziliensis *is the most genetically and biologically divergent of the three species analyzed for this study [29]. A remarkable difference, which may be related with the variations in genomic distribution of SIDER2 elements among the *Leishmania *species, is that *L. braziliensis *possesses potentially active retrotransposons that are absent in the other two *Leishmania *species [6].

Accumulating data from different organisms do indicate that mobile elements and non-coding repetitive sequences are important elements in a genome and may be playing functional roles that vary from control of gene expression to chromosomal organization [1,3]. In this regard, the sequence features and genomic organization of SIDER2 elements are suggestive of relevant functional roles, but what kind of function can they be playing? The search for these elements within coding regions in *L. infantum *predicted genes indicates that no SIDER2 sequences are in coding region. The sole exception to this rule is the *L. infantum *database entry LinJ10_V3.1340, which contains sequence homology to SIDER2s. However, this entry is considered as pseudogene, since its sequence contains several in-frame stop codons. Remarkably, this putative pseudogene shows high sequence conservation with genes containing uninterrupted ORF in other kinetoplatids: LmjF10.1225 (*L. major*), LbrM10_V2.1350 (*L. braziliensis*), Tc00.1047053506153.6 (*Trypanosoma cruzi*) and Tb927.8.4690 (T. brucei). In spite of this particular finding, as overall conclusion, it must be stated that SIDER2 elements are rare in coding sequences.

On the other hand, several lines of evidence suggest that SIDER2 elements are frequently found in untranslated regions (UTRs) of genes, mainly 3'UTRs. Using both bioinformatics and experimental approaches, Bringaud et al. [22] demonstrated that SIDER2 elements are present in 3-UTRs of many different genes. Furthermore, these authors showed experimental evidence that SIDER2 sequences are promoting downregulation of mRNA steady state levels. In addition, our database analyses showed that several *L. chagasi *cDNAs have SIDER2 sequences, reinforcing the idea that these elements are frequently found in UTRs of mRNAs, playing putative regulatory role in gene expression.

**Figure 6:**
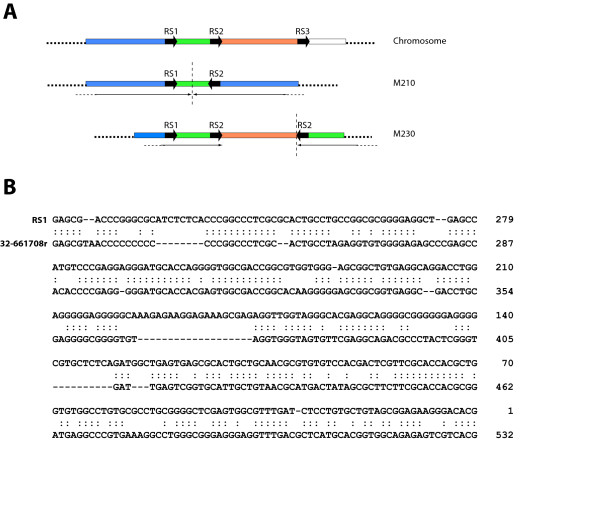
**SIDER2 sequences are found in linear inverted DNA amplicons of *Leishmania***. (A) Schematic representation showing the location of the repeated sequences RS1, RS2 and RS3 in the M210 and M230 *L. major *amplicons and in the chromosome source (depicted according to data shown in reference [32]). The location and orientation of the RS1, RS2 and RS3 repeats are indicated by arrows. The rearrangement points are indicated by discontinuous, vertical lines. The extent and orientation of the inversions are indicated by horizontal arrows. The drawing is not to scale. (B) Alignment of RS1 and LiSIDER2-32-661708r sequences. Symbol (:) indicates identical bases in both sequences. Gaps (-) were introduced to maximize the alignment.

Extrachromosomal DNA amplifications are commonly observed in different *Leishmania *species either after drug pressure or even in natural isolates [30,31]. When parasites are subjected to selective stresses, appropriate genomic DNA regions, containing flanking repeats, are amplified as extrachromosomal structures. According to the Beverley's model for explaining DNA amplification phenomena, the *Leishmania *genome should contain amplification-prone cassettes [30]. Thus, the genomic organization of SIDER2 elements in the *Leishmania *chromosomes (see Figs. 1 and 4) could be related with the amplification mechanism. Interestingly, another prediction of the model is the existence of two types of cassettes, those flanked by direct repeats and those flanked by inverted repeats. SIDER2 elements are found in both direct and inverted orientations, which further suggest their possible implication in *Leishmania *DNA amplification. In order to find additional cues supporting this idea, we looked for SIDER2 sequences in characterized DNA amplification structures of *Leishmania*. Remarkably, SIDER2 related sequences were found in several GenBank and EMBL entries corresponding to *Leishmania *DNA amplification structures. For example, three repeated sequences (RS1, RS2 and RS3) were identified in close proximity to the recombination points of extrachromosomal linear DNA amplicons M210 and M230 of *L. major *[32]. Schematic drawings for M210 and M230 amplicons, and for the genomic region of the source chromosome are depicted in figure 6A. Both amplicons have an inverted repeat structure, and the inversion occurred between repeats RS1 and RS2 for M210, and between RS2 and RS3 for M230. The three repeated sequences are 374-bp in size and show a high level of sequence identity (98%) [32]. These repeated sequences have a remarkable homology with LiSIDER2 sequences (figure 6B), suggesting that they are members of an LmjSIDER2 subfamily. In other example, the repeated sequences, postulated to be involved in the formation of a linear amplicon in *L. tarentolae *[33], also share significant sequence homology with LiSIDER2 elements. These data suggest that indeed SIDER2 elements could be involved in the generation of some *Leishmania *extrachromosomal amplification.

Our search on GenBank and EMBL databases showed the existence of SIDER2 elements in other relevant *Leishmania *genomic regions. For example, homology to SIDER2 sequences is found in a 44-kb genomic region, which was involved in mitotic stability of extrachromoses in *L. donovani *[34]. To date, the DNA elements participating in the chromosomal replication and segregation processes are largely unknown in *Leishmania *and other trypanosomatids. The difficulty to uncover the centromeres in trypanosomatids could be pointing to the existence of holocentric chromosomes that are characterized by the presence of a diffuse or nonlocalized centromere during mitosis [35]. In this scenario, SIDER2 elements should be considered as candidates for centromeric sequences. This hypothesis is based on two features of SIDER2 elements: they are distributed regularly along the chromosomes (Figs. 1 and 4) and they have G+C-rich sequences. Richness in G+C-sequences is observed in centromeres and pericentromic regions of many organisms [36]. Also, it is noticeable the existence, within the SIDER2 sequences, of G-rich tracts that are known for their propensity to form G-quadruplex DNA structures [37].

Finally, the presence of the "79-bp signature" in a large proportion of the SIDER2 elements may be suggestive of a transcriptional role for this class of repeats. In a previous report, we have demonstrated that the "79-bp signature" (also named Pr77), derived from *T. cruzi *L1Tc non-LTR retrotransposon has a RNA-pol II-dependent promoter that strongly activates gene transcription [38]. In this context, it may be postulated that SIDER2s bearing the "79-bp signature" could be acting as RNA-pol II recruiting points to enhance the transcriptional active at some chromosomal regions.

## Conclusion

In this study, we describe several features of a family of novel repeated elements (named SIDER2) that are interspersed along the different chromosomes and present in all *Leishmania *species. We show an in-depth analysis of these elements in the *L. infantum *chromosomes 20 and 32, and in the *L. major *and *L. braziliensis *chromosomes 32. Apart from their proved role in post-transcriptional regulation of gene expression in *Leishmania*, our analyses suggest that SIDER2 elements could be involved in DNA amplification phenomena and, perhaps, they can represent centromeric sequences of holocentric chromosomes. In summary, SIDER2 elements constitute a relevant piece of the *Leishmania *genome organization, and this work provides a framework for investigating the functions of these sequences.

## Methods

### Parasites and DNA isolation

*L. infantum *JPC strain (MCAN/ES/98/LLM-724, clone M5) was used for arrays construction. For Southern blot analysis, the following *Leishmania *species were used: *L. tropica *(MHOM/SU/74/K-27), *L. mexicana *(MNYC/BZ/62/M-379), *L. amazonensis *(IFLA/BR/67/PH-8), *L. braziliensis *(MHOM/BR/75/M-2904) and *L. major *(MHOM/IL/80/Friedlin). Promastigote forms were cultured *in vitro *at 26°C in RPMI 1640 medium (Sigma), supplemented with 10% heat-inactivated foetal calf serum (Sigma).

Genomic DNA was prepared from 2 × 10^8 ^promastigotes. After washing with phosphate-buffered saline (PBS), cells were suspended in 500 μl of lysis buffer (0.15 M NaCl, 0.1 M EDTA (pH 8.0) and 0.5% SDS). Afterwards, proteinase K was added to a final concentration of 0.1 mg/ml. After incubation for 30 min at 50°C, samples were extracted sequentially with phenol, a phenol-chloroform-isoamyl alcohol (25:24:1) mixture and a chloroform-isoamyl alcohol (24:1) mixture. After adding 0.1 volumes of 3 M sodium acetate and 2.5 volumes of cold ethanol, DNA was collected by centrifugation. The pellet was suspended in 200 μl of Te buffer (10 mM Tris-HCl and 0.1 mM EDTA, pH 8.0) and incubated with RNAse A (20 μg/ml final concentration) for 30 min at 37°C. Afterwards, DNA samples were extracted with a phenol-chloroform-isoamyl alcohol (25:24:1) mixture, and DNA precipitated by addition of 0.5 volumes of 7.5 M ammonium acetate and 2 volumes of cold ethanol. Finally, DNA was suspended in 100 μl of Te buffer.

### *L. infantum *genomic arrays

Genomic DNA macroarrays were constructed as previously described [39]. Briefly, a genomic library of *Sau*3AI DNA fragments (4-kb average size) was constructed in pBluescript KS plasmid (Promega). DNA from individual colonies was prepared using the Perfectprep Plasmid 96 Vac kit (Eppendorf) and the BIOMEK 2000 robot (Beckam). DNA from 575 different clones was spotted in triplicate onto positively charged nylon membranes (Schleicher and Schuell) by NewBioTechnic (Sevilla, Spain).

Before hybridizations, macroarray membranes were washed with 0.5 M phosphate buffer (pH 7.2) and incubated for 2 h at 65°C in 20 ml of hybridization solution (0.5 M phosphate buffer (pH 7.2), 7% SDS and 1 mM EDTA). For hybridization, 350 ng of *L. infantum *genomic DNA were labelled by nick-translation using 50 μCi of [α-^32^P]dCTP (3000 Ci/mmole; Amersham) and standard methods [40]. The labelled-DNA was added to the hybridization solution, and membranes were further incubated for 12 h at 65°C. Afterwards, membranes were washed three times with washing solution (40 mM phosphate buffer (pH 7.2) and 0.1% SDS) for 20 min at 65°C. Radioactive signals were analyzed by a Phosphorimager (Fuji BAS-1500).

### DNA sequencing of clone pGLi5-G8g

Both strands of the insert of clone pGLi5-G8g were sequenced using an automated sequencer (ABI Prism 3730; Applied Biosystems) by the Genomics Unit of the Parque Científico de Madrid (SIDI-UAM). Nucleotide sequence of this clone has been deposited at European Molecular Biology Laboratory (EMBL/EBI) nucleotide sequence database under accession number AM937229.

### Identification of SIDER2 sequence elements in *Leishmania *databases

An initial BLASTN search of the *L. infantum *database [25] using the sequence of clone pGLi5-G8g showed that this clone contains a genomic region from chromosome 32. However, a subregion of approximately 550-bp was found to be widespread along the *L. infantum *genome. For the identification of these repeated sequences, now called LiSIDER2 elements, an iterative process was followed. For a given chromosome, sequence blocks showing sequence identity ≥ 60% and length ≥ 100 nucleotides with pGLi5-G8g sequence were considered for further analysis. Selected sequences (plus surrounding upstream and downstream sequences) were aligned using ClustalW. The clustering of sequences into subfamilies was carried out by phylogenetic analysis (see below). To determine the extent of the elements belonging to a given subfamily, the particular sequences were aligned with ClustalW and the extremities determined by visual inspection of the alignment. A subfamily was defined as a group of elements sharing sequence identity ≥ 85%. When the size of an element was clearly different to the medium size for the elements of the subfamily, it was considered as truncated element. Each time a subfamily was identify, the sequence of the longest member of the subfamily was used to perform additional BLASTN searches in the *Leishmania *databases (contig sequences, [25]), the retrieved sequences (if new) were aligned as indicated above; the process was repeated until no new sequences were obtained. Finally, the remaining matches, non-assigned to any subfamily, were considered as SIDER2 "orphan" elements. Given the complexity of the identification process, we restricted the analyses to contigs for chromosomes 32 (LinJ32_20070420_V3) and 20 (LinJ20_20070420_V3).

Identification of SIDER2 elements in *L. major *and *L. braziliensis *databases [25] was performed by BLASTN searches using representative members for the LiSIDER2 subfamilies and the orphans LiSIDER2 elements of *L. infantum *chromosome 32. Each time a homologous sequence was retrieved, it was used to perform additional BLASTN searches in the database. The size and genomic positions for the different LmjSIDER2 or LbSIDER2 elements were determined by sequence alignments using ClustalW and manual corrections. Again, we restricted our analysis to chromosomes 32 of *L. major *(LmjF32_01_20050601_V5.2) and *L. braziliensis *(LbrM32, version 2.0).

### Multiple alignments and phylogenetic trees

The complete LiSIDER2 sequences were aligned using the default options of ClustalW2 [41]. The resulting alignments were used to perform phylogenetic analysis conducted with the program MEGA version 3.1 [42] using the Neighbour-Joining method and default parameters.

### Other databases mining

The different LiSIDER2 elements found in *L. infantum *chromosome 32 were used for BLASTN searches in *T. brucei *and *T. cruzi *databases [25]. Also, BLAST searches were performed in GenBank and EMBL databases.

### DNA probes and Southern blot analysis

LiSIDER2-32-121058r and LiSIDER2-20-575257d elements were PCR amplified using as template genomic DNA from *L. infantum *JPC strain. As primers, the following oligonucleotides were used: LiRS-32-Ad (5'-CCGCCCCGAAATATAAGT-3') and LiRS-32-Ar (5'-GCCTCCATGCGCGGTGTC-3') for LiSIDER2-32-121058r; 20R-d (5'-CCACATCGCGCGTGGCGC-3') and 20R-r (5'-TGACGTGTGGACCCCGCT-3') for LiSIDER2-20-575257d. The amplification products were cloned into the pCR2.1 vector (Invitrogen), yielding clones pLiRS-32A (LiSIDER2-32-121058r) and pLiRS-20Q (LiSIDER2-20-575257d). The authenticity of clones and the fidelity of the PCR-amplification were verified by nucleotide sequencing.

For Southern blot analysis, 1 μg of total DNA from the different *Leishmania *species was digested with the *Sal*I restriction enzyme and electrophoresed on 0.8% agarose cells. After ethidium bromide visualization, DNA was transferred to nylon membranes (Hybond-N, Amersham) by standard methods [43]. For probe preparations, *Eco*RI-inserts of clones pLiRS-32A and pLiRS-20Q were labelled with [α-^32^P]dCTP by nick-translation [43]. Hybridizations were performed as reported earlier [44].

## Authors' contributions

CF, MCL and MCT carried out the different steps of macroarrays construction. CF performed hybridization of macroarrays, PCR amplification and Southern blotting. JMR conceived the project, supervised the experiments and performed the bioinformatics analyses. MCL and MCT helped to draft the manuscript. CF prepared the final version of figures. JMR wrote the final version of the manuscript. All authors have read and approved the final manuscript.

## Supplementary Material

Additional file 1 ClustalW2 alignment of the 54 LiSIDER2 sequences present in the *L. infantum *chromosome 32. Click here for file

Additional file 2 ClustalW2 alignment of the 27 LiSIDER2 sequences present in the *L. infantum *chromosome 20. Click here for file
