# Supplementary material for: The SIDER2 elements, interspersed repeated sequences that populate the Leishmania genomes, constitute subfamilies showing chromosomal proximity relationship
Source: BMC Genomics. 2008 Jun 2;9:263. doi: 10.1186/1471-2164-9-263 (PMC2424063; doi:10.1186/1471-2164-9-263)
Supplement: Additional file 1 — ClustalW2 alignment of the 54 LiSIDER2 sequences present in the L. infantum chromosome 32. [file 1471-2164-9-263-S1.doc]

**ClustalW2 Results**

Results of search

Number of sequences 54

Alignment score 2209867

Sequence format Pearson

LiSIDER2-32-354985d .......... .......... .......... .......... .CCCGGGTGA

LiSIDER2-32-367858d .......... .......... .......... .......... .CCCGGGTGA

LiSIDER2-32-661708r .......... .......... .......... .......... ..........

LiSIDER2-32-1405232d .......... .......... .......... .......... ..........

LiSIDER2-32-169517r .......... .......... .......... .......... ...CTCTTGC

LiSIDER2-32-185886r .......... .......... .......... .......... ...CTCTTGC

LiSIDER2-32-420911d .......... .......... .......... .......... ..........

LiSIDER2-32-460150r .......... .......... .......... .......... ..........

LiSIDER2-32-456060d .......... .......... .......... .......... ..........

LiSIDER2-32-991979r .......... .......... .......... .......... ..........

LiSIDER2-32-1017837r .......... .......... .......... .......... ..........

LiSIDER2-32-18439r .......... .......... .......... .......... ..........

LiSIDER2-32-30662r .......... .......... .......... .......... ..........

LiSIDER2-32-39696d .......... .......... .......... .......... ..........

LiSIDER2-32-72349d .......... .......... .......... .......... ..........

LiSIDER2-32-46425r .......... .......... .......... .......... ..........

LiSIDER2-32-84516r .......... .......... .......... .......... ..........

LiSIDER2-32-121058r .......... .......... .......... .......... ..........

LiSIDER2-32-127617r .......... .......... .......... .......... ..........

LiSIDER2-32-1128071r .......... .......... .......... .......... ..........

LiSIDER2-32-222503d .......... .......... .......... CGTCTTCCTC TCTCATCCTG

LiSIDER2-32-232971d .......... .......... .......... .......... .....TCCTG

LiSIDER2-32-253342d .......... .......... .......... .......... .....TCCTG

LiSIDER2-32-311078r .......... .......... .......... .......... ..........

LiSIDER2-32-1195026d TAAGAGGATC GATAAGCAGT TATTTTTGTT TTCCTCTCCT TTCTCTCAAA

LiSIDER2-32-1219109r TAACAGACTC GATAAGCAGT TATTTTTGTT TTCCTCTCCT TTCTCTCAAA

LiSIDER2-32-1172151d .......... .......... .......... .......... ..........

LiSIDER2-32-542863d .......... .......... ....TCTCCT TTCTCATGAC CGGGCACCCA

LiSIDER2-32-546470r .......... .......... ....TCTCCT CTCTCATGAC CGGGCACCCA

LiSIDER2-32-520410d .......... .......... .......... .....ATGAC CGGGCACCCA

LiSIDER2-32-588727r .......... .......... .......... .......... ..........

LiSIDER2-32-642986d .......... .......... .......... .......... ..........

LiSIDER2-32-598168r .......... .......... .......... .......... ..........

LiSIDER2-32-919839r .......... ..CCCGAACG ACGGAGGTGG AACGCCCCAG TGCATGGCAT

LiSIDER2-32-953057r .......... ..CCCGAACG ACGGAGGTGG AACGCCCCAG TGCATGGCAT

LiSIDER2-32-937896d .......... .......... .......... .......... ..........

LiSIDER2-32-726312r .......... .......... .......... .......... ..........

LiSIDER2-32-752616r .......... .......... .......... .......... ..........

LiSIDER2-32-734536r .......... .......... .......... .......... ..........

LiSIDER2-32-755765r .......... .......... .......... .......... ..........

LiSIDER2-32-1284330d .......... .......... .......... .......... ..........

LiSIDER2-32-775723r .......... .......... .......... .......... ..........

LiSIDER2-32-794401r .......... .......... .......... .......... ..........

LiSIDER2-32-802492r .......... .......... .......... .......... ..........

LiSIDER2-32-827594d .......... .......... .......... .......... ..........

LiSIDER2-32-808837r .......... .......... .......... .......... ..........

LiSIDER2-32-769406r .......... .......... .......... .......... ..........

LiSIDER2-32-881720r .......... ...TTTTGTG TTCCCGTTTT CTCTTGCCTT CCCGAATGAC

LiSIDER2-32-890515r .......... ...TTTTGTG TTCCCGTTTT CTCTTGCCTT CCCGAATGAC

LiSIDER2-32-1496187d ......AGCG CGCGTTTCTG CTGTCTCTCT TGCCGCCTTC CCCCGATGAC

LiSIDER2-32-1532945d .......... .......... .......... .......... ..........

LiSIDER2-32-1545347d .......... .......... .......... .......... ......TGAC

LiSIDER2-32-772687r .......... .......... .......... .......... ..........

LiSIDER2-32-1141454r .......... .......... .......... .......... ..........

LiSIDER2-32-354985d CGGAGGGGGG .ACCTCAATG TGTGGTATCT GAGGGCGCGG CGCACCCCCG

LiSIDER2-32-367858d CGGAGGGGGG GACCTCAATG TGTGGTATCT GAGGGCGCGG CGCACCCCCG

LiSIDER2-32-661708r .......... .......... .......... .......... ....GCACAC

LiSIDER2-32-1405232d .......... .......... .......ACA AGGGGCGGGT GGACGCACTA

LiSIDER2-32-169517r ACGCCCCGAT GACGAGGGGA GATCTCGATG CGTGGTATCA CAGATTCCAG

LiSIDER2-32-185886r ACGCCCCGAT GACGAGGGGA GATCTCGATG CGTGGTATCA CAGATTCCAG

LiSIDER2-32-420911d .......... .......... ........CG TC..GCATCT CAGAGTTCAG

LiSIDER2-32-460150r .......... ...TGGGCAA CAAATCACCG TC..GCATCT CAGAGTTCAG

LiSIDER2-32-456060d .......... ...TGGGCAA CAAATCGCCG TC..GCATCT CAGAGTTCAG

LiSIDER2-32-991979r .......GGG GTGCAAGGGG CACCTCAGCG TGTGGCATCT CAGGGTCCAG

LiSIDER2-32-1017837r .......GGG GTGCAAGGGT CACCTCAGCG TGTGGCATCT CAGGGTCCAG

LiSIDER2-32-18439r .......... .......... .ACCGCAATA CATGGTGTTA CAGGGCTCAC

LiSIDER2-32-30662r .......... .......... .ACCTCAATG CATGGCGCTA CAGGGCTCAC

LiSIDER2-32-39696d .......... .......... .ACCTCCATG CGCGGTGTCG CAGGGCCCAG

LiSIDER2-32-72349d .......... .......... .GCACCCATG CGTGATGTCG CAGGGCCCAG

LiSIDER2-32-46425r .......... .......... .ACCTCCATG CGCGGTGTCG CAGGGCCCAG

LiSIDER2-32-84516r .......... .......... .AACTCCATG CGTGGTGCCG CAGGGCCCAG

LiSIDER2-32-121058r .......... .......... .GCCTCCATG CGCGGTGTCG CAGGGCCCAG

LiSIDER2-32-127617r .......... .......... .AACTCCATG CGTGGTGCCG CAGGGCCCAG

LiSIDER2-32-1128071r .......... .......... .......... ..TGGTGT.. CAGGGTCCCG

LiSIDER2-32-222503d ACAGGGGAGG GGACGGCACA CCGCGCAGCG CGTGGTATTT CAGGGGTCTG

LiSIDER2-32-232971d ACGGGGGAGG GGACGGCACA CCGCGCAGCG CGTGGTATTT CAGGGGTCTG

LiSIDER2-32-253342d ACGAGGGAGG GGACGGCACA CCGCGCAGCG CGTGGTATTT CAGGGGTCTG

LiSIDER2-32-311078r .......GGG AAAACACACA CCGCGCAGTG CGTGGTACTT CACGCTCCAG

LiSIDER2-32-1195026d CTGTGATGAC GGGAGGGCGG CACCTCGGTG CGCGGCA..T CAAGGCCCAG

LiSIDER2-32-1219109r CTGTGATGAC GGGAGGGCGG CACCTCGGTG CGCGGCA..T CAAGGCCCAG

LiSIDER2-32-1172151d .......... .......... .........G CTCAGCA..T CAGAATCCAA

LiSIDER2-32-542863d CGGCATTGCA TGCTGTCTCA GAGCCCAGCG GCCTCCCCCC TCTCTCCCCA

LiSIDER2-32-546470r CGGCATTGCA TGCTGTCTCA GAGCCCAGCG GCCTCCCCCC TCTCTCCCCA

LiSIDER2-32-520410d CGGCATTGCA TGCTGTCTCA GAGCCCAGCG GCCTCCCCCC TCTCTCCCCA

LiSIDER2-32-588727r .......... .......... .......... .......... ..........

LiSIDER2-32-642986d .......... .......... .......... .......... ..........

LiSIDER2-32-598168r .......... .......... .......... .......... ......CTCC

LiSIDER2-32-919839r CATCAGAGTC CAGCGCCCTC CCCCCTACAC GCTCTCTCTT TCTCTGTGGG

LiSIDER2-32-953057r CATCAGAGTC CAGCGCCCTC CCCCCTACAC GCTCTCTCTT TCTCTGTGGG

LiSIDER2-32-937896d .......... .......... .......... .......... ..........

LiSIDER2-32-726312r .......... .......... .......... .......... ....AGGCTT

LiSIDER2-32-752616r .......... .......... .......... .......... ....GGGGGG

LiSIDER2-32-734536r .......... .......... .......... .......... ....GGGGGG

LiSIDER2-32-755765r .......... .......... .......... .......... ....GGGGGG

LiSIDER2-32-1284330d .......... .......... .......... .......... ..........

LiSIDER2-32-775723r .......... .......... .......... .......... ..........

LiSIDER2-32-794401r .......... .......... .....GGCCA GTCTCCATGC GTGTTATATC

LiSIDER2-32-802492r .......... .......... ....GGACAC CTCCCAGTGT GTGGTATCTC

LiSIDER2-32-827594d .......... .......... ....GAAAAC CCCCCAGTGC GTGGTATCTC

LiSIDER2-32-808837r .......... .......... ....AGACAC CTCCCAGTGC GTGGTATCTC

LiSIDER2-32-769406r .......... .......... .......... .......... ..........

LiSIDER2-32-881720r G.GGGGGG.T ACGGAGGGCA TGCCTCCGTG CTTGGTGTCT CACTGTCCAG

LiSIDER2-32-890515r G.GGGGGGGT ACGGAGGGCA TGCCTCCGTG CTTGGTGTCT CACTGTCCAG

LiSIDER2-32-1496187d A.GGCGGA.. ..TGGACACA ACCCTTCGTG TGTGGCATCT CAGGGTCCAG

LiSIDER2-32-1532945d .......... .......... .......... .......... ..........

LiSIDER2-32-1545347d GTAGCTGATG ACAGAGGACA CATCAGCGTG ......GTGG CAGGGTGCAG

LiSIDER2-32-772687r .......... .......... .......... .......... ..........

LiSIDER2-32-1141454r .......... .......... .......... .......... ..........

LiSIDER2-32-354985d ACACTGTC.. TGTGTGGAGA AGCGAAGCAG C.CA...CC. .CCCCCTCCT

LiSIDER2-32-367858d ACACTGTC.. TGTGTGGAGA AGCGAAGCAG C.CATCCCC. .CCCCCTCCT

LiSIDER2-32-661708r TCGCTCTG.. TGTATGGAGA AGCCAAGCAG CTCCTTCCCA .CTCCCCCCT

LiSIDER2-32-1405232d TCCGTCTC.. CGTGCGTGGA ATCCCAG.GG TTCAGTACC. .CTTCCCCCT

LiSIDER2-32-169517r TGC..TCC.. ACTCTCTCTG ....GTGAAG CCAGGCAGCC CCCCTCCCTC

LiSIDER2-32-185886r TGC..CCC.. ACTCTCTCTG ....GTGAAG CCAGGCAGCC CCCCTCCCTC

LiSIDER2-32-420911d TAC.CGCC.. GCTCTTTCTG ....GAAAAG CCAAGCGGC. ACCCTT.CCC

LiSIDER2-32-460150r TAC.CGCC.. GCTCTTTCTG ....GAAAAG CCAAGCGGC. ACCCTT.CCC

LiSIDER2-32-456060d TAC.CGCC.. GCTCTTTCTG ....GAAAAG CCAAGCGGC. ACCCTTTCCC

LiSIDER2-32-991979r TGCGTCCC.. ACTCCCTCTG TCTTGGGAAG CTAAGCCGCC GCCCTATCCC

LiSIDER2-32-1017837r TGCGTCCC.. ACTCCCTCTG TCTTGGGAAG CTAAGCCGCC GCCCTATCCC

LiSIDER2-32-18439r TACT.CT... A...CTCGGC GGCAAAGCCA AGCAGCCCGG A..CCCCCTC

LiSIDER2-32-30662r TACT.CT... A...CTCGGC GGCAAAGCCA AGCAGCCCGG A..CCCCCTC

LiSIDER2-32-39696d TACC.CCC.. G...CCCTCT CTCTCTCTGG AGAAGCCGGG AGGCCACCCT

LiSIDER2-32-72349d TACC.CCC.. G...CCCTCT CTCTC..TGG AGAAGCCGGG AGGCCACCCT

LiSIDER2-32-46425r TACC.CCC.. G...CCCTCT CTCTCTCTGG AGAAGCCGGG AGGCCACCCT

LiSIDER2-32-84516r TACC.CCC.. G...CCCTCT CTCTCTCTGG AGAAGCCGGG AGGCCACCCT

LiSIDER2-32-121058r TACC.CCC.. G...CCCTCT CTCTCTCTGG AGAAGCCGGG AGGCCACCCT

LiSIDER2-32-127617r TACC.CCC.. A...CCCTCT CTCTCTCTGG AGAAGCCGGG AGGCCACCCT

LiSIDER2-32-1128071r TACC.CTC.. AACTCTGTGT GGAGAAGCCA AGCAGCCCCC CCCCCCCCTA

LiSIDER2-32-222503d CACC..CC.. A...TTGTGT GGGGAGGCGA GGCAGCCT.. .....CCACT

LiSIDER2-32-232971d CACC..CC.. A...TTGTGT GGGGAGGCGA GGCAGCCC.. .....CCACT

LiSIDER2-32-253342d CACC..CC.. A...TTGTGT GGGGAGGCGA GGCAGCCC.. .....CCACT

LiSIDER2-32-311078r TATT..CC.. A...CTGTGT GGGGGGGAAG CCAAACAG.. .....CCCCT

LiSIDER2-32-1195026d CAGCACCC.. A...CTCGGT GGAGAGGCCT GGCAGTC... .....CCCTT

LiSIDER2-32-1219109r CAGCACCC.. A...CTCGGT GGAGAGGCCT GGCAGTC... .....CCCTT

LiSIDER2-32-1172151d AAGCACAC.. A...CTCGGT GGAGAGGCCT GGCAGTC... .....CCCTT

LiSIDER2-32-542863d CTCCTT.C.. TGGGGAAGCC AAG.CAGCCC ACCCGACTCT CCCCCTGCTA

LiSIDER2-32-546470r CTCCTT.C.. TGGGGAAGCC AAG.CAGCCC ACCCGACTCT CCCCCTGCTA

LiSIDER2-32-520410d CTCCTTTC.. TGGGGAAGCC AAG.CAGCCC ACCCGACTCT CCCCCTGCTA

LiSIDER2-32-588727r .....TTG.. AGTTAAAGCC AAG.CAGCCC ACCCGACTCT CCCCCTGCTA

LiSIDER2-32-642986d .......... .......... .......... .......... ..........

LiSIDER2-32-598168r ACTCCTTC.. TGGGGAAGCC AAG.CAGCCC ACCCGACTCT CCCCCTGCTA

LiSIDER2-32-919839r GAAGGGAG.. GGAGGGGGCG GGGGCAGGCA GCTCTCCCCT CCCTCCACCT

LiSIDER2-32-953057r GAAGGGAG.. GGAGGGGGCG GGGGCAGGCA GCTCTCCCCT CCCTCCACCT

LiSIDER2-32-937896d .......... .......... .......... GTTCTCCCCT CCCTCCACCT

LiSIDER2-32-726312r CCCTATTT.. ATACTCTGTA GG.GAAGCCA AAGCA.GCCC CCCGCCCCCT

LiSIDER2-32-752616r ACATTTCA.. GTGCGCCGTA TCTGAGGGTG CAGTACACCC CCCGCCCCCT

LiSIDER2-32-734536r ACATTTCA.. GTGCGCCGTA TCTGAGGGTG CAGTACACCC CCCGCCCCCT

LiSIDER2-32-755765r ACATTTCA.. GTGCGCCGTA TCTGAGGGTG CAGTACACCC CCCGCCCCCT

LiSIDER2-32-1284330d ...TATAC.. TGTGTGGGGA AGCCATGCAG CACCCCCCCC TCCCCCTACT

LiSIDER2-32-775723r .......... .......... .......... .......... ..........

LiSIDER2-32-794401r AATGCTCA.. GTGCACCCGC CACTCTGGGA GGAAGCCAGG CAGCCTCCCT

LiSIDER2-32-802492r AGGTTTCA.. CTA.GCCACA CTCCGTGTGG TGAAGCCAGG CAGCCTCCCT

LiSIDER2-32-827594d AGGTTTCA.. CTA.GCCAAA CTCCGTGCGG TGAAGCCAGG CAGCCTCCCT

LiSIDER2-32-808837r AGGTTTCA.. CTA.GCCACA CTCCGTGTGG TGAAGCCAGG CAGCCTCCCT

LiSIDER2-32-769406r .......... .......... .GAAGCCAGG CGACTCCCCG CCCCCTCCA.

LiSIDER2-32-881720r TGCACCCCCC ACTCTGTGTG TGTGTGGGTG AGGGGGAGCC AGGCAGCCCC

LiSIDER2-32-890515r TGCACCCCCC ACTCTGTGTG TGTGTGGGTG AGGGGGAGCC AGGCAGCCCC

LiSIDER2-32-1496187d TACATCCC.. .CGCACCGTG CGGTAGGATG ACTAGCAGCC CCGCTAACCC

LiSIDER2-32-1532945d .......... .......... .......... .......... ......ACCC

LiSIDER2-32-1545347d TGCATCCT.. .CACCATCTA TGCCCTTGCG GAAAGGAGCC AGACAGCCAC

LiSIDER2-32-772687r .......... .......... .......... .......... ..........

LiSIDER2-32-1141454r .......... .......... .......... .......... ..........

LiSIDER2-32-354985d CTCCCTG.CC GG.TGCA... .CGACC..AC CTCTGACCGT ...GATAGGG

LiSIDER2-32-367858d CTCCCTG.CC GG.TGCA... .CGACC..AC CTCTGACCGT ...GATAGGG

LiSIDER2-32-661708r CTCGCTG.CC GAATGCC... .GAGCG..GC GTCTGATGGT ...GACAGGG

LiSIDER2-32-1405232d ATCTATG.CC CA.TGCC... .GAACC..GC TTCTGGTGGT ...GAGAAGG

LiSIDER2-32-169517r CCCCCTTGTC CGCTGCCAGC GCACCACTAC CTCTGGTGGT ...GGCAGGG

LiSIDER2-32-185886r CCCCCTTGTC CGCTGCCAGC GCACCACTAC CTCTGGTGGT ...GGCAGGG

LiSIDER2-32-420911d CCCTCC.... ..CCGCCA.. ..TCCCTTGC CCCTGCCGA. ...GCC...A

LiSIDER2-32-460150r CCCTCC.... ..CCGCTA.. ..TCCCTTGC CCCTGCCGA. ...GCC...A

LiSIDER2-32-456060d CCCTCC.... ..CCGCCA.. ..TCCCTTGC CCCTGCCGA. ...GCC...A

LiSIDER2-32-991979r TGCCAC.... ..ATGCCG.. ..AACCCCAC TTCTGGTGAT ...GCCGGGA

LiSIDER2-32-1017837r TGCCAC.... ..ATGCCG.. ..AACCCCAC TTCTGGTGAT ...GCCGGGA

LiSIDER2-32-18439r ATTCCTTGCG AAGTGCC... .GAACC..AC CTGTGGTGGT ...GACAGGG

LiSIDER2-32-30662r ATTCCTTGCG AAGTGCC... .GAACC..AC CTGTGGTGGT ...GACAGGG

LiSIDER2-32-39696d ATTGCTGAGC AAGTGCC... .GAACC..GC CTGTGGTGGT ...GACAGGG

LiSIDER2-32-72349d ATCGCTGACC AAGTGCC... .GAACC..AC CTGTGGTGGT ...GACAGGG

LiSIDER2-32-46425r ATTGCTGAGC AAGTGCC... .GAACC..GC CTGTGGTGGT ...GACAGGG

LiSIDER2-32-84516r ATCGCTGGCC AAGTGCC... .GAACC..AC CTGTGGTGGT ...GACAGGG

LiSIDER2-32-121058r ATCGCTGACC AAGTGCC... .GAACC..AC CTGTGGTGGT ...GACAGGG

LiSIDER2-32-127617r ATCGCTGACC AAGTGCC... .GAACC..AC CTGTGGTGGT ...GACAGGG

LiSIDER2-32-1128071r ATTCCTG..C TAGCGCCA.. .GAGCC..AC CTCTCGTGGT ...GACAGGG

LiSIDER2-32-222503d ACCCCTG..C CAATGCA... .CAAGC..AC CTCTGGTGGT ...GACAGGG

LiSIDER2-32-232971d ACCCCTG..C CAATGCA... .CAAGC..AC CTCTGGTGGT ...GACAGGG

LiSIDER2-32-253342d ACCCCTG..C CAATGCA... .CAAGC..AC CTCTGGTGGT ...GACAGGG

LiSIDER2-32-311078r GTCCCTG..T CAATGCC... .GAGCC..AC TTCTGGTGGT ...GGCGGAG

LiSIDER2-32-1195026d ATACATG..C CGGCGCC... .GAGCC..AC CCTTCATGGT ...GGCAGGG

LiSIDER2-32-1219109r ATACATG..C CGGCGCC... .GAGCC..AC CCTTCATGGT ...GGCAGGG

LiSIDER2-32-1172151d ATACATG..C CGGCGCC... .GAGCC..AC CCTTCATGGT ...GGCAGGG

LiSIDER2-32-542863d TCCTCCG.CC AAATGCC... .GAGCC..AC CTCTGGCTGT ...GGCACGA

LiSIDER2-32-546470r TCCTCCG.CC AAATGCC... .GAGCC..AC CTCTGGCTGT ...GGCACGA

LiSIDER2-32-520410d TCCTCCG.CC AAATGCC... .GAGCC..AC CTCTGGCTGT T..GGCACGA

LiSIDER2-32-588727r TCCTCCG.CC AAATGCC... .GAGCC..AC CTCTGGCTGT ...GGCACGA

LiSIDER2-32-642986d TCCTGGG.CC AAATGCC... .GAGCC..AC CTCTGGCTGT ...GGCACGA

LiSIDER2-32-598168r TCCTCCG.CC AAATGCC... .GAGCC..AC CTCTGGCTGT ...GGCACGA

LiSIDER2-32-919839r ATCCCTG.CC GAATGCC... .GAACCTCAC TTCCGGGGAT G..CCGGGGG

LiSIDER2-32-953057r ATCCCTG.CC GAATGCC... .GAACCTCAC TTCCGGGGAT G..CCGGGGG

LiSIDER2-32-937896d ATCCCTG.CC GAATGCC... .GAACCTCAC TTCCGGGGAT G..CCGGGGG

LiSIDER2-32-726312r AGCCCTG.CC AAATGCC... .GGGCC..AC CTCTGGTGGT ...GACAGGG

LiSIDER2-32-752616r AGCCCTG.CC AAATGCC... .GGGCC..AC CTCTGGTGGT ...GACAGGG

LiSIDER2-32-734536r AGCCCTG.CC AAATGCC... .GGGCC..AC CTCTGGTGGT ...GACAGGG

LiSIDER2-32-755765r AGCCCTG.CC AAATGCC... .GGGCC..AC CTCTGGTGGT ...GACAGGG

LiSIDER2-32-1284330d ATCCCTG.CC .AGTGCT... .GAACT..GC CTCTGGTGGT GGTGACAGGG

LiSIDER2-32-775723r .......... .......... .......... .......... ..........

LiSIDER2-32-794401r ATCCCCT.TC CGATGCA... .CAGCC..AC CTCTGGTGGT ...GACAGGG

LiSIDER2-32-802492r ATCCCCT.TC CGATGCA... .CAGCC..AC CTCTGGTGGT ...GACAGGG

LiSIDER2-32-827594d ATCCCCT.TC CGATGCA... .CAGCC..AC CTCTGGTGGT ...GACAGGG

LiSIDER2-32-808837r ATCCCCT.TC CGATGCA... .CAGCC..AC CTCTGGTGGT ...GACAGGG

LiSIDER2-32-769406r ATTTCTA.TG AAATGTC... .AAACT..GC TTTTGGCCAT ...GACAGGG

LiSIDER2-32-881720r CTATCGCTGC CAATGCT... .GCGCC..AC CTCTGGTGGT G..ACC.GGG

LiSIDER2-32-890515r CTATCGCTGC CAATGCT... .GCGCC..AC CTCTGGTGGT G..ACC.GGG

LiSIDER2-32-1496187d TTGCCA.... .AATGCC... .GGCCC..AC CTCTGGTGGT G..GCAAGGG

LiSIDER2-32-1532945d TTGCCA.... .AATGCC... .GGCCC..AC CTCTGGTGGT G..GCAAGGG

LiSIDER2-32-1545347d CCACCTGC.C AACTGCG... .AAACC..AC TGCTCGCCGG G..GCG.AAG

LiSIDER2-32-772687r .......... .......... .......... .......... ..........

LiSIDER2-32-1141454r .......... .......... .......... .......... ..........

LiSIDER2-32-354985d TCAA...... ..GCGCCCAC GACGTAGGG. .......... ..........

LiSIDER2-32-367858d TCAA...... ..GCGCCCAC GACGTAGGG. .......... ..........

LiSIDER2-32-661708r TCCA...... ..GCACCCAC GACCTGGGG. .......... ..........

LiSIDER2-32-1405232d CCAA...... ..GCACCTAC GAGGTAGGG. .......... ..........

LiSIDER2-32-169517r TCAA...... ..GCACCTAC GACATGGGG. .......... ..........

LiSIDER2-32-185886r TCAA...... ..GCACCTAC GACATGGGG. .......... ..........

LiSIDER2-32-420911d CCTG...... ..TGATTAGG AG...AGGG. .......... ..........

LiSIDER2-32-460150r CCTG...... ..TGATTAGG AG...AGGG. .......... ..........

LiSIDER2-32-456060d CCTG...... ..TGATTAGG AG...AGGG. .......... ..........

LiSIDER2-32-991979r TCAC...... ..GCATCCAC GG...GGCA. .......... ..........

LiSIDER2-32-1017837r TCAC...... ..GCATCCAC GG...GGCA. .......... ..........

LiSIDER2-32-18439r TCGC...... CGGCGCCGGC GCCGTGCTG. .......... ..........

LiSIDER2-32-30662r TCGC...... CGGCGCCGGC GCCGTGCTG. .......... ..........

LiSIDER2-32-39696d TCGCAGGCGC CGGCGCCGGC GCCGTGCTG. .......... ..........

LiSIDER2-32-72349d TCGCAGGCGC CGGCGCCGGC GCCGTGCTG. .......... ..........

LiSIDER2-32-46425r TCGCAGGCGC CGGCGCCGGC GCCGTGCTG. .......... ..........

LiSIDER2-32-84516r TCGC...... CGGCGCCGGC GCCGTGCTG. .......... ..........

LiSIDER2-32-121058r TCGCA..... .GGCGCCGGC GCCGTGCTG. .......... ..........

LiSIDER2-32-127617r TCGCA..... .GGCGCCGGC GCCGTGCTG. .......... ..........

LiSIDER2-32-1128071r TCAGG..... TGCCTACGAC GTAGAGGGG. .......... ..........

LiSIDER2-32-222503d CCA....... .GGTGCCTGC GACG.AAGG. .......... ..........

LiSIDER2-32-232971d TCA....... .GGTGCCTGC GACG.AAGG. .......... ..........

LiSIDER2-32-253342d TCA....... .GGTGCCTGC GACG.AAGG. .......... ..........

LiSIDER2-32-311078r TCA....... .TGCACCTAC GACTTAGGG. .......... ..........

LiSIDER2-32-1195026d TCA....... .GGCGCCTAC GACG.TAGG. .......... ..........

LiSIDER2-32-1219109r TCA....... .GGCGCCTAC GACG.TAGG. .......... ..........

LiSIDER2-32-1172151d TCA....... .GGCGCCTAC GACG.TAGG. .......... ..........

LiSIDER2-32-542863d TCAG...... .G.CACCTAC GACGTAAGGC AGCGGGGGTG GAGGAGGAGG

LiSIDER2-32-546470r TCAG...... .G.CACCTAC GACGTAAGGC AGCGGGGGTG GAGGAGGAGG

LiSIDER2-32-520410d TCAG...... .G.CACCTAC GACGTAAGGC AGCGGGGGTG GAGGAGGAGG

LiSIDER2-32-588727r TCAG...... .G.CACCTAC GACGTAAGGC AGCGGGGGTG GAGGAGGAGG

LiSIDER2-32-642986d TCAG...... .G.CACCTAC GACGTAAGGC AGCGGGGGTG GAGGAGGAGG

LiSIDER2-32-598168r TCAG...... .G.CACCTAC GACGTAAGGC AGCGGGGGTG GAGGAGGAGG

LiSIDER2-32-919839r CCCG...... .G.CACCCAC GACGTAAGG. .......... .....GGAGG

LiSIDER2-32-953057r CCCG...... .G.CACCCAC GACGTAAGG. .......... .....GGAGG

LiSIDER2-32-937896d CCCG...... .G.CACCCAC GACGTAAGG. .......... .....GGAGG

LiSIDER2-32-726312r CCGG...... .G.TGCCTAC GACGTAGGG. .......... ..........

LiSIDER2-32-752616r CCGG...... .G.TGCCTAC GACGTAGGG. .......... ..........

LiSIDER2-32-734536r CCGG...... .G.TGCCTAC GACGTAGGG. .......... ..........

LiSIDER2-32-755765r CCGG...... .G.TGCCTAC GACGTAGGG. .......... ..........

LiSIDER2-32-1284330d CCGG...... .GGTGCCTAC GACTTGGGA. .......... ..........

LiSIDER2-32-775723r .......... .......... .......... .......... ..........

LiSIDER2-32-794401r CTGA...... .G.TGCCTAC GGCGTAGGG. .......... ..........

LiSIDER2-32-802492r CTGA...... .G.TGCCTAC GGCGTAGGG. .......... ..........

LiSIDER2-32-827594d TTGA...... .G.TGCCTAC GGCGTAGGG. .......... ..........

LiSIDER2-32-808837r CTGA...... .G.TGCCTAC GGCGTAGGG. .......... ..........

LiSIDER2-32-769406r TCAA...... .G.AACCTGA AGCAT..GG. .......... ..........

LiSIDER2-32-881720r TGAG...... ..GCGCCTGC GGC.GTAGG. .......... ..........

LiSIDER2-32-890515r TGAG...... ..GCGCCTGC GGC.GTAGG. .......... ..........

LiSIDER2-32-1496187d TTAA...... ..GCGCGTAC GACAGAAGG. .......... ..........

LiSIDER2-32-1532945d TTAA...... ..GCGCGTAC GACAGAAGG. .......... ..........

LiSIDER2-32-1545347d CCAA...... ..GTACCTTC CAC.GTACG. .......... ..........

LiSIDER2-32-772687r .......... .......... .......... .......... ..........

LiSIDER2-32-1141454r .......... .......... .......... .......... ..........

LiSIDER2-32-354985d ..GAGGGGGG AGAGGAGAGC AGAGTGATTC ACCGCTA..C GGATGTT...

LiSIDER2-32-367858d ..GAGGGGGG AGAGGAGAGC AGAGTGATTC ACCGCTA..C GGATGTT...

LiSIDER2-32-661708r ..GAAGGGA. ....GGGGGT G.......TC AGAGCGA..T GCA..CC...

LiSIDER2-32-1405232d ..AGGAGGTC AGCTGGATGT A.......TC GCTACTG..C GAATGCA...

LiSIDER2-32-169517r .AGGGC.... .......GCC AGTGCGATTA ATCGCTG..C TGATGTC...

LiSIDER2-32-185886r .AGGGC.... .......GCC AGTGCGATTA ATCGCTG..C TGATGTC...

LiSIDER2-32-420911d .CGAAG.... .......CCA GGGCCGCTTT ACGGCTAG.C CGGTGTC...

LiSIDER2-32-460150r .CGAAG.... .......CCA GGGCCGCTTT ACGGCTAG.C CGGTGTC...

LiSIDER2-32-456060d .CGAAG.... .......CCA GGGCCGCTTT ACGGCTAG.C CGGTGTC...

LiSIDER2-32-991979r .AGGAG.... .......CTG AGAGCAACGC ACTCCTA..C GAACGTC...

LiSIDER2-32-1017837r .AGGAG.... .......CTG AGAGCAACGC ACTCCTA..C GAACGTC...

LiSIDER2-32-18439r ..GAAG.... ........TC CGGGCGACGT ATCGCTG..C TGATGTA...

LiSIDER2-32-30662r ..GAAG.... ........TC CGGGCGACGT ATCGCTG..C TGATGTA...

LiSIDER2-32-39696d ..GAGG.... ........TC CGGGCGACGT ATCGCTG..C TGATGTA...

LiSIDER2-32-72349d ..GAGG.... ........TC CGGGCGACGT ATCGCTG..C TGATGTA...

LiSIDER2-32-46425r ..GAAG.... ........TC CGGGCGACGT ATCGCTG..C TGATGTA...

LiSIDER2-32-84516r ..GAGG.... ........TC CGAGCGACGT ATCGCTG..C TGATGTA...

LiSIDER2-32-121058r ..GAGG.... ........TC CAGGCGACGT ATCGCTG..C TGATGTA...

LiSIDER2-32-127617r ..GAGG.... ........TC CAGGCGACGT ATCGCTG..C TGATGTA...

LiSIDER2-32-1128071r ..AATG.... ........TC AGAGCCATGC ATCACTG..C TGATGTGCC.

LiSIDER2-32-222503d ..GAGG.... ........TC AGAGCGATGC ATGGCTG..C TGATGCC...

LiSIDER2-32-232971d ..GAGG.... ........TC AGAGCGATGC ATGGCTG..C TGATGCC...

LiSIDER2-32-253342d ..GAGG.... ........TC AGAGCGATGC ATGGCTG..C TGATGCC...

LiSIDER2-32-311078r ..GAGG.... ........TC AGAGCGATGG ATTGCTG..C TAGTGTC...

LiSIDER2-32-1195026d ..GGGG.... ........CC AGAGCGACTT ATCGCTA..A TGATGTC...

LiSIDER2-32-1219109r ..GGGG.... ........CC AGAGCGACTT ATCGCTA..A TGATGTC...

LiSIDER2-32-1172151d ..GGGG.... ........CC AGAGCGACTT ATCGCTA..A TGATGTC...

LiSIDER2-32-542863d AGGAGGAGGA GG...GTGTC TGAGCGGTGC ATCGCTG..C TGACGTC...

LiSIDER2-32-546470r AGGAGGAGGA GGAGGGGGTC TGAGCGGTGC ATCGCTG..C TGACGTC...

LiSIDER2-32-520410d AGGAGGGG.. .......GTC TGAGCGGTGC ATCGCTG..C TGACGTC...

LiSIDER2-32-588727r AGGAGGAGG. .....GTGTC TGAGCGGTGC ATCGCTG..C TGACGTC...

LiSIDER2-32-642986d AGGAGGAGGA GGAGGGGGTC TGAGCGGTGC ATCGCTG..C TGACGTC...

LiSIDER2-32-598168r AGGAGG.... .....GTGTC TGAGCGGTGC ATCGCTG..C TGACGTC...

LiSIDER2-32-919839r AGGGAG.... .......GCC CGGGCGATGC ATCGCTA..C AGATGCC...

LiSIDER2-32-953057r AGGGAG.... .......GCC CGGGCGATGC ATCGCTA..C AGATGCC...

LiSIDER2-32-937896d AGGGAG.... .......GCC CGGGCGATGC ATCGCTA..C AGATGCC...

LiSIDER2-32-726312r ..GAAG.... .......GCC CGAGCGATGC ATCACTG..C TGATGTC...

LiSIDER2-32-752616r ..GAAG.... .......GCC CGAGCGATGC ATCACTG..C TGATGTC...

LiSIDER2-32-734536r ..GAAG.... .......GCC CGAGCGATGC ATCACTG..C TGATGTC...

LiSIDER2-32-755765r ..GAAG.... .......GCC CGAGCGATGC ATCACTG..C TGATGTC...

LiSIDER2-32-1284330d ...AAG.... ........CC AGAGCGATTT ATCGCCA..C GGAGGCC...

LiSIDER2-32-775723r .......... .......... .......... .......... ..........

LiSIDER2-32-794401r ..GAAG.... .......GCC AGAGCGATGC ATCGCGA..C TGATGTG...

LiSIDER2-32-802492r ..GAAG.... .......GCC AGAGCGATGC ATCGCGA..C TGATGTG...

LiSIDER2-32-827594d ..GAAG.... .......GCC AGAGCGATGC ATCGCGA..C TGATGTG...

LiSIDER2-32-808837r ..GAAG.... .......GCC AGAGCGATGC ATCGCGA..C TGATGTG...

LiSIDER2-32-769406r ..GAAG.... .......GCC AGAGTGATGC ATCGCTG..C TGATGTC...

LiSIDER2-32-881720r ..GGGT.... ........GC GAG.CGATGC ATCGCTG..C TGATGCC...

LiSIDER2-32-890515r ..GGGT.... ........GC GAG.CGATGC ATCGCTG..C TGATGCC...

LiSIDER2-32-1496187d ..GAGT.... ........CC GGGGCGATGC ATCGCTG..C CGATGCC...

LiSIDER2-32-1532945d ..GAGT.... ........CC GGGGCGATGC ATCGCTG..C CGATGCC...

LiSIDER2-32-1545347d ..GAGG.... ........TC AGAGCGACCC A.CGCTGT.C TGATGTC...

LiSIDER2-32-772687r .......... .......... ........AT GTTTCTAC.T TCTTTGCGT.

LiSIDER2-32-1141454r .......... .........T GGAGGAGCGC GGTGATGCCT TGATGGCGGA

LiSIDER2-32-354985d GGCGGTCAGG T.CCTGGATG G.CGT..TGT GTCGGTG.TG ACCTGCGACG

LiSIDER2-32-367858d GGCGGTCAGG T.CCTGGATG G.CGT..TGT GTCGGTG.TG ACCTGCGACG

LiSIDER2-32-661708r GCTGCTGATG C.C...GGCG GTC.C..TGC CTTGTAT.GG CGGTGCAA..

LiSIDER2-32-1405232d GGCGGTGAGG T.CCTTGACG GGCGT..TGC GTCGGGA.AG ACGTGCAACA

LiSIDER2-32-169517r GGCGGCCGGG C.CCTGGATG G.CGT..TGC TTTAGGG.TG ACCGGTGAC.

LiSIDER2-32-185886r GGCGGCCGGG C.CCTGGATG G.CGT..TGC TTTAGGG.TG ACCGGTGAC.

LiSIDER2-32-420911d GGCGGCCAGG T.CCTGGGCG G.CGC..TGC GTCGGAG.CG GCCCGAGACG

LiSIDER2-32-460150r GGCGGCCAGG T.CCTGGGCG G.CGC..TGC GTCGGAG.CG GCCCGAGACG

LiSIDER2-32-456060d GGCGGCCAGG T.CCTGGGCG G.CGC..TGC GTCGGAG.CG GCCCGAGACG

LiSIDER2-32-991979r GGGGGCCAGG T.TCTGGAGG G.CGT..CTC GCCAGGG.TG ACCAGCGAC.

LiSIDER2-32-1017837r GGGGGCCAGG T.TCTGGAGG G.CGT..CTC GCCAGGG.TG ACCAGCGAC.

LiSIDER2-32-18439r GTTGGTCGGG T.CCTGGCCG GGCGTTGCGT ..CGGGG.CG ACCGGCGACA

LiSIDER2-32-30662r GTTGGTCGGG T.CCTGGCCG GGCGTTGCGT ..CGGGG.CG ACCGGCGACA

LiSIDER2-32-39696d GTTGGCCGGG T.CCTGGCTG GGCGTTGCGT ..CGGGG.CG ACCGGCGACA

LiSIDER2-32-72349d GTTGGCCGGG T.CCTGGCTG GGCGTTGCGT ..CGGGG.CG ACCGGCGACA

LiSIDER2-32-46425r GTTGGTCGGG T.CCTGGCCG GGCGTTGCGT ..CGGGG.CG ACCGGCGACA

LiSIDER2-32-84516r GTTGGTCGGG T.CCTGGCTG GGCGTTGCGT ..CGGGG.CG ACCGGCGACA

LiSIDER2-32-121058r GTTGGCCGGG T.CCTGGCTG GGCGTTGCGT ..CGGGG.CG ACCGGCGACA

LiSIDER2-32-127617r GTTGGCCGGG T.CCTGGCTG GGCGTTGCGT ..CGGGG.CG ACCGGCGACA

LiSIDER2-32-1128071r GGCGGTGGGG T.CCTGGATG G.CGTTGCGT ..CGGAG.CC GCCTGCGACA

LiSIDER2-32-222503d GGCGCTCAGG C.CCTGGATG G.CGTTGCGT ..GGGGG.CG ACCTGCGACG

LiSIDER2-32-232971d GGCGCTCAGG C.CCTGGATG G.CGTTGCGT ..GGGGG.CG ACCTGCGACG

LiSIDER2-32-253342d GGCGCTCAGG C.CCTGGATG G.CGTTGCGT ..GGGGG.CG ACCTGCGACG

LiSIDER2-32-311078r GGCGGTGAGG T.CCTGGATA G.CGCTGCGT ..CGGAG.TG ACCTGCGGTG

LiSIDER2-32-1195026d GGCGGTGAGG T.GCTGGATG G.CGCTGCGT TGGGGAG.AG ATCCGCCACT

LiSIDER2-32-1219109r GGCGGTGAGG T.GCTGGATG G.CGCTGCGT TGGGGAG.AG ATCCGCCACT

LiSIDER2-32-1172151d GGCGGTGAGG T.GCTGGATG G.CGCTGCGT TGGGGAG.AG ATCCGCCACT

LiSIDER2-32-542863d GGCGGTCCGG T.GCTGGACG G.CGT..GGC GTCGCAG.CG GCCCGCGATC

LiSIDER2-32-546470r GGCGGTCCGG T.GCTGGACG G.CGT..GGC GTCGCAG.CG GCCCGCGATC

LiSIDER2-32-520410d GGCGGTCCGG T.GCTGGACG G.CGT..GGC GTCGCAG.CG GCCCGCGATC

LiSIDER2-32-588727r GGCGGTCCGG T.GCTGGACG G.CGT..GGC GTCGCAG.CG GCCCGCGATC

LiSIDER2-32-642986d GGCGGTCCGG T.GCTGGACG G.CGT..GGC GTCGCAG.CG GCCCGCGATC

LiSIDER2-32-598168r GGCGGTCCGG T.GCTGGACG G.CGT..GGC GTCGCAG.CG GCCCGCGATC

LiSIDER2-32-919839r GGCGCCCAGG T.CCTGGATG G.CGC..TGC GTCGGGG.CA CCCCCCCTGC

LiSIDER2-32-953057r GGCGCCCAGG T.CCTGGATG G.CGC..TGC GTCGGGG.CA CCCCCCCTGC

LiSIDER2-32-937896d GGCGCCCAGG T.CCTGGATG G.CGC..TGC GTCGGGG.CA CCCCCCCTGC

LiSIDER2-32-726312r GGCGGTGAGG G.GCTGGATG G.CGC..TGC GTCGGAC.CG ACCTGCGACC

LiSIDER2-32-752616r GGCGGTGAGG G.GCTGGATG G.CGC..TGC GTCGGAC.CG ACCTGCGACC

LiSIDER2-32-734536r GGCGGTGAGG G.GCTGGATG G.CGC..TGC GTCGGAC.CG ACCTGCGACC

LiSIDER2-32-755765r GGCGGTGAGG G.GCTGGATG G.CGC..TGC GTCGGAC.CG ACCTGCGACC

LiSIDER2-32-1284330d GGCGGCCACG T.CCAGGACG G.CGCGCTTT GTCGGGG.CG ACCCGCCACC

LiSIDER2-32-775723r .......... .......... .......... .......... ..........

LiSIDER2-32-794401r GACGGCCAGG C.TCTGCACG G.CGC..CGC GTCAGAG.CG CTCTGCGACG

LiSIDER2-32-802492r GACGGCCAGG C.TCTGCACG G.CGC..CGC GTCAGAG.CG CTCTGCGACG

LiSIDER2-32-827594d GACGGCCAGG C.TCTGCACG G.CGC..CGC GTCAGAG.CG CTCTGCGACG

LiSIDER2-32-808837r GACGGCCAGG C.TCTGCACG G.CGC..CGC GTCAGAG.CG CTCTGCGACG

LiSIDER2-32-769406r GGCGGCTAGG T.CGCGGATG G.CGC..TGC GTTGGAG.GG CGGTGCGATG

LiSIDER2-32-881720r GGCGGTGATG C.TGTGGATG G.CGC..TGC GTCGGGG.CG GCCCGCGG.C

LiSIDER2-32-890515r GGCGGTGATG C.TGTGGATG G.CGC..TGC GTCGGGG.CG GCCCGCGG.C

LiSIDER2-32-1496187d CGCGGCCAGG T.CCTGGATG G.CAC..GTC GCCGAAG.AG ACCTGCGTTC

LiSIDER2-32-1532945d CGCGGCCAGG T.CCTGGATG G.CAC..GTC GCCGAAG.AG ACCTGCGTTC

LiSIDER2-32-1545347d GGCGGTCAGG G.CCTCGATG G.CAC..GGC ATCGGAGGCG ACCCGCTA.T

LiSIDER2-32-772687r GCGCTCCTGA T.GACAGGGG GACATC.TCC GCCTGGC... ATCCGCGGCC

LiSIDER2-32-1141454r GGCGGAGAAA CAGCTGAAGA AGCGCTCGTG GTTCTCGTCG AGCGAC.ACG

LiSIDER2-32-354985d TT....GAAC ATGTTTGCAC C..ACCTGCA TGA...TGGG CAAAGCGCTA

LiSIDER2-32-367858d TT....GAAC ATGTTTGCAC C..ACCTGCA TGA...TGGG CAAAGCGCTA

LiSIDER2-32-661708r ........AC T..TTTGCAC C..ATCTATG TGA...TGGG CGACCTGCCA

LiSIDER2-32-1405232d GC....GAAC CGGCTTGTGC G..ACCCACA TGA...TGGG CGAAGTGCCA

LiSIDER2-32-169517r .A..CCGAGC ATATCTGCGC C..ATCCATA TAA...TGCG CAAAGTGCCA

LiSIDER2-32-185886r .A..CCGAGC ATATCTGCGC C..ATCCATA TAA...TGCG CAAAGTGCCA

LiSIDER2-32-420911d AT..GCGAGC ACGCTCGTGC C..ACCTATA TGG...TGGG CGAAGCGCCA

LiSIDER2-32-460150r AT..GCGAGC ACGCTCGTGC C..ACCTATA TGG...TGGG CGAAGCGCCA

LiSIDER2-32-456060d GT..GCGAGC ACGCTCGTGC C..ACCTATA TGG...TGGG CGAAGCGCCA

LiSIDER2-32-991979r .C..GCAAGC ACGCTTGTGC C..ATCCGTA TAA...TGGG GAAAGGGCCC

LiSIDER2-32-1017837r .C..GCAAGC ACGCTTGTGC C..ATCCGTA TAA...TGGG GAAAGGGCCC

LiSIDER2-32-18439r GT....GAAC ACGCGTGTGC C..ATCCATA TGG.TTCG.G CAGCGTGTCA

LiSIDER2-32-30662r GT....GAAC ACGCGTGTGC C..ATCCATA TGG.TTCG.G CAGCGTGTCA

LiSIDER2-32-39696d GT....GAAC ACGCGTGTGC C..ATCCACA TGA.TTCG.G CAGCGTGTCA

LiSIDER2-32-72349d GT....GAAC ACGCGTGTGC C..ATCCACA TGA.TTCG.G CAGCGTGTCA

LiSIDER2-32-46425r GT....GAAC ACGCGTGTGC C..ATCCACA TGA.TTCG.G CAGCGTGTCA

LiSIDER2-32-84516r GT....GAAC ACGCGTGTGC C..ATCCACA TGA.TTCG.G CAGCGTGTCA

LiSIDER2-32-121058r GT....GAAC ACGCGTGTGC C..ATCCATA TGA.TTCG.G CAGCGTGTCA

LiSIDER2-32-127617r GT....GAAC ACGCGTGTGC C..ATCCATA TGA.TTCG.G CAGCGTGTCA

LiSIDER2-32-1128071r GT....GAAC ACATCTGTGC C..ATCCATG TGA.TA.G.G CAGCGTGTCC

LiSIDER2-32-222503d GT....GAAC GCGTCTGTGC A..GCCCATA TGG.CCAG.G CAGCGTGTGA

LiSIDER2-32-232971d GT....GAAC GCGTCTGTGC A..GCCCATA TGG.CCAG.G CAGCGTGTGA

LiSIDER2-32-253342d GT....GAAC GCGTCTGTGC A..GCCCATA TGG.CCAG.G CAGCGTGTGA

LiSIDER2-32-311078r TT....GAAC ATGTTTGTGC C..GTCGACA TGA.TGGGCA GAGCGTCCCA

LiSIDER2-32-1195026d GT....GCGC ACGCCTGTGC C..GTCCGTA TGC.TGGG.. CGGAGTGTCA

LiSIDER2-32-1219109r GT....GCGC ACGCCTGTGC C..GTCCGTA TGC.TGGG.. CGGAGTGTCA

LiSIDER2-32-1172151d GT....GCGC ACGCCTGTGC C..GTCCGTA TGC.TGGG.. CGGAGTGTCA

LiSIDER2-32-542863d GT..GTCAGC AGGGCGGCGC C..ATCCATA GGAGTTTAGG CGGCGTGCCA

LiSIDER2-32-546470r GT..GTCAGC AGGGCGGCGC C..ATCCATA GGAGTTTAGG CGGCGTGCCA

LiSIDER2-32-520410d GT..GTCAGC AGGGCGGCGC C..ATCCATA GGAGTTTAGG CGGCGTGCCA

LiSIDER2-32-588727r GT..GTCAGC AGGGCGGCGC C..ATCCATA GGAGTTTAGG CGGCGTGCCA

LiSIDER2-32-642986d GT..GTCAGC AGGGCGGCGC C..ATCCATA GGAGTTTAGG CGGCGTGCCA

LiSIDER2-32-598168r GT..GTCAGC AGGGCGGCGC C..ATCCATA GGAGTTTAGG CGGCGTGCCA

LiSIDER2-32-919839r GACAGCGAGC ACGCTTGTGC CTAATCCATA TGA..TTAGG CAGAGCGTCA

LiSIDER2-32-953057r GACAGCGAGC ACGCTTGTGC CTAATCCATA TGA..TTAGG CAGAGCGTCA

LiSIDER2-32-937896d GACAGCGAGC ACGCTTGTGC CTAATCCATA TGA..TTAGG CAGAGCGTCA

LiSIDER2-32-726312r GT....GCAC ACGCTTGCGC C..ATCCACG TGA...TGGG CAGAGCGTCC

LiSIDER2-32-752616r GT....GCAC ACGCTTGCGC C..ATCCACG TGA...TGGG CAGAGCGTCC

LiSIDER2-32-734536r GT....GCAC ACGCTTGCGC C..ATCCACG TGA...TGGG CAGAGCGTCC

LiSIDER2-32-755765r GT....GCAC ACGCTTGCGC C..ATCCACG TGA...TGGG CAGAGCGTCC

LiSIDER2-32-1284330d GT....GGAC ATGCTTGTAC CACACGCACG CATG.ACGGG CAAAGTGCCA

LiSIDER2-32-775723r .......... .......... .......... .....TTAGG CAGAGTGTAC

LiSIDER2-32-794401r GT....GCGC CCGCTTGCAC T.AACCCATA TGA..TTAGG CAGAGTGTCA

LiSIDER2-32-802492r GT....GCGC ACGCTTGCAC T.AACCCATA TGA..TTAGG CAGAGTGTCA

LiSIDER2-32-827594d GT....GCGC ACGCTTGCAC T.AACCCATA TGA..TTAGG CAGAGTGTCA

LiSIDER2-32-808837r GT....GCGC ACGCTTGCAC T.AACCCATA TGA..TTAGG CAGAGTGTCA

LiSIDER2-32-769406r CT....AAAC ACGCTTGTGC C.G.TCTATA TGA..C.GGG CAGAGCGTCA

LiSIDER2-32-881720r AG...TGAGC ACGCCTGTAC C..ACCCATA TGG..TAGGC AAGAGTGCCT

LiSIDER2-32-890515r AG...TGAGC ACGCCTGTAC C..ACCCATA TGG..TAGGC AAGAGTGCCT

LiSIDER2-32-1496187d AG...CGAAC GCGCCTGCGC T..ATCCATG TGG..CTGGG CAGGGCGTCG

LiSIDER2-32-1532945d AG...CGAAC GCGCCTGCGC T..ATCCATG TGG..CTGGG CAGGGCGTCG

LiSIDER2-32-1545347d TG...TGAAT CGGCTTGCGC C..ATTCACA TCA..C.AGC GAGTGTGTCA

LiSIDER2-32-772687r TA..GCGCCC CTCCCTCTCC GCACTCTATG TGG......G AGGGGTGGAG

LiSIDER2-32-1141454r AAAGTTGATG AGGCGCACGA CACGTTCCTG CAGGCTGCCA CGCAGTACAA

LiSIDER2-32-354985d GCGTGGCTCG AGC.GTA... .......... ...CCTCACA CCCGGCCCCT

LiSIDER2-32-367858d GCGTGGCTCG AGC.GTA... .......... ...CCTCACA CCCGGCCCCT

LiSIDER2-32-661708r GCGTGACTCG AGC.GTAACC .......... ...CCCCCCC CCCG.GCCCT

LiSIDER2-32-1405232d CCGTGACTCG AGC.GCA... .......... .....TCGCG CCCG.CCCCT

LiSIDER2-32-169517r GCGTGGCTGG AAC.GCACC. .......... .....TCACG CG.....CAC

LiSIDER2-32-185886r GCGTGGCTGG AAC.GCACC. .......... .....TCACG CG.....CAC

LiSIDER2-32-420911d ACGTGCCTCG AGC.GCATC. .......... .....CCGCC CCACGGCCCT

LiSIDER2-32-460150r ACGTGCCTCG AGC.GCATC. .......... .....CCGCC CCACGGCCCT

LiSIDER2-32-456060d ACGTGCCTCG AGC.GCATC. .......... .....CCGCC CCACGGCCCT

LiSIDER2-32-991979r GCCTAACTGG GAG.GTATC. .......... .....TCACT CC.CGACCCT

LiSIDER2-32-1017837r GCCTAACTGG GAG.GTATC. .......... .....TCACT CC.CGACCCT

LiSIDER2-32-18439r CCGCGTCTCG AGA.GTAAC. .......... .....CCGCC ..CGGGCCCC

LiSIDER2-32-30662r CCGCGTCTCG AGA.GTAAC. .......... .....CCGCC ..CGGGCCCC

LiSIDER2-32-39696d CCGCGTCTCG AGA.GTAAC. .......... .....CCGCC ..CGGGCCCC

LiSIDER2-32-72349d CCGCGTCTCG AGA.GTAAC. .......... .....CCGCC ..CGGGCCCC

LiSIDER2-32-46425r CCGCGTCTCG AGA.GTAAC. .......... .....CCGCC ..CGGGCCCC

LiSIDER2-32-84516r CCGCGTCTCG AGA.GTAAC. .......... .....CCGCC ..CGGGCCCC

LiSIDER2-32-121058r CCGCGTCTCG AGA.GTAAC. .......... .....CCGCC ..CGGGCCCC

LiSIDER2-32-127617r CCGCGTCTCG AGA.GTAAC. .......... .....CCGCC ..CGGGCCCC

LiSIDER2-32-1128071r GCGCGACTGG AAATGCATC. .......... .....TCACC T.CCGGCCCT

LiSIDER2-32-222503d GCGTGACTGG AGT.GCTGC. .......... .....ACCTC ATGCGCCCCT

LiSIDER2-32-232971d GCGTGACTGG AGT.GCTGC. .......... .....ACCTC ATGCGCCCCT

LiSIDER2-32-253342d GCGTGACTGG AGT.GCTGC. .......... .....ACCTC ATGCGCCCCT

LiSIDER2-32-311078r GCGCGACTCG AGC.GTATC. .......... .....TCAAA GCCCGGTCCT

LiSIDER2-32-1195026d GCGTGACTCG AAC.GTGTC. .......... .....CC... ACCCGGCCCT

LiSIDER2-32-1219109r GCGTGACTCG AAC.GTGTC. .......... .....CC... ACCCGGCCCT

LiSIDER2-32-1172151d GCGTGACTCG AAC.GTGTC. .......... .....CC... ACCCGGCCCT

LiSIDER2-32-542863d GCGTGGCTCG GAC.GTATCC CCTCCCCCCG CCACCCCTCC CCTCGGCCCT

LiSIDER2-32-546470r GCGTGGCTCG GAC.GTATCC CCTCCCCCCG CCACCCCTCC CCTCGGCCCT

LiSIDER2-32-520410d GCGTGGCTCG GAC.GTATCC CCTCCCCCCG CCACCCCTCC CCTCGGCCCT

LiSIDER2-32-588727r GCGTGGCTCG GAC.GTATCC CCTCCCCCCG CCACCCCTCC CCTCGGTCCT

LiSIDER2-32-642986d GCGTGGCTCG GAC.GTATCC CCTCCCCC.G CCACCCCTCC CCTCGGTCCT

LiSIDER2-32-598168r GCGTGGCTCG GAC.GTATCC CCTCCCCCCG CCACCCCTCC CCTCGGTCCT

LiSIDER2-32-919839r GCGTGGCTCG GGC.GTGTGT CCC......A CCACCCACCC CCCCGGCCCT

LiSIDER2-32-953057r GCGTGGCTCG GGC.GTGTGT CCC......A CCACCCACCC CCCCGGCCCT

LiSIDER2-32-937896d GCGTGGCTCG GGC.GTGTGT CCC......A CCACCCACCC CCCCGGCCCT

LiSIDER2-32-726312r GCGTGACTCG AGC.GTGTA. .......... ....CCGCAC CCCCGGCCCT

LiSIDER2-32-752616r GCGTGACTCG AGC.GTGTA. .......... ....CCGCAC CCCCGGCCCT

LiSIDER2-32-734536r GCGTGACTCG AGC.GTGTA. .......... ....CCGCAC CCCCGGCCCT

LiSIDER2-32-755765r GCGTGACTCG AGC.GTGTA. .......... ....CCGCAC CCCCGGCCCT

LiSIDER2-32-1284330d GCGTGACTCG AGC.GTAT.. .......... .......CCC ACCCGATACT

LiSIDER2-32-775723r GCGTGGCTAG AGC.GCACC. .......... ......CCTT ..CCGGCCCT

LiSIDER2-32-794401r TCGCGGCTCC AAC.GCATC. .......... ......CCAC GCCCGGCCCT

LiSIDER2-32-802492r TCGCGGCTCC AAC.GCATC. .......... ......CCAC GCCCGGCCCT

LiSIDER2-32-827594d TCGCGGCTCC AAC.GCATC. .......... ......CCAC GCCCGGCCCT

LiSIDER2-32-808837r TCGCGGCTCC AAC.GCATC. .......... ......CCAC GCCCGGCCCT

LiSIDER2-32-769406r GCATGACTCG AGC.GTAGG. .......... ......TCAC TTCCGACCCT

LiSIDER2-32-881720r GCGTGGCTCG AAC.GCCGT. .......... ....ATTCCA CCGC.CGGCC

LiSIDER2-32-890515r GCGTGGCTCG AAC.GCCGT. .......... ....ATTCCA CCGC.CGGCC

LiSIDER2-32-1496187d GCGTGACTCG AGC.GCACG. .......... ....TCTCAC CCCC.TGGTT

LiSIDER2-32-1532945d GCGTGACTCG AGC.GTACG. .......... ....CCTCAC CCCC.TGGTT

LiSIDER2-32-1545347d GCGT..CACG AAC.ATATA. .......... ....TCTCAC CCCCACGGCC

LiSIDER2-32-772687r GGGGAGCCGG GAAGGCCTT. .......... ....CCATCC CTGCCAATAT

LiSIDER2-32-1141454r GGCCGCTGGC AATTTCGCGA .......... ....AGGTGG CCCAGGCGTA

LiSIDER2-32-354985d CACACT..GC GCACTGGTG. TGGG...... .....GCG.. CCTGAGCC..

LiSIDER2-32-367858d CACACT..GC GCACTGGTG. TGGG...... .....GCG.. CCTGAGCC..

LiSIDER2-32-661708r CGCACT..GC CTAGAGGTG. TGGG...... .....GAGAG CCCGAGCCAC

LiSIDER2-32-1405232d CACACT..GC CCGCTGGTG. TGGG...... .....GAG.. .CCGGTCC..

LiSIDER2-32-169517r CTGGCT..GC CTGCTGGTG. GGGT...... .....GGGGG CTCGGGCCAC

LiSIDER2-32-185886r CTGGCT..GC CTGCTGGTG. GGGT...... .....GGGGG CTCGGGCCAC

LiSIDER2-32-420911d CACACG..GT CTACTGGTG. CGGG...... .....ACGCC TGCGTGTGAC

LiSIDER2-32-460150r CACACG..GT CTACTGGTG. CGGG...... .....ACGCC TGCGTGTGAC

LiSIDER2-32-456060d CACACG..GT CTACTGGTG. CGGG...... .....ACGCC TGCGTGTGAC

LiSIDER2-32-991979r CGCACT..GC CTGCTGGTG. TGCG...... .....AGGAG CCTGCGCCGC

LiSIDER2-32-1017837r CGCACT..GC CTGCTGGTG. TGCG...... .....AGGAG CCTGCGCCGC

LiSIDER2-32-18439r TACACC..GC CCACTGGTG. TGCGGGC... ...GGGGGGA GGGCGGGGGC

LiSIDER2-32-30662r TACACC..GC CCACTGGTG. TGCGGGC... ...GGGGGGA GGGCGGGGGC

LiSIDER2-32-39696d TACACC..GC CTACTGGTG. TGCGGGC... ...GGGGGGA GGGCGGGGGC

LiSIDER2-32-72349d TACACC..GC CTACTGGTG. TGCGGGC... ...GGGGGGA GGGCGGGGGC

LiSIDER2-32-46425r TACACC..GC CTACTGGTG. TGCGGGC... ...GGGGGGA GGGCGGGGGC

LiSIDER2-32-84516r TACACC..GC CCACTGGTG. TGCGGGC... ...GGGGGGA GGGCGGGGGC

LiSIDER2-32-121058r TACACC..GC CCACTGGTG. TGCGGGC... ...GGGGGGA GGGCGGGGGC

LiSIDER2-32-127617r TACACC..GC CCACTGGTG. TGCGGGC... ...GGGG... ........GC

LiSIDER2-32-1128071r CACACT..GC CTGCTGGTG. TGGGGAG... ...TCTGAGC A.......AC

LiSIDER2-32-222503d CGC..T..GG CCACTGGTG. TGGGGA.... ...GCCTGAG ......CCAC

LiSIDER2-32-232971d CGC..T..GG CCACTGGTG. TGGGGA.... ...GCCTGAG ......CCAC

LiSIDER2-32-253342d CGC..T..GG CCACTGGTG. TGGGGA.... ...GCCTGAG ......CCAC

LiSIDER2-32-311078r CACACT..GC CTGCTGGTG. TGGGGA.... ...GCCGGAG T....GCCAC

LiSIDER2-32-1195026d CGCACT..GC CTGCTGGTG. TAGGGAA... ...GCCTGAG ......CGAC

LiSIDER2-32-1219109r CGCACT..GC CTGCTGGTG. TAGGGAA... ...GCCTGAG ......CGAC

LiSIDER2-32-1172151d CGCACT..GC CTGCTGGTG. TAGGGAA... ...GCCTGAG ......CGAC

LiSIDER2-32-542863d CACACG..GC CTACTAGTG. TGGGTGGGTG GGTGGGGGC. ..........

LiSIDER2-32-546470r CACACG..GC CTACTAGTG. TGGGTGGGTG GGTGGGGGGC TGAGTGCCAC

LiSIDER2-32-520410d CACACG..GC CTACTAGTG. TGGGTGGGTG GGTGGGGGGC TGAGTGCCAC

LiSIDER2-32-588727r CACACG..GC CTACTAGTG. TGGGTGGGTG GGTGGGGGGC TGAGTGCCAC

LiSIDER2-32-642986d CACACG..GC CTACTGGTG. TGGGTGGGTG GGTGGGGGGC TGAGTGCCAC

LiSIDER2-32-598168r CACACG..GC CTACTAGTG. TGGGTGGGTG GGTGGGGGGC TGAGTGCCAC

LiSIDER2-32-919839r CGCGCACTGC CTGCTGGTG. TGCA...... ...GGGAGCC T..GCGCCAC

LiSIDER2-32-953057r CGCGCACTGC CTGCTGGTG. TGCA...... ...GGGAGCC T..GCGCCAC

LiSIDER2-32-937896d CGCGCACTGC CTGCTGGTG. TGCA...... ...GGGAGCC T..GCGCCAC

LiSIDER2-32-726312r CGCACT..GC CCAGTGGTG. TGGGGCGGC. ....GGAGCG CCACCGCGAG

LiSIDER2-32-752616r CGCACT..GC CCAGTGGTG. TGGGGCGGC. ....GGAGCG CCACCGCGAG

LiSIDER2-32-734536r CGCACT..GC CCAGTGGTG. TGGGGCGGC. ....GGAGCG CCACCGCGAG

LiSIDER2-32-755765r CGCACT..GC CCAGTGGTG. TGGGGCGGC. ....GGAGCG CCACCGCGAG

LiSIDER2-32-1284330d CAGT....GC CCACTCGTG. TGGGC..... .....GAGCC T..GAGCCGC

LiSIDER2-32-775723r A..ACT..GC CCACTGGTG. TGGG...... .....GAGCC CGAGTACCAT

LiSIDER2-32-794401r A..ACT..GC CCACTGGTG. TGGG...... .....GAGCC CGAGTACCAT

LiSIDER2-32-802492r A..ACT..GC CCACTGGTG. TGGG...... .....GAGCC CGAGTACCAT

LiSIDER2-32-827594d A..ACT..GC CCACTGGTG. TGGG...... .....GAGCC CGAGTACCAT

LiSIDER2-32-808837r A..ACT..GC CCACTGGTG. TGGG...... .....GAGCC CGAGTACCAT

LiSIDER2-32-769406r CTCACT..GT CTAGTGTTG. CGGG...... .....GAGGC TGATTGTAAC

LiSIDER2-32-881720r CTCGCT..GC CCACCGGCG. A..A...... .....GGCCG CCTAAGCCAC

LiSIDER2-32-890515r CTCGCT..GC CCACCGGCG. A..A...... .....GGCCG CCTAAGCCAC

LiSIDER2-32-1496187d CACACC..GT TCGCTGGTG. T..G...... .....GG... ..........

LiSIDER2-32-1532945d CACACC..GT TCGCTGGTG. T..G...... .....GG... ..........

LiSIDER2-32-1545347d CGGATT..GT GCACTAGCG. TCAA...... .....GAGCC TGCGCGCCAC

LiSIDER2-32-772687r CGAGCTGTTT CTGGTGCTGA CAGGTTCAAG CACTTAGAAC TTAGGGGTAA

LiSIDER2-32-1141454r CAAGCGCGCC TCTGAAATGT CCCTCAAGA. ...ACAAGAG CGAGAGTGAC

LiSIDER2-32-354985d ..ACCGCGAG CGGTGTG... ....TGTGTG CG.CGCACCA C.G.......

LiSIDER2-32-367858d ..ACCGCGAG CGGTGTG... ....TGTGTG CG.CGCACCA C.G.......

LiSIDER2-32-661708r ACCCCGAGGG GGATGCACCA CGAGTGGCGA CC.GGCACAA G.GGGGAGCG

LiSIDER2-32-1405232d .CATCACGGG AGGCGCGCCA C..GCGACGG CC.CGCAAAA A.GCAGAGT.

LiSIDER2-32-169517r CCCAC...GG CGGGATGCAC CAGGTGGCC. ACCAGCATGA ..TTGGAGTG

LiSIDER2-32-185886r CCCAC...GG CGGGATGCAC CAGGTGGCC. ACCAGCATGA ..TTGGAGTG

LiSIDER2-32-420911d CCCGA...GA .GAGGTGCGC CAGGTGGCG. GCCAGCATGG ..TGGGAGCC

LiSIDER2-32-460150r CCCGA...GA .GAGGTGCGC CAGGTGGCG. GCCAGCATGG ..TGGGAGCC

LiSIDER2-32-456060d CCCGA...GA .GAGGTGCGC CAGGTGGCG. GCCAGCATGG ..TGGGAGCC

LiSIDER2-32-991979r CCCGA...GG .AACGTGC.. CAGGTAGCCC AGTTGCACGA ..TGGGAGCG

LiSIDER2-32-1017837r CCCGA...GG .AACGTGC.. CAGGTAGCCC AGTTGCACGA ..TGGGAGCG

LiSIDER2-32-18439r AGCGG..CGC CCGCGCCCAT TCCGAGAAGG GA.GGGA.GG ..CACGAGCT

LiSIDER2-32-30662r AGCGG..CGC CCGCGCCCAT TCCGAGAAGG GA.GGGA.GG ..CACGAGCT

LiSIDER2-32-39696d AGCGG..CGC CCGCGCCCAT TCCGAGAAGG GA.GGGA.GG ..CACGAGCT

LiSIDER2-32-72349d AGCGG..CGC CCGCGCCCAT TCCGAGAAGG GA.GGGA.GG ..CACGAGCT

LiSIDER2-32-46425r AGCGG..CGC CCGCGCCCAT TCCGAGAAGG GA.GGGA.GG ..CACGAGCT

LiSIDER2-32-84516r AGCGG..CGC CCGCGCCCAT TCCGAGAAGG GA.GGGA.GG ..CACGAGCT

LiSIDER2-32-121058r AGCGG..CGC CCGCGCCCAT TCCGAGAAGG GA.GGGA.GG ..CACGAGCT

LiSIDER2-32-127617r AGCGG..CGC CCGCGCCCAT TCCGAGAAGG GA.GGGA.GG ..CACCAGCT

LiSIDER2-32-1128071r CCCGA..GGA TGATGCGCAT CAGGTGGCGA TC.GGCATAA ..TGTGAACC

LiSIDER2-32-222503d CCCGC..GGC GGG.ATGCGC GAGGTGGCGA CC.GGCATAG ..TGGGGGCA

LiSIDER2-32-232971d CCCGC..GGC GGG.ATGCGC GAGGTGGCGA CC.GGCATAG ..TGGGGGCA

LiSIDER2-32-253342d CCCGC..GGC GGG.ATGCGC GAGGTGGCGA CC.GGCATAG ..TGGGGGCA

LiSIDER2-32-311078r ACCGA..GGG GGGGATGCAC CACGTAGCGA CC.GGCACAA ..TGGGAGCG

LiSIDER2-32-1195026d CTCG...... GGCGATGCAT CGCGTGGCGG TC.AGCATGG C.TGGGGGCG

LiSIDER2-32-1219109r CTCG...... GGCGATGCAT CGCGTGGCGG TC.AGCATGG C.TGGGGGCG

LiSIDER2-32-1172151d CTCG...... GGCGATGCAT CGCGTGGCGG TC.AGCATGG C.TGGGGGCG

LiSIDER2-32-542863d .......... .......... .......... .......... ..........

LiSIDER2-32-546470r CGCC...GAT GAGGGGGCAG GGATGCGCCA GG.GG..TGG CGACGGGGCA

LiSIDER2-32-520410d CGCC...GAT GAGGGGGCAG GGATGCGCCA GG.GG..TGG CGACGGGGCA

LiSIDER2-32-588727r CGCC...GAT GAGGGGGCAG GGATGCGCCA GG.GG..TGG CGACGGGGCA

LiSIDER2-32-642986d CGCC...GAT GAGGGGGCAG GGATGCGCCA GG.GG..TGG CGACGGGGCA

LiSIDER2-32-598168r CGCC...GAT GAGGGGGCAG GGATGCGCCA GG.GG..TGG CGACGGGGCA

LiSIDER2-32-919839r .GCC...GAG GAGGATGCAC CGCGTGGCGG CC.GG..CAC GATGGGCGCG

LiSIDER2-32-953057r .GCC...GAG GAGGATGCAC CGCGTGGCGG CC.GG..CAC GATGGGCGCG

LiSIDER2-32-937896d .GCC...GAG GAGGATGCAC CGCGTGGCGG CC.GG..CAC GATGGGCGCG

LiSIDER2-32-726312r TGTG...TGT GTGTGTGCAG CACGTAGGGA CC.GACATGA TGTGGGAGCG

LiSIDER2-32-752616r TGTG...TGT GTGTGTGCAG CACGTAGGGA CC.GACATGA TGTGGGAGCG

LiSIDER2-32-734536r TGTG...TGT GTGTG..CAG CACGTAGGGA CC.GACATAA TGTGGGAGCG

LiSIDER2-32-755765r TGTG...TGT GTGTGTGCAG CACGTAGGGA CC.GACATGA TGTGGGAGCG

LiSIDER2-32-1284330d C.CT...GAG GGGGGAGCAC CACGTGGTGA CT.GG..CAC AATGGGAGCG

LiSIDER2-32-775723r CCCTGATGGT GGACACACAC CGTGCGGCGA CC.GGCATGA TGGGGGAGCG

LiSIDER2-32-794401r CCCTGATGGT GGACACACAC CGTGCGGCGA CC.GGCATGA TGGGGGAGCG

LiSIDER2-32-802492r CCCTGATGGT GGACACACAC CGTGCGGCGA CC.GGCATGA TGGGGGAGCG

LiSIDER2-32-827594d CCCTGATGGT GGACACACAC CGTGCGGCGA CC.GGCATGA TGGGGGAGCG

LiSIDER2-32-808837r CCCTGATGGT GGACACACAC CGTGCGGCGA CC.GGCATGA TGGGGGAGCG

LiSIDER2-32-769406r CCC....GGT GGGCGCACCA GGGGTGGCGG CTTGGCATGA TGGGG.AGCG

LiSIDER2-32-881720r CCCGA..... .GGGATGCGC CAGGTGAC.. ...GACCTGC ....GAGGCG

LiSIDER2-32-890515r CCCGA..... .GGGATGCGC CAGGTGAC.. ...GACCTGC ....GAGGCG

LiSIDER2-32-1496187d .......... .......... .......... ...GGCCTGC ....GGGGCG

LiSIDER2-32-1532945d .......... .......... .......... ...GGCCTGC ....GGGGCG

LiSIDER2-32-1545347d CGCGA..... .GGGACAGAC ACAAGGAGAC GA.GGCGAGC ....GGAGCG

LiSIDER2-32-772687r GCCCGAGTGA TGCATCGCTG CTGATGTCGG CG.GTCAGGC GCTGGCCGGC

LiSIDER2-32-1141454r CAGGC..GGT GGAGATGGAG GAGGCAGCAA A..GGCATTC GTCAAGGCCG

LiSIDER2-32-354985d .C...GGTGA GCTTCTCAGC GGGG...... .......... AGGTGGGCAG

LiSIDER2-32-367858d .C...GGTGA GCTTCTCAGC GGGG...... .......... AGGTGGGCAG

LiSIDER2-32-661708r GC...GGTGA GGCGACCTGC GAGGG..... .GCGGGGTGT AGGTGGGTAG

LiSIDER2-32-1405232d .C...GGCAA C...ACTTGC G......... .......... ..GTGGAGAC

LiSIDER2-32-169517r GC...TGTGA GGCGACCTTC GGAG...... CGGTGG.... ..ATGGGTGG

LiSIDER2-32-185886r GC...TGTGA GGCGACCTTC GGAG...... CGGTGG.... ..ATGGGTGG

LiSIDER2-32-420911d GCGGGTGTGA GGCAGCCTGC GCAG...... CGGCGGTGGA TGATGAGTCG

LiSIDER2-32-460150r GCGGGTGTGA GGCAGCCTGC GCAG...... CGGCGGTGGA TGATGAGTCG

LiSIDER2-32-456060d GCGGGTGTGA GGCAGCCTGC GCAG...... CGGCGGTGGA TGATGAGTCG

LiSIDER2-32-991979r GC...TGTGA GGCGACCTGC GGAG...... CGGCGTGGGT GTGTGGGCAG

LiSIDER2-32-1017837r GC...TGTGA GGCGACCTGC GGAG...... CGGCGTGGGT GTGTGGGCAG

LiSIDER2-32-18439r GG...CGGCC GGCGAGGCGG CGGG...... ......TGG. .GTGGGGTAG

LiSIDER2-32-30662r GG...CGGCC GGCGAGGCGG CGGG...... ......TGG. .GTGGGGTAG

LiSIDER2-32-39696d GG...CGGCC GGCGAGGCGG CGGG...... ......TGG. .GTGGGGTAG

LiSIDER2-32-72349d GG...CGGCC GGCGAGGCGG CGGG...... ......TGG. .GTGGGGTAG

LiSIDER2-32-46425r GG...CGGCC GGCGAGGCGG CGGG...... ......TGG. .GTGGGGTAG

LiSIDER2-32-84516r GG...CGGCC GGCGAGGCGG CGGG...... ......TGG. .GTGGGGTAG

LiSIDER2-32-121058r GG...CGGCC GGCGAGGCGG CGGG...... ......TGG. .GTGGGGTAG

LiSIDER2-32-127617r GG...CGACC GGCGAGGCGG CGAG...... ......TGG. .GTGGGGTAG

LiSIDER2-32-1128071r GC...TGTGA GGCAAC.CGG CGGA...... ......GAG. .GGTGGGCAG

LiSIDER2-32-222503d AC...TGTGG TGCTGACTGC GAGG...... ......CGGC GGGTGGGTAG

LiSIDER2-32-232971d AC...TGTGG TGCTGACTGC GAGG...... ......CGGC GGGTGGGTAG

LiSIDER2-32-253342d AC...TGTGG TGCTGACTGC GAGG...... ......CGGC GGGTGGGTAG

LiSIDER2-32-311078r GC...TGTGA GGCGACCTTC GGAG...... ......CGG. AGGTCGCTAA

LiSIDER2-32-1195026d AC...GGTGA GGCAGCCTGC GAGG...... ......CGG. GGGCGGGAGG

LiSIDER2-32-1219109r AC...GGTGA GGCAGCCTGC GAGG...... ......CGG. GGGCGGGAGG

LiSIDER2-32-1172151d AC...GGTGA GGCAGCCTGC GAGG...... ......CGG. GGGCGGGAGG

LiSIDER2-32-542863d .......... .......... .......... .......... ..........

LiSIDER2-32-546470r TG...ATGGA CGCGGCTGTG AGGGACCCTG CGCTGCGGGC CTCTGGGTAT

LiSIDER2-32-520410d TG...ATGGA CGCGGCTGTG AGGGACCCTG CGCTGCGGGC CTCTGGGTAT

LiSIDER2-32-588727r TG...ATGGA CGCGGCTGTG AGGGACCCTG CGCTGCGGGC CTCTGGGTAT

LiSIDER2-32-642986d TG...ATGGG CGAGGGTGG. .......... .......... ...TAAGTAG

LiSIDER2-32-598168r TG...ATGGA CGCGGCTGTG AGGGACCCTG CGCTGCGGGC CTCTGGGTAT

LiSIDER2-32-919839r GG...TCGGG GGTGGGTGGG .......... .......... ..TGGGGCCG

LiSIDER2-32-953057r GG...TCGGG GGTGGGTGGG .......... .......... ..TGGGGCCG

LiSIDER2-32-937896d GG...TCGGG GGTGGGTGGG .......... .......... ..TGGGGCCG

LiSIDER2-32-726312r GC...TGTGA GGCGGCCTGC GAGG...... CAGGATGGGT GGGCGGACAG

LiSIDER2-32-752616r GC...TGTGA GGCGGCCTGC GAGG...... CAGGATGGGT GGGCGGGCAG

LiSIDER2-32-734536r GC...TGTGA GGCGGCCTGC GAGG...... CAGGATGGGT GGGCGGGCAG

LiSIDER2-32-755765r GC...TGTGA GGCGGCCTGC GAGG...... CAGGATGGGT GGGCGGGCAG

LiSIDER2-32-1284330d GC...TGTGA GGCAACCTGG GAGA...... ...CGGCGGG TGGTGGGTGG

LiSIDER2-32-775723r GC...CTGCG AGTCGGCCTG CGAG...... ...GCGGCCG TGGTGGGTAG

LiSIDER2-32-794401r GC...CTGCG AGTCGGCCTG CGAG...... ...GCGGCCG TGGTGGGTAG

LiSIDER2-32-802492r GC...CTGCG AGTCGGCCTG CGAG...... ...GCGGCCG TGGTGGGTAG

LiSIDER2-32-827594d GC...CTGCG AG........ .......... ...GCGGCCG TGGTGGGTAG

LiSIDER2-32-808837r GC...CTGCG AGTCGGCCTG CGAG...... ...GCGGCCG TGGTGGGTAG

LiSIDER2-32-769406r GC...CTGCG AG........ .......... ...GCGGCCG TGGTGGGTAG

LiSIDER2-32-881720r GG......CG GGTG...... .......... .......... .GGTG...GC

LiSIDER2-32-890515r GG......CG GGTG...... .......... .......... .GGTG...GC

LiSIDER2-32-1496187d GG....GGTG GGCA...... .......... .......... .GGTG...GC

LiSIDER2-32-1532945d GG....GGTG GGCA...... .......... .......... .GGTG...GC

LiSIDER2-32-1545347d GG....GGTG GGTG...... .......... .......... .TGTGCGTAG

LiSIDER2-32-772687r GATTTTCTGA AGCGACCCGC AAGG...... .......AGT TGGTCGATGG

LiSIDER2-32-1141454r GCGA.TGCAA AGTCAGCAGC AGCC...... CTGCTCAGAG ACGTGGTGGA

LiSIDER2-32-354985d ACTTTGAGGC A....GGGGC CGTGCTGA.. GGTGACTGGG TTGGC.GGAG

LiSIDER2-32-367858d ACTTTGAGGC A....GGGGC CGTGCTGA.. GGTGACTGGG TTGGC.GGAG

LiSIDER2-32-661708r TGTTCGAGGC A....GACGC CCTACTCG.. GGTGATTGAG TCGGT.GCAT

LiSIDER2-32-1405232d TGTGCT.... .......... ....TTCC.. GATGACGGAG TTGGC.GCAT

LiSIDER2-32-169517r AGCTCGAGGC A....GAATG CGTGCTGT.. GATGGCTGAG TCGGC.GCAT

LiSIDER2-32-185886r AGCTCGAGGC A....GAATG CGTGCTGT.. GATGGCTGAG TCGGC.GCAT

LiSIDER2-32-420911d AGGTTGGCGC A....GAGGC CGTGCTCA.. GGCCGTCGAG CCGGT.GCGT

LiSIDER2-32-460150r AGGTTGGCGC A....GAGGC CGCGCTCA.. GGCCGCCGAG CCGGT.GCGT

LiSIDER2-32-456060d AGGTTGGCGC A....GAGGC CGCGCTCA.. GGCCGCCGAG CCGGT.GCGT

LiSIDER2-32-991979r CGTTTGAGGC A....GAGAC CGTTCTCC.. GATGACTGAG TCGGC.GCAT

LiSIDER2-32-1017837r CGTTTGAGGC A....GAGAC CGTTCTCC.. GATGACTGAG TCGGC.GCAT

LiSIDER2-32-18439r AGCTCGAGGG CA..GATGGC CGTGCTCA.. GATAGCTGAG TCGGCAGCAC

LiSIDER2-32-30662r AGCTCGAGGG CA..GATGGC CGTGCTCA.. GATGGCTGAG TCGGCAGCAC

LiSIDER2-32-39696d AGCTCGAGGG CA..GATGGC CGTGCTGG.. GATGACCGGG TCGGC.GCAC

LiSIDER2-32-72349d AGCTCGAGGG CA..GGTGGC CGTGCTGG.. GATGACCGGG TCGGC.GCAC

LiSIDER2-32-46425r AGCTCGAGGG CA..GATGGC CGTGCTGG.. GATGACCGGG TCGGC.GCAC

LiSIDER2-32-84516r AGCTCGAGGG CA..GGTGGC CGTGCTCA.. GATAGCTGAG TCGGC.GCAC

LiSIDER2-32-121058r AGCTCGAGGG CA..GATGGC CGTGCTGG.. GATGACCGGG TCGGC.GCAC

LiSIDER2-32-127617r AGCTCGAGGG CA..GGTGGC CGTGCTGG.. GATGACCGGG TCGGC.GCAC

LiSIDER2-32-1128071r AATTCGAGG. CA..GG.GGC CGTGCTCG.. GGTGACTGAG TCGGC.GCAT

LiSIDER2-32-222503d AGCTTGAGGG CA..GGGGGC CGTGCTCG.. GATGGCTGGG TCGGC.GCAC

LiSIDER2-32-232971d AGCTTGAGGG CA..GGGGGC CGTGCTCG.. GATGGCTGGG TCGGC.GCAC

LiSIDER2-32-253342d AGCTTGAGGG CA..GGGGGC CGTGCTCG.. GGTGGCTGGG TCGGC.GCAC

LiSIDER2-32-311078r AGTGTGAAAC ....GAAGGC TGCACTCC.. CATGACCAAG TCGTC.GCAT

LiSIDER2-32-1195026d TGTTTGGGGC ....GCAGAC GCTGCCCG.. CATGCCTGAG CTGGC.GCAT

LiSIDER2-32-1219109r TGTTTGGGGC ....GCAGAC GCTGCCCG.. CATGCCTGAG CTGGC.GCAT

LiSIDER2-32-1172151d TGTTTGGGGC ....GCAGAC GCTGCCCG.. CATGCCTGAG CTGGC.GCAT

LiSIDER2-32-542863d .......... .......... .......... .......... ..........

LiSIDER2-32-546470r GGTTTGAGGC G....GAGGC GGTGTTCC.. GATGACAGAG TCGGC.GCGT

LiSIDER2-32-520410d GGTTTGAGGC G....GAGGC GGTGTTCC.. GATGACAGAG TCGGC.GCGT

LiSIDER2-32-588727r GGTTTGAGGC G....GAGGC GGTGTTCC.. GATGACAGAG TCGGC.GCGT

LiSIDER2-32-642986d GGTTGGAGGC A....GAGGT GGTGCTCC.. GATGACAGTG TCGGC.GCAT

LiSIDER2-32-598168r GGTTTGAGGC G....GAGGC GGTGTTCC.. GATGACAGAG TCGGC.GCGT

LiSIDER2-32-919839r CGTTTGAGGC A....GGGGT CGTGCTGC.. GATGACTGGG TCGGC.GCAT

LiSIDER2-32-953057r CGTCTGAGGC A....GGGGT CGTGCTGC.. GATGACTGGG TCGGC.GCAT

LiSIDER2-32-937896d CGTTTGAGGC A....GGGGT CGTGCTGC.. GATGACTGGG TCGGC.GCAT

LiSIDER2-32-726312r AGCTTGAGGG CAG.TGGGGC CGTTCTCC.. GATGACTGAG TGGGC.ACAC

LiSIDER2-32-752616r AGCTTGAGGG CAG.TGGGGC CGTTCTCC.. GATGACTGAG TGGGC.ACAC

LiSIDER2-32-734536r AGCTTGAGGG CAG.TGGGGC CGTTCTCC.. GATGACTGAG TGGGC.GCAT

LiSIDER2-32-755765r AGCTTGAGGG CAG.TGGGGC CGTTCTCC.. GATGACTGAG TGGGC.GCAT

LiSIDER2-32-1284330d AGTTCGAGGC A....GGGGC CGTGCTCTCA GATGGCTGGG CCGGT.GCAT

LiSIDER2-32-775723r AGTTTGAGGC A....GAGGC CATGCTCA.. GATGGCTGAG TCGGC.GCAT

LiSIDER2-32-794401r AGTTTGAGGC A....GAGGC CATGCTCA.. GATGGCTGAG TCGGC.GCAT

LiSIDER2-32-802492r AGTTTGAGGC A....GAGGC CATGCTCA.. GATGGCTGAG TCGGC.GCAT

LiSIDER2-32-827594d AGTTTGAGGC A....GAGGC CATGCTCA.. GATGGCTGAG TCGGC.GCAT

LiSIDER2-32-808837r AGTTTGAGGC A....GAGGC CATGCTCA.. GATGGCTGAG TCGGC.GCAT

LiSIDER2-32-769406r AGTTTGAGGC A....GAGGC CATGCTCA.. GATGGCTGAG TCGGC.GCAT

LiSIDER2-32-881720r GATCGGAGGC A....GGGGC CGTGCTCC.. GGTGGGTGAG TTGGC.GCAT

LiSIDER2-32-890515r GATCGGAGGC A....GGGGC CGTGCTCC.. GGTGGGTGAG TTGGC.GCAT

LiSIDER2-32-1496187d G.TTTGAGGC A....GGGAC CGTGCTCG.. GCTGCCTGCA TCGGCAGCAT

LiSIDER2-32-1532945d G.TTTGAGGC A....GGGAC CGTGCTCG.. GCTGCCTGCA TCGGCAGCAT

LiSIDER2-32-1545347d AGTTTGAGGC A....AGGGC CGTTCTGA.. GCCAATTGCG ACGGT.GCGC

LiSIDER2-32-772687r ACTTTGAGGC A......GGG TGCGTTCAGA GATGACCGAG TGGGC.GCAT

LiSIDER2-32-1141454r CATGTATGAC AAGGCTCAGA AGTACACAAA CGCAGCCAAG GCGTGCGCTG

LiSIDER2-32-354985d GGCTGCAACG CGTGC..TGA CGGCTGCTTC G.CCACACGT G...ATGGTC

LiSIDER2-32-367858d GGCTGCAACG CGTGC..TGA CGGCTGCTTC G.CCACACGT G...ATGGTC

LiSIDER2-32-661708r TGCTGTAACG CATGACTATA GCGCTTCTTC G.CACCACGC GGATGAGGCC

LiSIDER2-32-1405232d TGCTGGAACG GGCGCGTCTA CGGCTGCCTC G.CACGGCGC G.ATGAGGCC

LiSIDER2-32-169517r TGCCGTAACG CGTGTCTAC. ....TGCATC GTGC.CACGC G.ATGGG..C

LiSIDER2-32-185886r TGCCGTAACG CGTGTCTAC. ....TGCATC GTGC.CACGC G.ATGGG..C

LiSIDER2-32-420911d TGCTGTAACG CGTGTCCACG GGTGCGATCG GCACATACGC G.ATCGCG.T

LiSIDER2-32-460150r TGCTGTAACG CGTGTCCACG GGTGCGATCG GCACATACGC G.ATCGCG.T

LiSIDER2-32-456060d TGCTGTAACG CGTGTCCACG GGTGCGATCG GCACATACGC G.ATCGCG.T

LiSIDER2-32-991979r TGTTGCAACG CCGGTGCCTA GCGCTGCTGC GCAC.CATGC G.ATGGGGGC

LiSIDER2-32-1017837r TGTTGCAACG CCGGTGCCTA GCGCTGCTGC GCAC.CATGC G.ATGGGGGC

LiSIDER2-32-18439r TGCTGCACCG CG..TGCCTG CCGCTTCTTC G.CGCGACGC G.GGGTGGGA

LiSIDER2-32-30662r TGCTGCACCG CG..TGCCTG CCGCTTCTTC G.CGCGACGC G.GGGTGGGA

LiSIDER2-32-39696d TGCTGCAACG CGCGTGCCTG CCGCTTCTTC G.CGCGACGC G.GGGTGGGA

LiSIDER2-32-72349d TGCTGCAACG CGCGTGCCTG CCGCTTCTTC G.CGCGACGC G.GGGTGGGA

LiSIDER2-32-46425r TGCTGCAACG CGCGTGCCTG CCGCTTCTTC G.CGCGACGC G.GGGTGGGA

LiSIDER2-32-84516r TGCTGCACCG CG..TGCCTG CCGCTTCTTC G.CGCGACGC G.GGGTGGGA

LiSIDER2-32-121058r TGCTGCAACG CGCGTGCCTT GCGCTGCTTC G.CCCCACAC G.ATG.GGGT

LiSIDER2-32-127617r TGCTGCAACG CGCGTGCCTT GCGCTGCTTC G.CCCCACAC G.ATG.GGGT

LiSIDER2-32-1128071r TGCTGTAGCA CG..TGCCCA CGGCTGCTTT G.CACGACGC A.AGGTGGGC

LiSIDER2-32-222503d GACTGTGATG CG..TGTCGA GTGCTGCTTC G.CGCGACGC G.A..TGGGC

LiSIDER2-32-232971d GACTGTGATG CG..TGTCGA GTGCTGCTTC G.CGCGACGC G.A..TGGGC

LiSIDER2-32-253342d GACTGTGATG CG..TGTCGA GTGCTGCTTC G.CGCGACGC G.A..TGGGC

LiSIDER2-32-311078r GGCTGTGGCG CG..TCTCTT GTGCTGCTTC G.CACCACAC G.A..TGGCC

LiSIDER2-32-1195026d TGCTGCCACG CG..TGTGCG CGGCTGCTTC G.CGGCGTGC G.A..TGGGT

LiSIDER2-32-1219109r TGCCGCCACG CG..TGTGCG CGGCTGCTTC G.CGGCGTGC G.A..TGGGT

LiSIDER2-32-1172151d TGCTGCCACG CG..TGTGCG CGGCTGCTTC G.CGGCGTGC G.A..TGGGT

LiSIDER2-32-542863d .......... .......... .......... .......... ..........

LiSIDER2-32-546470r TGCTGTAAGG CTATTGC..A GCGCTGCTTC GCACGACGCG A.TGGGGAGG

LiSIDER2-32-520410d TGCTGTAAGG CTATTGC..A GCGCTGCTTC GCACGACGCG A.TGGGGAGG

LiSIDER2-32-588727r TGCTGTAAGG CTATTGC..A GCGCTGCTTC G.CACGACGC G.ATGGGGAG

LiSIDER2-32-642986d TACTCCAGCA CGGGTTT..A TTGCTCCTTG GGCGCGACGC ..........

LiSIDER2-32-598168r TGCTGTAAGG CTATTGC..A GCGCTGCTTC G.CACGACGC G.ATGGGGAG

LiSIDER2-32-919839r TGCTGTGACG CGCGTGTCTA GCGCTGCTGC A.CACCACGC G.AGGCGGAC

LiSIDER2-32-953057r TGCTGTGACG CGCGTGTCTA GCGCTGCTGC A.CACCACGC G.AGGCGGGC

LiSIDER2-32-937896d TGCTGTGACG CGCGTGTCTA GCGCTGCTGC A.CACCACGC G.AGGCGGGC

LiSIDER2-32-726312r TGCCGCAACG CCTGGCGAAA GCGATGGTTG A.CAGCACGC G.CTGAGGGA

LiSIDER2-32-752616r TGCCGCAACG CCTGGCGAAA GCGATGGTTG A.CAGCACGC G.CTGAGGAA

LiSIDER2-32-734536r GGCTGTAACA GC.ATCTCCA CGGTTGCCTG G.CGCGGCGC G.ATGTGGGG

LiSIDER2-32-755765r GGCTGTAACA GC.ATCTCCA CGGCTGCCTG G.CGCGGCGC G.ATGTGGGG

LiSIDER2-32-1284330d TGCTGCAACG CGTGTTTC.. ACGCTGCTCC G.CATCACGC G.........

LiSIDER2-32-775723r TGCTGTAGCA CGTGTGTCTC ACGCTGCTTC G.CACCAGGC G.ATGCGGTG

LiSIDER2-32-794401r TGCTGCAGCA CGTGTGTCTC ACGCTGCTTC G.CACCAGGC G.ATGCGGTG

LiSIDER2-32-802492r TGCTGCAGCA CGTGTGTCTC ACGCTGCTTC G.CACCAGGC G.ATGCGGTG

LiSIDER2-32-827594d TGCTGCAGCA CGTGTGTCTC ACGCTGCTTC G.CACCAGGC G.ATGCGGTG

LiSIDER2-32-808837r TGCTGCAGCA CGTGTGTCTC ACGCTGCTTC G.CACCAGGC G.ATGCGGTG

LiSIDER2-32-769406r TGCTGTAGCA CGTGTGTCTC ACGCTGCTTC G.CACCAGGC G.ATGCGGTG

LiSIDER2-32-881720r TGCTGTAATG CG..TCTCTT GTGCTGCCTC A.CACCAGGC GATG..GGGC

LiSIDER2-32-890515r TGCTGTAATG CG..TCTCTT GTGCTGCCTC A.CACCAGGC GATG..GGGC

LiSIDER2-32-1496187d AGCTGCAACG CGCGTGTCTC CGGCTTCTCC G.CATCACGC GATGTGGGGC

LiSIDER2-32-1532945d AGCTGCAACG CGCGTGTCTC CGGCTTCTCC G.CATCACGC GATGTGGGGC

LiSIDER2-32-1545347d TACTGCAACG CGTGTGTCTG AGGCTGTTTG C.TACGATGT GATG...GAT

LiSIDER2-32-772687r TGCCGCAACG CCTGGCGAAA GCGATGGCTG A.CAGCACGC G.ATGAGGGC

LiSIDER2-32-1141454r CGCTGGGCGA CATAACCATG G....GCGAC GAGGCGATGC G.....GTGG

LiSIDER2-32-354985d CTGTTGCAGG CCGGG.GTGG ACAGAGTGGT ATTCAAGCTA TGCTGTATGG

LiSIDER2-32-367858d CTGTTGCAGG CCGGG.GTGG ACAGAGTGGT ATTCAAGCTA TGCTGTATGG

LiSIDER2-32-661708r C.GTGAAAGG CCTGG.GCGG G.AGGGAGGT TTGACGCTCA TGCACGGTGG

LiSIDER2-32-1405232d CTGTCGCAGG GC........ .......... .......... ..........

LiSIDER2-32-169517r CTGTGACAGG .CCGG...GT GGAGTGGCGT TGAG....CT CATGCCCGAT

LiSIDER2-32-185886r CTGTGACAGG .CCGG...GT GGAGTGGCGT TGAG....CT CATGCCCGAT

LiSIDER2-32-420911d CTGTGACAGA .CTGGTGGGC AGCGGGGCGT GCGA....CG CACGCTTTAT

LiSIDER2-32-460150r CTGTGACAGA .CTGGTGGGC AGCGGGGCGT GCGA....CG CACGCTTTAT

LiSIDER2-32-456060d CTGTGACAGA .CTGGTGGGC AGCGGGGCGT GCGA....CG CACGCTTTAT

LiSIDER2-32-991979r CTATGACAGG GCCGGGGCGT GGGGGGGGGA GGGAAGGTCG AGTGGCATCG

LiSIDER2-32-1017837r CTATGACAGG GCCGGGGCGT GGGGGGGGGA GGGAAGGTCG AGTGGCATCG

LiSIDER2-32-18439r CTGTGACAGG CCGTG....G GTGGAGCGGA GCTTGCCCTG TGTTGGAGTG

LiSIDER2-32-30662r CTGTGACAGG CCGTG....G GTGGAGCGGA GCTTGCCCTG TGTTGGAGTG

LiSIDER2-32-39696d CTGTGACAGG CCGTG....G GTGGAGCGGA GCTTGCCTTG TGTTGGAGTG

LiSIDER2-32-72349d CTGTGACAGG CCGTG....G GTGGAGCGGA GCTTGCCTTG TGTTGGAGTG

LiSIDER2-32-46425r CTGTGACAGG CCGTG....G GTGGAGCGGA GCTTGCCTTG TGTTGGAGTG

LiSIDER2-32-84516r CTGTGACAGG CCGTG....G GTGGAGCGGA GCTTGCCTTG TGTTGGAGTG

LiSIDER2-32-121058r CTGTGACAGG CCGGGTGGGG GTAGAGTAGA GTTTCACTTA TATTTCGGGG

LiSIDER2-32-127617r CTGTGACAGG CCGGGTGGGG GTAGAGTGG. .......... ..........

LiSIDER2-32-1128071r CGGAGGCGGG CG.......G GTAGAGCGCA GTTG...... ..........

LiSIDER2-32-222503d CTGCGACATG CCGGGCG..A GTAGGGTGGG CCTGCACTCA TGTTGTCTAG

LiSIDER2-32-232971d CTGCGACATG CCGGGCG..A GTAGGGTGGG CCTGCACCCA TGTAGTCTAG

LiSIDER2-32-253342d CTGCGACATG CCGGGCG..A GTAGGGTGGG CCTGCACCCA TGTAGTCTAG

LiSIDER2-32-311078r CTGCGACAAA CTGGGGG..A A.GGAGTGGG GGGGGGTGAA TTGAA.....

LiSIDER2-32-1195026d CTCTGACG.. ..GGGCG..G GCGGGGCGGG GCTCAACGTG TGTGCTGTGG

LiSIDER2-32-1219109r CTCTGACG.. ..GGGCG..G GCGGGGCGGG GCTCAACGTG TGTGCTGTGG

LiSIDER2-32-1172151d CTCTGACG.. ..GGGCG..G GCGGGGCGGG GCTCAACGTG TGTGTTGTGG

LiSIDER2-32-542863d .......... .......... .......... .......... ..........

LiSIDER2-32-546470r AGGGGGCCTG CGGCAGGCCG GGTGCCGAGT GAAGTGTAAC GCATGCTCTG

LiSIDER2-32-520410d AGGGGGCCTG CGGCAGGCCG GGTGCCGAGT GAAGTGTAAC GCATGCTCTG

LiSIDER2-32-588727r GAGGGGGCCT GTGACAGGGG CTTCGGCAAG AGTGTGTGTG TATTTGTGGC

LiSIDER2-32-642986d .......... .......... .......... .......... ..........

LiSIDER2-32-598168r GAGGGGGCCT GCGGCAGGCG GGGCAAATTG GAGGTAAAGC CGTGCTGTGC

LiSIDER2-32-919839r CTGTGACGGG GCGCGTGCGG GCTCGAGTTG GGTTTGGACT CCTGCTGTAC

LiSIDER2-32-953057r CTGTGACGGG GCGCGTGCGG GCTCGAGTTG GGTTTGGACT CCTGTTGTAT

LiSIDER2-32-937896d CTGTGACGGG GCGCGTGCGG GCTCGAGTTG GGTTTGGGCT CCTGCTGTAT

LiSIDER2-32-726312r CT........ .......... .......... .......... ..........

LiSIDER2-32-752616r CT........ .......... .......... .......... ..........

LiSIDER2-32-734536r CC........ .......... .......... .......... ..........

LiSIDER2-32-755765r CC........ .......... .......... .......... ..........

LiSIDER2-32-1284330d .......... .......... .......... .......... ..........

LiSIDER2-32-775723r CCTGTGAC.. .......... .......... .......... ..........

LiSIDER2-32-794401r CCTGTGACGG GCCAGGGGTA GACTGGAGAG TGACTCATGT GGTACAGCAA

LiSIDER2-32-802492r CCTGTGACGG GCCAGGGGTA GACTGGAGAG TGACTCATGC TTTATGGCAG

LiSIDER2-32-827594d CCTGTGACGG GCCAGGGGTA GACTGGAGAG TGACTCATGT GGTACAGCAA

LiSIDER2-32-808837r CCTGTGACGG GCCAGGGATA GGTTGGCGCT TAGCCGATGC TTTATCTGGG

LiSIDER2-32-769406r CCTTTG.... .......... .......... .......... ..........

LiSIDER2-32-881720r CTGTGGCAGG CCCGGTAGAG CTGACATCAT ATTGTGTCGG AGAGCAATGG

LiSIDER2-32-890515r CTGTGGCAGG CCCGGTAGAG CTGACATCAT ATTGTGTCGG AGAGCAATGG

LiSIDER2-32-1496187d CTGTGACAG. .......... .......... .......... ..........

LiSIDER2-32-1532945d CTGTGGCAG. .......... .......... .......... ..........

LiSIDER2-32-1545347d CCCTGACCGG CGGGGCAGAC ACGGA..... .......... ..........

LiSIDER2-32-772687r CTTTGGCAAG CCTGG....G CTAGTGTGGA GTATAGCGCA TGCTCGCTGA

LiSIDER2-32-1141454r CTGCAACAGG CC........ .......... .......... ..........

LiSIDER2-32-354985d TAGAGAATGG TCTTGCTGCA AAAGAAGAAA ACGC...... ..........

LiSIDER2-32-367858d TAGAGAATGG TCTTGCTGCA AAAGAAGAAA ACGC...... ..........

LiSIDER2-32-661708r CAGAGAGTCG TCACGCCGCA GAGGAGAAAA AAGCA..... ..........

LiSIDER2-32-1405232d .......... .......... .......... .......... ..........

LiSIDER2-32-169517r GACAGAATG. .......... .......... .......... ..........

LiSIDER2-32-185886r GACAGAATG. .......... .......... .......... ..........

LiSIDER2-32-420911d GGCCAAGTAA TGGA...... .......... .......... ..........

LiSIDER2-32-460150r GGCCAAGTAA TGGG...... .......... .......... ..........

LiSIDER2-32-456060d GGTCAAGTAA TGGA...... .......... .......... ..........

LiSIDER2-32-991979r GGCTTGACGT TGTGTGCCAG AGAGAAAGCG GACACACGTT GGGAGACGAA

LiSIDER2-32-1017837r GGCTTGACGT TGTGTGCCAG AGAGAAAGCG GACACACGTT GGGAGACCAA

LiSIDER2-32-18439r GCAGAATAGG CCATGTGAAG .......... .......... ..........

LiSIDER2-32-30662r GCAGAGAAAC GAATGCGTTG .......... .......... ..........

LiSIDER2-32-39696d GCAGAGAAAC GAATGCGTTG .......... .......... ..........

LiSIDER2-32-72349d GCAGAGAAAC GAATGCGTTG .......... .......... ..........

LiSIDER2-32-46425r GCAGAATAGG CCATGTGAAA .......... .......... ..........

LiSIDER2-32-84516r GCAG...... .......... .......... .......... ..........

LiSIDER2-32-121058r CGG....... .......... .......... .......... ..........

LiSIDER2-32-127617r .......... .......... .......... .......... ..........

LiSIDER2-32-1128071r .......... .......... .......... .......... ..........

LiSIDER2-32-222503d CAGTGCATGA AGGCACAGAA AAAAAAGAGG .......... ..........

LiSIDER2-32-232971d CAGTGCATGA AGGCACAGAA AAAGAAAAG. .......... ..........

LiSIDER2-32-253342d CAGTGCATGA TGA.ACGGGC TGAAAAGAGG .......... ..........

LiSIDER2-32-311078r .......... .......... .......... .......... ..........

LiSIDER2-32-1195026d CGGCGTGGAC ACGGTGA... .......... .......... ..........

LiSIDER2-32-1219109r CGGCGTGGAC ACGGTGA... .......... .......... ..........

LiSIDER2-32-1172151d .......... .......... .......... .......... ..........

LiSIDER2-32-542863d .......... .......... .......... .......... ..........

LiSIDER2-32-546470r CAGCAGAGAA CG........ .......... .......... ..........

LiSIDER2-32-520410d CAGCAGAGAA CG........ .......... .......... ..........

LiSIDER2-32-588727r GTTTAACT.. .......... .......... .......... ..........

LiSIDER2-32-642986d .......... .......... .......... .......... ..........

LiSIDER2-32-598168r GACCGAGAAA G......... .......... .......... ..........

LiSIDER2-32-919839r G......... .......... .......... .......... ..........

LiSIDER2-32-953057r G......... .......... .......... .......... ..........

LiSIDER2-32-937896d G......... .......... .......... .......... ..........

LiSIDER2-32-726312r .......... .......... .......... .......... ..........

LiSIDER2-32-752616r .......... .......... .......... .......... ..........

LiSIDER2-32-734536r .......... .......... .......... .......... ..........

LiSIDER2-32-755765r .......... .......... .......... .......... ..........

LiSIDER2-32-1284330d .......... .......... .......... .......... ..........

LiSIDER2-32-775723r .......... .......... .......... .......... ..........

LiSIDER2-32-794401r AGTGGGCACG TTGAACATCA TCGAAGAAAA AGCATCCACT TGCCTCTCCC

LiSIDER2-32-802492r AG........ .......... .......... .......... ..........

LiSIDER2-32-827594d AGTGGGCACG TTGAACATCA TCGAAGAAAA AGCATCCACT TGCCTCTCCC

LiSIDER2-32-808837r AG........ .......... .......... .......... ..........

LiSIDER2-32-769406r .......... .......... .......... .......... ..........

LiSIDER2-32-881720r GCG....... .......... .......... .......... ..........

LiSIDER2-32-890515r GCGCG..... .......... .......... .......... ..........

LiSIDER2-32-1496187d .......... .......... .......... .......... ..........

LiSIDER2-32-1532945d .......... .......... .......... .......... ..........

LiSIDER2-32-1545347d .......... .......... .......... .......... ..........

LiSIDER2-32-772687r CGGCGAGTGG .......... .......... .......... ..........

LiSIDER2-32-1141454r .......... .......... .......... .......... ..........

LiSIDER2-32-354985d .......

LiSIDER2-32-367858d .......

LiSIDER2-32-661708r .......

LiSIDER2-32-1405232d .......

LiSIDER2-32-169517r .......

LiSIDER2-32-185886r .......

LiSIDER2-32-420911d .......

LiSIDER2-32-460150r .......

LiSIDER2-32-456060d .......

LiSIDER2-32-991979r CCCAAAC

LiSIDER2-32-1017837r CCCAAAC

LiSIDER2-32-18439r .......

LiSIDER2-32-30662r .......

LiSIDER2-32-39696d .......

LiSIDER2-32-72349d .......

LiSIDER2-32-46425r .......

LiSIDER2-32-84516r .......

LiSIDER2-32-121058r .......

LiSIDER2-32-127617r .......

LiSIDER2-32-1128071r .......

LiSIDER2-32-222503d .......

LiSIDER2-32-232971d .......

LiSIDER2-32-253342d .......

LiSIDER2-32-311078r .......

LiSIDER2-32-1195026d .......

LiSIDER2-32-1219109r .......

LiSIDER2-32-1172151d .......

LiSIDER2-32-542863d .......

LiSIDER2-32-546470r .......

LiSIDER2-32-520410d .......

LiSIDER2-32-588727r .......

LiSIDER2-32-642986d .......

LiSIDER2-32-598168r .......

LiSIDER2-32-919839r .......

LiSIDER2-32-953057r .......

LiSIDER2-32-937896d .......

LiSIDER2-32-726312r .......

LiSIDER2-32-752616r .......

LiSIDER2-32-734536r .......

LiSIDER2-32-755765r .......

LiSIDER2-32-1284330d .......

LiSIDER2-32-775723r .......

LiSIDER2-32-794401r CACCC..

LiSIDER2-32-802492r .......

LiSIDER2-32-827594d CACCC..

LiSIDER2-32-808837r .......

LiSIDER2-32-769406r .......

LiSIDER2-32-881720r .......

LiSIDER2-32-890515r .......

LiSIDER2-32-1496187d .......

LiSIDER2-32-1532945d .......

LiSIDER2-32-1545347d .......

LiSIDER2-32-772687r .......

LiSIDER2-32-1141454r .......
