# Supplementary material for: The SIDER2 elements, interspersed repeated sequences that populate the Leishmania genomes, constitute subfamilies showing chromosomal proximity relationship
Source: BMC Genomics. 2008 Jun 2;9:263. doi: 10.1186/1471-2164-9-263 (PMC2424063; doi:10.1186/1471-2164-9-263)
Supplement: Additional file 2 — ClustalW2 alignment of the 27 LiSIDER2 sequences present in the L. infantum chromosome 20. [file 1471-2164-9-263-S2.doc]

**Alignment**

CLUSTAL 2.0.5 multiple sequence alignment

LiSIDER2-20-315r ------------------------------------------------------------

LiSIDER2-20-9815r ------------------------------------------------------------

LiSIDER2-20-26129r ------------------------------------------------------------

LiSIDER2-20-609548d GCACCGCCGCTGCACCGCAACGCTGGCCGGTCTGCTGCCGCTGCGACGTTTGTGCGGCCG

LiSIDER2-20-616384d GCACCGCCGCTGCACCGCAACGCTGGCCGGTCTGCTGCCGCTGCGACGTTTGTGCGGCCG

LiSIDER2-20-38171r ------------------------------------------------------------

LiSIDER2-20-517565r ------------------------------------------------------------

LiSIDER2-20-575257d ------------------------------------------------------------

LiSIDER2-20-510191r ------------------------------------------------------------

LiSIDER2-20-483061r ------------------------------------------------------------

LiSIDER2-20-112062r ------------------------------------------------------------

LiSIDER2-20-121152d ------------------------------------------------------------

LiSIDER2-20-104446d ------------------------------------------------------------

LiSIDER2-20-133376d ------------------------------------------------------------

LiSIDER2-20-172973d ------------------------------------------------------------

LiSIDER2-20-248078r ------------------------------------------------------------

LiSIDER2-20-543749d ------------------------------------------------------------

LiSIDER2-20-550589d ------------------------------------------------------------

LiSIDER2-20-520085d ------------------------------------------------------------

LiSIDER2-20-512317d ------------------------------------------------------------

LiSIDER2-20-334521d ------------------------------------------------------------

LiSIDER2-20-418908d ------------------------------------------------------------

LiSIDER2-20-441039d ------------------------------------------------------------

LiSIDER2-20-449285d ------------------------------------------------------------

LiSIDER2-20-432767r ------------------------------------------------------------

LiSIDER2-20-716639r ------------------------------------------------------------

LiSIDER2-20-208609r ------------------------------------------------------------

LiSIDER2-20-315r ------------------------------------------------------------

LiSIDER2-20-9815r ------------------------------------------------------------

LiSIDER2-20-26129r ------------------------------------------------------------

LiSIDER2-20-609548d CCGCGCGCCTCGCCGGCTGCTGTGCCCGCGCCCGCGAGCTGCGGCACTGCGGGGCCGCTG

LiSIDER2-20-616384d CCGCGCGCCTCGCTGGCTGCTGTGCCCGCGCCCGCGAGCTGCGGCACTGCGGGGCCGCTG

LiSIDER2-20-38171r ------------------------------------------------------------

LiSIDER2-20-517565r ------------------------------------------------------------

LiSIDER2-20-575257d ------------------------------------------------------------

LiSIDER2-20-510191r ------------------------------------------------------------

LiSIDER2-20-483061r ------------------------------------------------------------

LiSIDER2-20-112062r ------------------------------------------------------------

LiSIDER2-20-121152d ------------------------------------------------------------

LiSIDER2-20-104446d ------------------------------------------------------------

LiSIDER2-20-133376d ------------------------------------------------------------

LiSIDER2-20-172973d ------------------------------------------------------------

LiSIDER2-20-248078r ------------------------------------------------------------

LiSIDER2-20-543749d ------------------------------------------------------------

LiSIDER2-20-550589d ------------------------------------------------------------

LiSIDER2-20-520085d ------------------------------------------------------------

LiSIDER2-20-512317d ------------------------------------------------------------

LiSIDER2-20-334521d ------------------------------------------------------------

LiSIDER2-20-418908d ------------------------------------------------------------

LiSIDER2-20-441039d ------------------------------------------------------------

LiSIDER2-20-449285d ------------------------------------------------------------

LiSIDER2-20-432767r ------------------------------------------------------------

LiSIDER2-20-716639r ------------------------------------------------------------

LiSIDER2-20-208609r ------------------------------------------------------------

LiSIDER2-20-315r ------------------------------------------------------------

LiSIDER2-20-9815r ------------------------------------------------------------

LiSIDER2-20-26129r ------------------------------------------------------------

LiSIDER2-20-609548d GAACAGGCCTCTCCTGCTGCTGCTGCCGTGCCGTGCGCGCGTGGTGCTGGACTGCCGACG

LiSIDER2-20-616384d GAACAGGCCTCTCCTGCTGCTGCTGCCGTGCCGTGCGCGCGTGGTGCTGGACTGCCGACG

LiSIDER2-20-38171r ------------------------------------------------------------

LiSIDER2-20-517565r ------------------------------------------------------------

LiSIDER2-20-575257d ------------------------------------------------------------

LiSIDER2-20-510191r ------------------------------------------------------------

LiSIDER2-20-483061r ------------------------------------------------------------

LiSIDER2-20-112062r ------------------------------------------------------------

LiSIDER2-20-121152d ------------------------------------------------------------

LiSIDER2-20-104446d ------------------------------------------------------------

LiSIDER2-20-133376d ------------------------------------------------------------

LiSIDER2-20-172973d ------------------------------------------------------------

LiSIDER2-20-248078r ------------------------------------------------------------

LiSIDER2-20-543749d ------------------------------------------------------------

LiSIDER2-20-550589d ------------------------------------------------------------

LiSIDER2-20-520085d ------------------------------------------------------------

LiSIDER2-20-512317d ------------------------------------------------------------

LiSIDER2-20-334521d ------------------------------------------------------------

LiSIDER2-20-418908d ------------------------------------------------------------

LiSIDER2-20-441039d ------------------------------------------------------------

LiSIDER2-20-449285d ------------------------------------------------------------

LiSIDER2-20-432767r ------------------------------------------------------------

LiSIDER2-20-716639r ------------------------------------------------------------

LiSIDER2-20-208609r ------------------------------------------------------------

LiSIDER2-20-315r ------------------------------------------------------------

LiSIDER2-20-9815r ------------------------------------------------------------

LiSIDER2-20-26129r ------------------------------------------------------------

LiSIDER2-20-609548d GTGAGCGCGGTGCCGGTGCTGTCGCTGGACTGCGAGGCGGAGCGCGGCTGCGCTGGCGTG

LiSIDER2-20-616384d GTGAGCGCGGTGCCGGTGCTGTCGCTGGACTGCGAGGCGGAGCGCGGCTGCGCTGGCGTG

LiSIDER2-20-38171r ------------------------------------------------------------

LiSIDER2-20-517565r ------------------------------------------------------------

LiSIDER2-20-575257d ------------------------------------------------------------

LiSIDER2-20-510191r ------------------------------------------------------------

LiSIDER2-20-483061r ------------------------------------------------------------

LiSIDER2-20-112062r ------------------------------------------------------------

LiSIDER2-20-121152d ------------------------------------------------------------

LiSIDER2-20-104446d ------------------------------------------------------------

LiSIDER2-20-133376d ------------------------------------------------------------

LiSIDER2-20-172973d ------------------------------------------------------------

LiSIDER2-20-248078r ------------------------------------------------------------

LiSIDER2-20-543749d ------------------------------------------------------------

LiSIDER2-20-550589d ------------------------------------------------------------

LiSIDER2-20-520085d ------------------------------------------------------------

LiSIDER2-20-512317d ------------------------------------------------------------

LiSIDER2-20-334521d ------------------------------------------------------------

LiSIDER2-20-418908d ------------------------------------------------------------

LiSIDER2-20-441039d ------------------------------------------------------------

LiSIDER2-20-449285d ------------------------------------------------------------

LiSIDER2-20-432767r ------------------------------------------------------------

LiSIDER2-20-716639r ------------------------------------------------------------

LiSIDER2-20-208609r ------------------------------------------------------------

LiSIDER2-20-315r ------------------------------------------------------------

LiSIDER2-20-9815r ------------------------------------------------------------

LiSIDER2-20-26129r ------------------------------------------------------------

LiSIDER2-20-609548d GCGCCTGCGACGGTGGCCGCCACGGTGGCGCCCGCAGTGCACGTGGCGGCGCCGGTGTGC

LiSIDER2-20-616384d GCGCCTGCGACGGTGGCCGCCACGGTGGCGCCCGCAGTGCACGTGGCGGCGCCGGTGTGC

LiSIDER2-20-38171r ------------------------------------------------------------

LiSIDER2-20-517565r ------------------------------------------------------------

LiSIDER2-20-575257d ------------------------------------------------------------

LiSIDER2-20-510191r ------------------------------------------------------------

LiSIDER2-20-483061r ------------------------------------------------------------

LiSIDER2-20-112062r ------------------------------------------------------------

LiSIDER2-20-121152d ------------------------------------------------------------

LiSIDER2-20-104446d ------------------------------------------------------------

LiSIDER2-20-133376d ------------------------------------------------------------

LiSIDER2-20-172973d ------------------------------------------------------------

LiSIDER2-20-248078r ------------------------------------------------------------

LiSIDER2-20-543749d ------------------------------------------------------------

LiSIDER2-20-550589d ------------------------------------------------------------

LiSIDER2-20-520085d ------------------------------------------------------------

LiSIDER2-20-512317d ------------------------------------------------------------

LiSIDER2-20-334521d ------------------------------------------------------------

LiSIDER2-20-418908d ------------------------------------------------------------

LiSIDER2-20-441039d ------------------------------------------------------------

LiSIDER2-20-449285d ------------------------------------------------------------

LiSIDER2-20-432767r ------------------------------------------------------------

LiSIDER2-20-716639r ------------------------------------------------------------

LiSIDER2-20-208609r ------------------------------------------------------------

LiSIDER2-20-315r ----------------------------CCCGGATGACGGTGGGGACAGCCAGTGCGTGG

LiSIDER2-20-9815r ----------------------------CCCGGATGACAGTGGGGACAGCCAGTGCGTGG

LiSIDER2-20-26129r --------------------------------------------------CGGTGCGTGG

LiSIDER2-20-609548d GGGACGCGCAGGCTTTCGGCGATGCCTGCTGCCGCGCTGGTGGAGGCGTTCAGGGCGACG

LiSIDER2-20-616384d GGGACGCGCAGGCTTTCGGCGATGCCTGCTGCCGCGCTGGTGGAGGCGTTCAGGGCGACG

LiSIDER2-20-38171r ------------------------------------------------------------

LiSIDER2-20-517565r ------------------------------------------------------------

LiSIDER2-20-575257d ------------------------------------------------------------

LiSIDER2-20-510191r ------------------------------------------------------------

LiSIDER2-20-483061r ------------------------------------------------------------

LiSIDER2-20-112062r ------------------------------------------------------------

LiSIDER2-20-121152d ------------------------------------------------------------

LiSIDER2-20-104446d ------------------------------------------------------------

LiSIDER2-20-133376d ---------------------------------CCTTTTGTTGTCATGGACATCGTACAG

LiSIDER2-20-172973d ---------------------------------CCTTTTGTTGTCATGGACATCGTACAG

LiSIDER2-20-248078r ------------------------------------------------------------

LiSIDER2-20-543749d ------------------------------------------------------------

LiSIDER2-20-550589d ------------------------------------------------------------

LiSIDER2-20-520085d ------------------------------------------------------------

LiSIDER2-20-512317d ------------------------------------------------------------

LiSIDER2-20-334521d ------------------------------------------------------------

LiSIDER2-20-418908d ------------------------------------------------------------

LiSIDER2-20-441039d -----------------------------------------CGCGCACACCTCAGCGCGC

LiSIDER2-20-449285d -----------------------------GGAAGGGGGGGCCGCGCACACCTCAGCGCGC

LiSIDER2-20-432767r -----------------------------GAAAGGGGGGGGCGCGCACACCTCAGCGCGC

LiSIDER2-20-716639r -----------------------------------------CTCTGATGACACAGAACAC

LiSIDER2-20-208609r ------------------------------------------------------------

LiSIDER2-20-315r CATCACAGG---GCTGCGTGTATTTTCAGTCTGTGGGGAAGCAAAGCAGCCCTGCTATCC

LiSIDER2-20-9815r CATCACAGG---GCTGCGTGTATTTTCAGTCTGTGGGGAAGCAAAGCAGCCCTGCTATCC

LiSIDER2-20-26129r CATCACAGG---GCTGCGTGTATTTTCAGTCTGTGGGGAAGCAAAGCAGCCCTGCTATCC

LiSIDER2-20-609548d TGCGAGGGGCACGTGGCGGACGTTGCGTGCACGCCGCAGGGCAAGGCGCTGCTGCAGGCG

LiSIDER2-20-616384d TGCGAGGGGCACGTGGCGGACGTTGCGTGCACGCCGCAGGGCAAGGCGCTGCTGCAGGCG

LiSIDER2-20-38171r ------------------------------------------------------------

LiSIDER2-20-517565r ------------------------------------------------------------

LiSIDER2-20-575257d ----------------------------------------TGACGTGTGGACCCCGCTCA

LiSIDER2-20-510191r ------------------------------------------------------------

LiSIDER2-20-483061r ----------------------------------------TGACGTGTGGACACCGCTCA

LiSIDER2-20-112062r ---------------------------------------------------CACCGCTCA

LiSIDER2-20-121152d ------------------------------------------------------------

LiSIDER2-20-104446d ----------------------------------------------GGCGACACCGCTCA

LiSIDER2-20-133376d CAACTCTTTCGGTGCGTGTTCGGCGCTCTGAGTCCGTCATGGCGGGGGCGACACCGCTCA

LiSIDER2-20-172973d CAACTCTTTCGGTGCGTGTTCGGCGCTCTGAGTCCGTCATGGCGGGGGCGACACCGCTCA

LiSIDER2-20-248078r ----------------------------------------------------AGCACACC

LiSIDER2-20-543749d ----------------------------------CCCTGGTGACGTGTGGACCCCGCTCA

LiSIDER2-20-550589d ----------------------------------CCCTCGTGACGTGTGGACCCCGCTCA

LiSIDER2-20-520085d -----------------------------------------GACGTGTGGACCCCGCTCA

LiSIDER2-20-512317d ----------------------------------CCCTGGTGACGTGTGGACCCCGCTCA

LiSIDER2-20-334521d ------------------------------------------------------------

LiSIDER2-20-418908d ---------------------------------------------------CAACGCTGT

LiSIDER2-20-441039d AGCATTCTCGGTGTCCAGTGCCCCACTCATACTGTCTGCAGAGAAGCCAGGCAAGCCTCC

LiSIDER2-20-449285d AGCATTCTCGGTGTCCAGTGCCCCACTCATACTGTCTGCAGAGAAGCCAGGCAAGCCTCC

LiSIDER2-20-432767r AGCATTCTCGGTGTCCAGTGCCCCACTCATACTGTCTGCAGAGAAGCCAGGCAAGCCTCC

LiSIDER2-20-716639r ATCAGCGTCGCATCTCAGGGTTCAGTACCCACTCTGAGTGTGGGAGGAAGCCAAGCAGCC

LiSIDER2-20-208609r ------------------------------------------------------------

LiSIDER2-20-315r CCCCC--TACCGATGCGGA-------GCCACCTCTGATGGTGACAGGGCCAAGCACCTAC

LiSIDER2-20-9815r CCCCCCCTACCGATGCGGA-------GCCACCTCTGATGGTGACAGGGCCAAGCACCTAC

LiSIDER2-20-26129r CCCCC--TACCGATGCGGA-------GCCACCTCTGATGGTGACAGGGCCAAGCACCTAC

LiSIDER2-20-609548d GCGCTGCGGACGCAGCGGCCGGAGGTGCTGGACTCTGTGGTGACGGAGCT--GTGCCCAC

LiSIDER2-20-616384d GCGCTGCGGACGCAGCGGCCGGAGGTGCTGGACTCTGTGGTGACGGAGCT--GTGCCCAC

LiSIDER2-20-38171r ------------------------------------------------------------

LiSIDER2-20-517565r ------------------------------------------------------------

LiSIDER2-20-575257d GTGCGCCGTATCTCAGGGTCCACTACCCACG--CACTGTGTGGTGAAGCCAAGCAGCCCT

LiSIDER2-20-510191r ------------------------------------------------------------

LiSIDER2-20-483061r GTGCGTCGTATCTCAGGGTCCACTACCCACG--CACTGTGTGGTGAAGCCAAGCAGCCCT

LiSIDER2-20-112062r GTGCTTCGTACCTCAGGGTCCAGCACACCCGCTCTCTGTGTCGGGAAGCCAAGCAGCCCC

LiSIDER2-20-121152d ------------------------------------------------------------

LiSIDER2-20-104446d GTGCTTCGTACCTCAGGGTCCAGCACACCCGCTCTCTGTGTCGGGAAGCCAAGCAGCCCC

LiSIDER2-20-133376d GTGCTTCGTACCTCAGGGTCCAGCACACCCGCTCTCTGTGTCGGGAAGCCAAGCAGCCCC

LiSIDER2-20-172973d GTGCTTCGTACCTCAGGGTCCAGCACACCCGCTCTCTGTGTCGGGAAGCCAAGCAGCCCC

LiSIDER2-20-248078r CACTCTCTGTGGGAGAAGCCAAGCAGTCACCTTCCCCACTTCACACACACACACACACCT

LiSIDER2-20-543749d GTGCGCCGTATCTCAAAGTCCACTACCCACG--CACTGTGTGGTGAAGCCAAGCAGCCCT

LiSIDER2-20-550589d GTGCGCCGTATCTCAAAGTCCACTACCCACG--CACTGTGTGGTGAAGCCAAGCAGCCCT

LiSIDER2-20-520085d GTGCGCCGTATCTCAGGGTCCACTACCCACG--CACTGTGTGGTGAAGCCAAGCAGCCCT

LiSIDER2-20-512317d GTGCGCCGTATCTCAGGGTCCACTACCCACG--CACTGTGTGGTGAAGCCAAGCAGCCCT

LiSIDER2-20-334521d -------------------------------------GCATCGCGGGGTCAGATACCCGC

LiSIDER2-20-418908d CTTACACCCTGATGA--CGTGGGAACAGCTCCGCCCCGCATCG-GGAGTCAGAGACGCAC

LiSIDER2-20-441039d CT-ATCCCCTGCCCA--TGCCAAAACACTTCCGCTG-GCGACA--GGGCCAGGCACCCGC

LiSIDER2-20-449285d CTTATCCCCTGCCCA--TGCGAAAACACTTCCGCTT-GCGACA--GGGCCAGGCACCCGC

LiSIDER2-20-432767r CT-ATCCCCTGCCCA--TGCCAAAACACTTCCGCTG-GCGACA--GGGCCAGGCACCCGC

LiSIDER2-20-716639r CCTATACTCGGCTAAAATGCCGAGCCACTTCTGCTG-GTGACA--GGTTCAAGCGCCTAC

LiSIDER2-20-208609r ------------------------------------------------------------

LiSIDER2-20-315r GGC----GTAGGGGGAGGCCGGAGCTATGCACCGCTGCTGATGCCGGC--GGTGATGTGG

LiSIDER2-20-9815r GGC----GTAGGGGGAGGCCGGAGCTATGCACCGCTGCTGATGCCGGC--GGTGATGTGG

LiSIDER2-20-26129r GGC----GTAGGGGGAGGCCGGAGCTATGCACCGCTGCTGATGCCGGC--GGTGATGTGG

LiSIDER2-20-609548d GGCT---GCGCGACGTGGCGGTGGACGTGCACGGGTGCCACGTGCTGC--GGACGC-TGG

LiSIDER2-20-616384d GGCT---GCGCGACGTGGCGGTGGACGTGCACGGGTGCCACGTGCTGC--GGACGC-TGG

LiSIDER2-20-38171r ----------------------------------------------------------GG

LiSIDER2-20-517565r -------------------------------------------------CCGTCAGGTCC

LiSIDER2-20-575257d CCCCAGCCCTATCCCCTGCC-GGATGCAGAACCACCTCTGGTGGTGACACGGCCAGGCCC

LiSIDER2-20-510191r ------------------------------------------------------------

LiSIDER2-20-483061r CCCCAGCCCTATCCCCTGCC-GGATGCAGAACCACCTCTAGTGGTGACACGGCCAGGCCC

LiSIDER2-20-112062r TT-------ATGTCCCTGCC--GATGTCAGACCACCTCTGGCGCAGACAGGGTCAAGCAC

LiSIDER2-20-121152d ------------------------------------------------------------

LiSIDER2-20-104446d TT-------ATGTCCCTGCC--GATGTCAGACCACCTCTGGCGCAGACAGGGTCAAGCAC

LiSIDER2-20-133376d TT-------ATGTCCCTGCC--GATGTCAGACCACCTCTGGCGCAGACAGGGTCAAGCAC

LiSIDER2-20-172973d TT-------ATGTCCCTGCC--GATGTCAGACCACCTCTGGCGCAGACAGGGTCAAGCAC

LiSIDER2-20-248078r CA-------TATCCCCTGCCCAAATGCCAAACCACTTCTGGTGGCGGCAGGGCTAGGC-C

LiSIDER2-20-543749d CCCCAGCCCTATCCCCTGCC-GGATGCAGAACCACCTCTGGTGGTGACATGGCCAGGCCC

LiSIDER2-20-550589d CCCCAGCCCTATCCCCTGCC-GGATGCAGAACCACCTCTGGTGGTGACATGGCCAGGCCC

LiSIDER2-20-520085d CCCCAGCCCTATCCCCTGCC-GGATGCAGAACCACCTCTGGTGGTGACACGGCCAGGCCC

LiSIDER2-20-512317d CCCCAGCCCTATCCCCTGCC-GGATGCAGAACCACCTCTGGTGGTGACATGGCCAGGCCC

LiSIDER2-20-334521d GA----TATGGGGGGAGGTCAGAGCGATGTATCGTAGCCGATGTCGGC--TACCAGGTCC

LiSIDER2-20-418908d ------TCTGTGAAGGAGTCGGAGCAGTGTATCGCTATTGGTGTCGGC--GCTCAGGTGC

LiSIDER2-20-441039d G------ACGTGGGGAGGTCAAGGCGATGTATCGCTGCTGACGTCGGC--GCTCAGGTCC

LiSIDER2-20-449285d G------ACGTGGGGAGGTCAAGGCGATGTATCGCTGCTGACGTCGGC--GCTCAGGTCC

LiSIDER2-20-432767r G------ACGTGGGGAGGTCAAGGCGATGTATCGCTGCTGACGTCGGC--GCTCAGGTCC

LiSIDER2-20-716639r G------ACGTGGGGAAGTGAGAGCAATTCATCGCTGCTGATGCTGGA--GACCATGTCC

LiSIDER2-20-208609r --------------------------------------CGTTTTCGAGGTGGTCATGACG

LiSIDER2-20-315r AGGATGGCGT-TGCTCCGGAGCGACCTGCGA--------CCGTGGACACGTGTGTG-CCG

LiSIDER2-20-9815r AGGATGGCGT-TGCTCCGGAGCGACCTGCGA--------CCGTGGACACGTGTGTG-CCG

LiSIDER2-20-26129r AGGATGGCGT-TGCTCCGGAGCGACCTGCGA--------CCGTGGACACGTGTGTG-CCG

LiSIDER2-20-609548d TGGA-GGCGTGCACTGCGGAGCAGACGGAGG--------C-GCTGATTGGCGCGAT-GCA

LiSIDER2-20-616384d TGGA-GGCGTGCACTGCGGAGCAGACGGAGG--------C-GCTGATTGGCGCGAT-GCA

LiSIDER2-20-38171r TAGATGGCGT-TGCTCCGGAGCGACCTGCGA--------CCGTGGACACGTGTGTG-CCG

LiSIDER2-20-517565r TGGATGGCGT-TGCGTTGGAGCGACCCGCGACAGTGGGTCAGCGTACACGTCTGCG-CCA

LiSIDER2-20-575257d TGGATGGCGT-TGCGTTGGAGCGACCTGCGACAGTGGGTCAGCGTACACGTCTGCG-CCA

LiSIDER2-20-510191r ---------------------------------------------ACACGTCTGCG-CCA

LiSIDER2-20-483061r TGGATGGCGT-TGCGTTAGAGCGACCTGCGACAGTGGGTCAGCGTACACGTCTGCG-CCA

LiSIDER2-20-112062r CCCACGGCGC-AAGGAGGGGCCAGA--GCGA-GCCGCGACAGTGCAGACGTGTGCG-CCA

LiSIDER2-20-121152d ---------------------CAGA--GCGA-GCCGCGACAGTGCAGACGTGTGCG-CCA

LiSIDER2-20-104446d CCCACGGCGC-AAGGAGGGGCCAGA--GCGA-GCCGCGACAGTGCAGACGTGTGCG-CCA

LiSIDER2-20-133376d CCCACGGCGC-AAGGAGGGGCCAGA--GCGA-GCCGCGACAGTGCAGACGTGTGCG-CCA

LiSIDER2-20-172973d CCCACGGCGC-AAGGAGGGGCCAGA--GCGA-GCCGCGACAGTGCAGACGTGTGCG-CCA

LiSIDER2-20-248078r TCCATGGGGT-GCGGAAGG-TCAGA--GCGA---TGTATCGCTGCTGATGTCGGCGGCCA

LiSIDER2-20-543749d TGGATGGCGT-TGCGTTGGAGCGACCTGCGACAGTGGGTCAGCGTACACGTCTGCG-CCA

LiSIDER2-20-550589d TGGATGGCGT-TGCGTTGGAGCGACCTGCGACAGTGGGTCAGCGTACACGTCTGCG-CCA

LiSIDER2-20-520085d TGGATGGCGT-TGCGTTGGAGCGACCTGCGACAGTGGGTCAGCGTACACGTCTGTG-CCA

LiSIDER2-20-512317d TGGATGGCGT-TGCGTTGGAGCGACCTGCGACAGTGGGTCAGCGTACACGTCTGCG-CCA

LiSIDER2-20-334521d TGGACGGCGT-TTCGTCGGTGCGAATAGCAA--------GAATGAACGCGCTTTTA-CAG

LiSIDER2-20-418908d TGGACGGTGT-GATGTCGGAGCGGCCTGGGT--------CAGCGAACACGTCAGCA-CCA

LiSIDER2-20-441039d TGGACGGTGT-TGCGCCGTAGCGACCTGCGA--------CAGTGCACGTGCTTGCA-GTA

LiSIDER2-20-449285d TGGACGGTGT-TGCGCCGTAGCGACCTGCGA--------CAGTGCACGTGCTTGCA-GTA

LiSIDER2-20-432767r TGGACGGTGT-TGCGCCGTAGCGACCTGCGA--------CAGTGCACGTGCTTGCA-GTA

LiSIDER2-20-716639r TGGATGGCGC-TGCGTTGGGGGGGGCTGCCA--------CAGTGAGCACGCGTGTA-CCA

LiSIDER2-20-208609r GCGCGAGGGCATCGCCCAGTGCATCCAATCA-CGCGGCCCGGCTCACCCACTCTCT-GCA

LiSIDER2-20-315r CCGATACGATGGGCGAAGTGTCAGCGCGACTGCAGCGCATCTCACCCGGCCCTGACTGTG

LiSIDER2-20-9815r CCGATACGATGGGCGAAGTGTCAGCGCGACTGCAGCGCATCTCACCCGGCCCTGACTGTG

LiSIDER2-20-26129r CCGATACGATGGGCGAAGTGTCAGCGCGACTGCAGCGCATCTCACCCGGCCCTGACTGTG

LiSIDER2-20-609548d CGCGTCGGTTGTGCTGAACATGTGC---ACTGCGTCGCAGTACACGCGGCGCACGCTGCA

LiSIDER2-20-616384d CGCGTCGGTTGTGCTGAACATGTGC---ACTGCGTCGCAGTACACGCGGCGCACGCTGCA

LiSIDER2-20-38171r CCGATACGATGGGCGAAGTGTCAGCGCGACTGCAGCGCATCTCACCCGGCCCTGACTGTG

LiSIDER2-20-517565r CCCACGTGACAGGCAGAGCGTCAGCGTGACGCGAGCGCG---TATCCCACCCCCGGCCCT

LiSIDER2-20-575257d TCCACGTGACAGGCAGAGCGTCAGCGTGACGCGAGCGCA---TATCCCACCCCCGGCCCT

LiSIDER2-20-510191r TCCATGTGACAGGCAGAGCTTCAGCGTGACGCGAGCGCA---TATCCCACCCCCGGCCCT

LiSIDER2-20-483061r TCCACGTGACAGGCAGAGCGTCAGCGTGACGCGAGCGCA---TATCCCACCCCCGGCCCT

LiSIDER2-20-112062r TCCATGGGATGGGCAATGCGCCAGCCTGACCCGCACC-----TACCTCACCA--GGCCCT

LiSIDER2-20-121152d TCCATGGGATGGGCAATGCGCCAGCCTGACCCGCACC-----TACCTCACCA--GGCCCT

LiSIDER2-20-104446d TCCATGGGATGGGCAATGCGCCAGCCTGACCCGCACC-----TACCTCACCA--GGCCCT

LiSIDER2-20-133376d TCCATGGGATGGGCAATGCGCCAGCCTGACCCGCACC-----TACCTCACCA--GGCCCT

LiSIDER2-20-172973d TCCATGGGATGGGCAATGCGCCAGCCTGACCCGCACC-----TACCTCACCA--GGCCCT

LiSIDER2-20-248078r GGTCCTGGATGG-CACTGCGGCGGCGTGACTTGAGCG-----TATCCCACAC--GGTCCT

LiSIDER2-20-543749d TCCACGTGACAGGCAGAGCGTCAGCGTGACGCGAGCGCA---TATCCCACCCCCGGCCCT

LiSIDER2-20-550589d TCCACGTGACAGGCAGAGCGTCAGCGTGACGCGAGCGCA---TATCCCACCCCCGGCCCT

LiSIDER2-20-520085d TCCACGTGACAGGCAGAGCGTCAGCGTGACGCGAGCGCA---TATCCCACCCCCGGCCCT

LiSIDER2-20-512317d TCCACGTGACAGGCAGAGCGTCAGCGTGACGCGAGCGCA---TATCCCACCCCCGGCCCT

LiSIDER2-20-334521d TTCACATCATAGGCGGAGTGCCACCGTGGCCCAAACGAA---TCCAACTCCCGGGCACCC

LiSIDER2-20-418908d CCCATATGACAGGCAGAGCGTCAGCGTGATGCAAGCGCA---TATCCCACCCCCGCCCCT

LiSIDER2-20-441039d CGCATATGGTAGGCAGAGTGTCAGCGTGACGCGAGCGCA---TAT--CCCACGCACCCCT

LiSIDER2-20-449285d CGCATATGGTAGGCAGAGTGTCAGCGTGACGCGAGCGCA---TAT--CCCACGCACCCCT

LiSIDER2-20-432767r CGCATATGGTAGGCAGAGTGTCAGCGTGACGCGAGCGCA---TAT--CCCACGCACCCCT

LiSIDER2-20-716639r TCCATGACT---ACTCAGTGTCAGCATGGCT--AGCGTG---TAT--CTCGCTTCGCCCT

LiSIDER2-20-208609r TGTGAGCGG-AGGCCAAGCAGCCCCCCACCTCTGCCGCATGCCAGACCACTTCTGGTGCT

* * * *

LiSIDER2-20-315r CTGGTGGTGAGGGGAGCCTGAGC---GCCCGCCCACGGCTGCACCGCGTGCTGGTTGGGA

LiSIDER2-20-9815r CTGGTGGTGAGGGGAGCCTGAGC---GCCCGCCCACGGCTGCACCGCGTGCTGGTTGGGA

LiSIDER2-20-26129r CTGGTGGTGAGGGGAGCCTGAGC---GCCCGCCCACGGCTGCACCGCGTGCTGGTTGGGA

LiSIDER2-20-609548d GTCGCTGTTCGAGCGGCGCGAGGTGGACCTGTCTGCGCTTGTGC-ACGTGCTCGC--GGA

LiSIDER2-20-616384d GTCGCTGTTCGAGCGGCGCGAGGTGGACCTGTCTGCGCTTGTGC-ACGTGCTCGC--GGA

LiSIDER2-20-38171r CTGGTGGTGAGGGGAGCCTGAGC---GCCCGCCCACGGCTGCACCGCGTGCTGGTTGGGA

LiSIDER2-20-517565r CGCGCTGCCT--ACAGCGGTGTGA-GGGAGGCCTGAGGCACCC---CGAGGGGAGGATGC

LiSIDER2-20-575257d CGCGCTGCCT--ACAGCGGTGTGG-GGGAGGCCTGAGGCACCC---CGAGGGGAGGATGC

LiSIDER2-20-510191r CGCGCTGCCT--ACAGCGGTGTGG-GGGAGGCCTGAGGCACCC---CGAGGGGAGGATGC

LiSIDER2-20-483061r CGCGCTGCCT--ACAGCGGTGTGG-GGGAGGCCTGAGGCACCC---CGAGGGGAGGATGC

LiSIDER2-20-112062r CGCACTGCCCTGCCGGTGGGGTGGTGGGGAGCCTGGGCCACCC---CGATGGCG--GCAC

LiSIDER2-20-121152d CGCACTGCCCTGCCGGTGGGGTGGTGGGGAGCCTGGGCCACCC---CGATGGCG--GCAC

LiSIDER2-20-104446d CGCACTGCCCTGCCGGTGGGGTGGTGGGGAGCCTGGGCCACCC---CGATGGCG--GCAC

LiSIDER2-20-133376d CGCACTGCCCTGCCGGTGGGGTGGTGGGGAGCCTGGGCCACCC---CGATGGCG--GCAC

LiSIDER2-20-172973d CGCACTGCCCTGCCGGTGGGGTGGTGGGGAGCCTGGGCCACCC---CGATGGCG--GCAC

LiSIDER2-20-248078r C--ACTGTT----CAGTGGTGTGG---AGAGTCTGCCCCATCA---GAGGGGGGATGTAC

LiSIDER2-20-543749d CGCGCTGCCT--ACAGCGGTGTGG-GGGAGGCCTGAGGCACCC---CGAGGGGAGGATGC

LiSIDER2-20-550589d CGCGCTGCCT--ACAGCGGTGTGG-GGGAGGCCTGAGGCACCC---CGAGGGGAGGATGC

LiSIDER2-20-520085d CGCGCTGCCT--ACAGCGGTGTGG-GGGAGGCCTGAGGCACCC---CGAGGGGAGGATGC

LiSIDER2-20-512317d CGCGCTGCCT--ACAGCGGTGTGG-GGGAGGCCTGAGGCACCC---CGAGGGGAGGATGC

LiSIDER2-20-334521d ATTTCTACCT--TC---------G--TGAAGCCCAAATGCCCCCCTGAAGGGGGATGCGG

LiSIDER2-20-418908d CGCGCTGCCT--ACAGCGGTGTGG--GGAGGCCTGAGGTATCCCTTGGGGGCGGGGGGGG

LiSIDER2-20-441039d GGCCCTCGCT--GC---------------TGTCTGCAGAGG----TGTGGGGGGCCTGAG

LiSIDER2-20-449285d GGCCCTCGCT--GC---------------TGTCTGCAGAGG----TGTGGGGGGCCTGAG

LiSIDER2-20-432767r GGCCCTCGCT--GC---------------TGTCTGCAGAGG----TGTGGGGGGCCTGAG

LiSIDER2-20-716639r CACACTCCCT--AC-----TAGTG--TGAAGCCTGAGGCCACC--CCGAGAAGGATGAAG

LiSIDER2-20-208609r GACAGGGCCAAGCACCCACTACGCAAGGAAGGTGAGAGCGATGCATCGCTGATGATGT--

*

LiSIDER2-20-315r CAATGCGAGCGTCTGCGAGGCGAC---CTGC-GTGGCGGATGTGGGTG--GGTCGAGCTT

LiSIDER2-20-9815r CAATGCGAGCGTCTGCGAGGCGAC---CTGC-GTGGCGGATGTGGGTG--GGTCGAGCTT

LiSIDER2-20-26129r CAATGCGAGCGTCTGCGAGGCGAC---CTGC-GTGGCGGATGTGGGTG--GGTCGAGCTT

LiSIDER2-20-609548d CAACGCGGGGTACCTTGCGGCGAC--GCAGCAGGGGTGCATCTCGCTGATGCGCGTGTTC

LiSIDER2-20-616384d CAACGCGGGGTACCTTGCGGCGAC--GCAGCAGGGGTGCATCTCGCTGATGCGCGTGTTC

LiSIDER2-20-38171r CAATGCGAGCGTCTGCGAGGCGAC---CTGC-GTGGCGGATGTGGGTG--GGTCGAGCTT

LiSIDER2-20-517565r AC-----------CAGGCGGCGACCGGCATGATGCGGGAGCGGCGGTGAGGCGACCTGCG

LiSIDER2-20-575257d AC-----------CAGGCGGCGACCGGCATGATGCGGGAGCGGCGGTGAGGCGACCTGCG

LiSIDER2-20-510191r AC-----------CAGGCGGCAACCGGCATGATGCGGGAGCGGCGGTGAGGCGACCTGCG

LiSIDER2-20-483061r AC-----------CAGGCGGCGACCGGCATGATGCAGGAGCGGCGGTGAGGCGACCTGCG

LiSIDER2-20-112062r C-------------GCGTGGCGACCGGCACAATGGG--AGCGGCTGTGCGGCGACCTGCG

LiSIDER2-20-121152d C-------------GCGTGGCGACCGGCACAATGGG--AGCGGCTGTGCGGCGACCTGCG

LiSIDER2-20-104446d C-------------GCGTGGCGACCGGCACAATGGG--AGCGGCTGTGCGGCGACCTGCG

LiSIDER2-20-133376d C-------------GCGTGGCGACCGGCACAATGGG--AGCGGCTGTGCGGCGACCTGCG

LiSIDER2-20-172973d C-------------GCGTGGCGACCGGCACAATGGG--AGCGGCTGTGCGGCGACCTGCG

LiSIDER2-20-248078r CA-----------GGGGTGGCTCCAGGCATCATGGGGAAGCAGCTGTGAGGCTGCCTGCG

LiSIDER2-20-543749d AC-----------CAGGCGGCGACCGGCATGATGCGGGAGCGGCGGTGAGGC-GTGTCTA

LiSIDER2-20-550589d AC-----------CAGGCGGCGACCGGCATGATGCGGGAGCGGCGGTGAGGC-GTGTCTA

LiSIDER2-20-520085d AC-----------CAGGCGGCGACCGGCATGATGCGGGAGCGGCGGTGAGGC-GTGTCTA

LiSIDER2-20-512317d AC-----------CAGGCGGCGACCGGCATGATGCGGGAGCGGCGGTGAGGC-GTGTCTA

LiSIDER2-20-334521d -------------CGGGCGACAGCCGGGATCATAGGAG--CGACTGCGGGGCGGCTGGCG

LiSIDER2-20-418908d GGGGGGCGCACATCAGGTGGCAGCCGGCATGATGGGAGAGCGACTGTGAGGCGGCCGGCA

LiSIDER2-20-441039d -------------CGCCCCTCCCCCCCCCCCGAGGGGAATGCACCAGGTGGCGACCAGCA

LiSIDER2-20-449285d -------------CGCCCCTCCCCCCCC--CGAGGGGAATGCACCAGGTGGCGACCAGCA

LiSIDER2-20-432767r -------------CGCCCCTCCCCCCC----GAGGGGAATGCACCAGGTGGCGACCAGCA

LiSIDER2-20-716639r -------------CAGGTGGTGCCCGGCATAATGAGAG--GGGCTGTGGGCCGACCTGCA

LiSIDER2-20-208609r --------------CGGCGTTGAATCGCAGGATGGCGTCGCGTCAGAAAGGCCTGCGAGG

LiSIDER2-20-315r GAGGCGGATGCCGTG--CTCAGATGACTGAGTGGGCGCGT--------TGCCG----TGG

LiSIDER2-20-9815r GAGGCGGATGCCGTG--CTCAGATGACTGAGTGGGCGCGT--------TGCCG----TGG

LiSIDER2-20-26129r GAGGCGGATGCCGTG--CTCAGATGACTGAGTGGGCGCGT--------TGCCG----TGG

LiSIDER2-20-609548d GAGCTGTGCGACGCG-GCGCAGAAGGCGGAGCTGGTGCGCG-AGCTGCTGCCGAAGTTGG

LiSIDER2-20-616384d GAGCTGTGCGACGCG-GCGCAGAAGGCGGAGCTGGTGCGCG-AGCTGCTGCCGAAGTTGG

LiSIDER2-20-38171r GAGGCGGATGCCGTG--CTCAGATGACTGAGTGGGCGCG------------------TTG

LiSIDER2-20-517565r GAGCG-AGAGGTGTGTGGGCAGAGTTCGAGGCGGGGGGCCGTGCCCTCCGACGACTGAGT

LiSIDER2-20-575257d GAGCG-AGAGGTGTGTGGGCAGAGTTCGAGGCAGGGGGCCGTGCTCTTCGACGACTGAGT

LiSIDER2-20-510191r GAGCG-AGAGGTGTGTGGGTAGAGTCCGAGGCAGGGGGCCGTGCTCTCCGACGACTGAGT

LiSIDER2-20-483061r GAGCG-AGAGGTGTGTGGGTAGAGTCCGAGGCAGGGGACCGTGCTCTCCGACGACTGAGT

LiSIDER2-20-112062r AGGC--GGGGATGGT-GGGCGGAGTCTGAGTCCGAGGCG---ATGCTCCGATGAATGAGT

LiSIDER2-20-121152d AGGC--GGGGATGGT-GGGCGGAGTCTGAGTCCGAGGCG---ATGCTCCGATGAATGAGT

LiSIDER2-20-104446d AGGC--GGGGATGGT-GGGCGGAGTCTGAGTCCGAGGCG---ATGCTCCGATGAATGAGT

LiSIDER2-20-133376d AGGC--GGGGATGGT-GGGCGGAGTCTGAGTCCGAGGCG---ATGCTCCGATGAATGAGT

LiSIDER2-20-172973d AGGC--GGGGATGGT-GGGCGGAGTCTGAGTCCGAGGCG---ATGCTCCGATGAATGAGT

LiSIDER2-20-248078r AATA--AGGTGTGTG-GGGTGGGAGTGGGGGGTAGAGTG---GGCTCGTGGTTGGCGACA

LiSIDER2-20-543749d GGGC--TGCTTCTCA-CCACGCGATGTGAGGCCTGTGAC---AGGCCTGGCCGAGTGGTG

LiSIDER2-20-550589d GGGC--TGCTTCTCA-CCACGCGATGTGAGGCCTGTGAC---AGGCCTGGCCGAGTGGTG

LiSIDER2-20-520085d GGGC--TGCTTCTCA-CCACGCGATGTGAGGCCTGTGAC---AGGCCTGGCCGAGTGGTG

LiSIDER2-20-512317d GGGC--TGCTTCTCA-CCACGCGATGTGAGGCCTGTGAC---AGGCCTGGCCGAGTGGTG

LiSIDER2-20-334521d ACGCAGGGGGGGGGGCGAGTGGGGTTTGAGGCGGGGGCC-GCGCCCTCAGATGACCGGGT

LiSIDER2-20-418908d AAGC------GAAGGCGAGTGGAGTTTGAGTCAGAGGCC-GTGC--TGAGCCGACCAAGT

LiSIDER2-20-441039d TCGT----GTGCGCGCGGGGAGAGTTTTGTGCAGAGGCC-GTGCTCTCAGATGGCTGGGT

LiSIDER2-20-449285d TCGT----GTGCGCGCGGGGAGAGTTTTGTGCAGAGGCC-GTGCTCTCAGATGGCTGGGT

LiSIDER2-20-432767r TCGT----GTGCGCGCGGGGAGAGTTTTGTGCAGAGGCC-GTGCTCTCAGATGGCTGGGT

LiSIDER2-20-716639r GAGC-----AGCTTGTCGGTAGAGCTTGAGGTAGGGGTC-GTGC--TCCAATGGCGGAGT

LiSIDER2-20-208609r CAGGGGGTAGGTGGGCCAGTCGAGTTGGAGTCAGGGGCC-GTCCTCTCGCATGACTGAGC

*

LiSIDER2-20-315r C-GTG-TGTGTGTGTGTG------------------------------------------

LiSIDER2-20-9815r C-GTG-TGTGTGTGTGTGTGTGTGCGG-CTGCTTGG--CACCACGCGATGGGGCCCTGTG

LiSIDER2-20-26129r C-GTG-TGTGTGTGTGTGT----GCGG-CTGCTTGG--CACCACGCGATGGGGCCCTGTG

LiSIDER2-20-609548d CTGCGCTGTCGATGGATGCGTTTGCGAACTACATGGTGCAGTGCGCGATCGAGCACAGCG

LiSIDER2-20-616384d CTGCGCTGTCGATGGATGCGTTTGCGAACTACATGGTGCAGTGCGCGATCGAGCACAGCG

LiSIDER2-20-38171r CCGTGGCGTGTGTGTGTGTGTGTGCGG-CTGCTTGG--CACCACGCGATGGGGCCCTGTG

LiSIDER2-20-517565r CGGCGCAGCACCCTAGCGCGTGCCCACCCCTG-CTTCGCGCCACGCG-------------

LiSIDER2-20-575257d CGGTGCAGCACCCTAGCGCGTGCCCACCCCTG-CTTCGCGCCACGCGCGATGTGG-----

LiSIDER2-20-510191r CGGCGCAGCACCCTAGCGCG----------------------------------------

LiSIDER2-20-483061r CGGCGCAGCACTCTAGCGCGTGCCCACCCCTG-CTTCGCGCCACGCGATGTGG-------

LiSIDER2-20-112062r AGGCGCATCGCTGTAACGTGTGTGTGTGTGCG-CGTGTTCGGCTGCTTCGCACGACGCGG

LiSIDER2-20-121152d AGGCGCATCGCTGTAACGTGTGTGTGTGTGCG-CGTGTTCGGCTGCTTCGCACGACGCGG

LiSIDER2-20-104446d AGGCGCATCGCTGTAACGTGTGTGTGTGTGCG-CGTGTTCGGCTGCTTCGCACGACGCGG

LiSIDER2-20-133376d AGGCGCATCGCTGTAACGTGTGTGTGTGTGCG-CGTGTTCGGCTGCTTCGCACGACGCGG

LiSIDER2-20-172973d AGGCGCATCGCTGTAACGTGTGTGTGTGTGCG-CGTGTTCGGCTGCTTCGCACGACGCGG

LiSIDER2-20-248078r TG----------------------------------------------------------

LiSIDER2-20-543749d TTCAGCGCATGCTCTGTGGCAGAATGGACACC-TTGAATGAA------------------

LiSIDER2-20-550589d TTCAGCGCATGCTCTGTGGCAGAATGGACACC-TTGAATGAA------------------

LiSIDER2-20-520085d TTCAGCGCATGCTCTGCGCCAGAATGGGCAAG-TTGAA----------------------

LiSIDER2-20-512317d TTCAGCGCATGCTCTGCGCCAGAATGGGCAAG-TTAAA----------------------

LiSIDER2-20-334521d CGGCGGATTGCTGTGGTGGATGCGTCCGCGGC-TACCACGCGGCTGG-G-----------

LiSIDER2-20-418908d CGGCGCACTGCACTAGCGTGTGTGTCCACGGC-TGCTTCGCACCACG-TC-ACGTTGCCC

LiSIDER2-20-441039d CGGCGCACTGCTACAACGCCTGTGTCTACGGCATGCTTCGCACCACGCGG-ATGGGGCCT

LiSIDER2-20-449285d CGGCGCACTGCTACAACGCCTGTGTCTACGGCATGCTTCGCACCACGCGG-ATGGGGCCT

LiSIDER2-20-432767r CGGCGCACTGCTACAACGCCTGTGTCTACGGCATGCTTCGCACCACGCGG-ATGGGGCCT

LiSIDER2-20-716639r CGGCACATTGCTCTGAGG--CGTGCCTACGGC-TGCTTCGCACCACGCGATGTGGAGACT

LiSIDER2-20-208609r CGGCGCATTGCTGTAACGCGCGTGCCTACGACATGCTTCGCACCGCGCGGTTGGGCACCT

LiSIDER2-20-315r ------------------------------------------------------------

LiSIDER2-20-9815r A--GAATGGCGGGCGGTGCAGCGGACCTGAGCTCATTC----------------------

LiSIDER2-20-26129r A--GAATGGCGGGCGGTGCAGCAGAGCTTGACTGATTT----------------------

LiSIDER2-20-609548d ATCGCACGACGGCCG-CGCAGTACGTTGTGGCCCACTTCACCGGCAACATATTGCAGATG

LiSIDER2-20-616384d ATCGCACGACGGCCG-CGCAGTACGTTGTGGCCCACTTCACCGGCAACATATTGCAGATG

LiSIDER2-20-38171r A--GAATGGCGGGCGGTGCAGCGGACCTGAGCTGATTC----------------------

LiSIDER2-20-517565r ------------------------------------------------------------

LiSIDER2-20-575257d ------------------------------------------------------------

LiSIDER2-20-510191r ------------------------------------------------------------

LiSIDER2-20-483061r ------------------------------------------------------------

LiSIDER2-20-112062r ATGGGGGCCTGTGACAGGGCTGGTGTGTGTGTGTGTGTG----GTGTGCGTGTGGTGTGC

LiSIDER2-20-121152d ATGGGGGCCTGTGACAGGGCTGGTGTGTGTGTGTGTG-----------------GTGTGC

LiSIDER2-20-104446d ATGGGGGCCTGTGACAGGGCTGGTGTGTGTGTGTGTGTG----GTGTGCGTGTGGTGTGC

LiSIDER2-20-133376d ATGGGGGCCTGTGACAGGGCTGGTGTGTGTGTGTGTGTGTGTGGTGTGTGTGTGGTGTGC

LiSIDER2-20-172973d ATGGGGGCCTGTGACAGGGCTGGTGTGTGTGTGTGTGTGTG--GTGTGTGTGTGGTGTGC

LiSIDER2-20-248078r ------------------------------------------------------------

LiSIDER2-20-543749d ------------------------------------------------------------

LiSIDER2-20-550589d ------------------------------------------------------------

LiSIDER2-20-520085d ------------------------------------------------------------

LiSIDER2-20-512317d ------------------------------------------------------------

LiSIDER2-20-334521d ------------------------------------------------------------

LiSIDER2-20-418908d GTGGCAGGCCGAGGGGC-------------------------------------------

LiSIDER2-20-441039d GTGGCAGGGGCCGAGGGACGTATGGCCTCGTGCTGTATGGCAGAGCAACGCACTCTTGTT

LiSIDER2-20-449285d GTGGCAGGGGCCGAGGGACGTATGGCCTCGTGCTGTATGGCAGAGCAACGCACTCTTGTT

LiSIDER2-20-432767r GTGGAAGGGGCCGAGGGACGTATGGCCTCGTGCTGTATGGCAGAGCAACGCACTCTTGTT

LiSIDER2-20-716639r GTGGCGGGGCGAGGGATCGAGTGGAATTTCGCTCATGTTCTCTGGCAGAGAATGGACACG

LiSIDER2-20-208609r GTGACACGTCCGAGGTAGAATG--------------------------------------

LiSIDER2-20-315r ------------------------------------------------------------

LiSIDER2-20-9815r ------------------------------------------------------------

LiSIDER2-20-26129r ------------------------------------------------------------

LiSIDER2-20-609548d AGCTGCAACAAGCACTCCAGCAACGTGCTGGAGGTTGTCCTGCGGTGCTGTGGCGAGGTC

LiSIDER2-20-616384d AGCTGCAACAAGCACTCCAGCAACGTGCTGGAGGTTGTCCTGCGGTGCTGTGGCGAGGTC

LiSIDER2-20-38171r ------------------------------------------------------------

LiSIDER2-20-517565r ------------------------------------------------------------

LiSIDER2-20-575257d ------------------------------------------------------------

LiSIDER2-20-510191r ------------------------------------------------------------

LiSIDER2-20-483061r ------------------------------------------------------------

LiSIDER2-20-112062r GTGTGGTGGTGGGGAGGTGCAGCTTAGTTTAACCTCATGCTCTATGGCCGGAGTACTGCA

LiSIDER2-20-121152d GTGTGGTGGTGGGGAGGTGCAGCTTAGTTTAACCTCATGCTCTATGGCCGGAGTACTGAA

LiSIDER2-20-104446d GTGTGGTGGTGGGGAGGTGCAGCTTAGTTTGACCTCATGCTCTATGGCCGGAGTACTGCA

LiSIDER2-20-133376d GTGTGGTGGTGGGGAGGTGCAGCTTAGTTTGACCTCATGCTCTATGGCCGGAGTACTGCA

LiSIDER2-20-172973d GTGTGGTGGTGGGGAGGTGCAGCTTAGTTTGACCTCATGCTCTATGGCCGGAGTACTGCA

LiSIDER2-20-248078r ------------------------------------------------------------

LiSIDER2-20-543749d ------------------------------------------------------------

LiSIDER2-20-550589d ------------------------------------------------------------

LiSIDER2-20-520085d ------------------------------------------------------------

LiSIDER2-20-512317d ------------------------------------------------------------

LiSIDER2-20-334521d ------------------------------------------------------------

LiSIDER2-20-418908d ------------------------------------------------------------

LiSIDER2-20-441039d GTCAGGAAAGGAGGACGAAGAAGAGCGACCTCACCAGCGTCG------------------

LiSIDER2-20-449285d GGCAGGAAAGGAGGACGAAGAAGAGCGACCTCACCAGCGTCG------------------

LiSIDER2-20-432767r GCC---------------------------------------------------------

LiSIDER2-20-716639r TTGAA-------------------------------------------------------

LiSIDER2-20-208609r ------------------------------------------------------------

LiSIDER2-20-315r ------------------------------------------------------------

LiSIDER2-20-9815r ------------------------------------------------------------

LiSIDER2-20-26129r ------------------------------------------------------------

LiSIDER2-20-609548d CCGGCGGTGCGGCGCCTTTTTCTGGACGAGCTGGTTTTCAACCCAGCCGCCCTGAAGGAG

LiSIDER2-20-616384d CCGGCGGTGCGGCGCCTTTTTCTGGACGAGCTGGTTTTCAACCCAGCCGCCCTGAAGGAG

LiSIDER2-20-38171r ------------------------------------------------------------

LiSIDER2-20-517565r ------------------------------------------------------------

LiSIDER2-20-575257d ------------------------------------------------------------

LiSIDER2-20-510191r ------------------------------------------------------------

LiSIDER2-20-483061r ------------------------------------------------------------

LiSIDER2-20-112062r CATGGCGAAAGAAG----------------------------------------------

LiSIDER2-20-121152d CTTGAAGAACTCCTTCCCTTC---------------------------------------

LiSIDER2-20-104446d CACGACGAAAGAGA----------------------------------------------

LiSIDER2-20-133376d CATGGCGAAAGAAGAACTGTTC--------------------------------------

LiSIDER2-20-172973d CACGACGAAGGAAG----------------------------------------------

LiSIDER2-20-248078r ------------------------------------------------------------

LiSIDER2-20-543749d ------------------------------------------------------------

LiSIDER2-20-550589d ------------------------------------------------------------

LiSIDER2-20-520085d ------------------------------------------------------------

LiSIDER2-20-512317d ------------------------------------------------------------

LiSIDER2-20-334521d ------------------------------------------------------------

LiSIDER2-20-418908d ------------------------------------------------------------

LiSIDER2-20-441039d ------------------------------------------------------------

LiSIDER2-20-449285d ------------------------------------------------------------

LiSIDER2-20-432767r ------------------------------------------------------------

LiSIDER2-20-716639r ------------------------------------------------------------

LiSIDER2-20-208609r ------------------------------------------------------------

LiSIDER2-20-315r ------------------------------------------------------------

LiSIDER2-20-9815r ------------------------------------------------------------

LiSIDER2-20-26129r ------------------------------------------------------------

LiSIDER2-20-609548d GTGGTGGGCGATCTGTACGGGAACTTTGTGGTGCAGGCGCTGATCGGCGTTGTGACGAAC

LiSIDER2-20-616384d GTGGTGGGCGATCTGTACGGGAACTTTGTGGTGCAGGCGCTGATCGGCGTTGTGACGAAC

LiSIDER2-20-38171r ------------------------------------------------------------

LiSIDER2-20-517565r ------------------------------------------------------------

LiSIDER2-20-575257d ------------------------------------------------------------

LiSIDER2-20-510191r ------------------------------------------------------------

LiSIDER2-20-483061r ------------------------------------------------------------

LiSIDER2-20-112062r ------------------------------------------------------------

LiSIDER2-20-121152d ------------------------------------------------------------

LiSIDER2-20-104446d ------------------------------------------------------------

LiSIDER2-20-133376d ------------------------------------------------------------

LiSIDER2-20-172973d ------------------------------------------------------------

LiSIDER2-20-248078r ------------------------------------------------------------

LiSIDER2-20-543749d ------------------------------------------------------------

LiSIDER2-20-550589d ------------------------------------------------------------

LiSIDER2-20-520085d ------------------------------------------------------------

LiSIDER2-20-512317d ------------------------------------------------------------

LiSIDER2-20-334521d ------------------------------------------------------------

LiSIDER2-20-418908d ------------------------------------------------------------

LiSIDER2-20-441039d ------------------------------------------------------------

LiSIDER2-20-449285d ------------------------------------------------------------

LiSIDER2-20-432767r ------------------------------------------------------------

LiSIDER2-20-716639r ------------------------------------------------------------

LiSIDER2-20-208609r ------------------------------------------------------------

LiSIDER2-20-315r ------------------------------------------------------------

LiSIDER2-20-9815r ------------------------------------------------------------

LiSIDER2-20-26129r ------------------------------------------------------------

LiSIDER2-20-609548d CCGATGGAGTTCAAGCGCGTGGAGGACCGCCTGCGCCCCGCACTGGTGGGGTGCCAGTTC

LiSIDER2-20-616384d CCGATGGAGTTCAAGCGCGTGGAGGACCGCCTGCGCCCCGCACTGGTGGGGTGCCAGTTC

LiSIDER2-20-38171r ------------------------------------------------------------

LiSIDER2-20-517565r ------------------------------------------------------------

LiSIDER2-20-575257d ------------------------------------------------------------

LiSIDER2-20-510191r ------------------------------------------------------------

LiSIDER2-20-483061r ------------------------------------------------------------

LiSIDER2-20-112062r ------------------------------------------------------------

LiSIDER2-20-121152d ------------------------------------------------------------

LiSIDER2-20-104446d ------------------------------------------------------------

LiSIDER2-20-133376d ------------------------------------------------------------

LiSIDER2-20-172973d ------------------------------------------------------------

LiSIDER2-20-248078r ------------------------------------------------------------

LiSIDER2-20-543749d ------------------------------------------------------------

LiSIDER2-20-550589d ------------------------------------------------------------

LiSIDER2-20-520085d ------------------------------------------------------------

LiSIDER2-20-512317d ------------------------------------------------------------

LiSIDER2-20-334521d ------------------------------------------------------------

LiSIDER2-20-418908d ------------------------------------------------------------

LiSIDER2-20-441039d ------------------------------------------------------------

LiSIDER2-20-449285d ------------------------------------------------------------

LiSIDER2-20-432767r ------------------------------------------------------------

LiSIDER2-20-716639r ------------------------------------------------------------

LiSIDER2-20-208609r ------------------------------------------------------------

LiSIDER2-20-315r ------------------------------------------------------------

LiSIDER2-20-9815r ------------------------------------------------------------

LiSIDER2-20-26129r ------------------------------------------------------------

LiSIDER2-20-609548d GCGGCGAAGATCGAGGGGAAGATCAAGGCGAAGCGTCCAGTCCCGCCCCACGCGGGCGGT

LiSIDER2-20-616384d GCGGCGAAGATCGAGGGGAAGATCAAGGCGAAGCGTCCAGTCCCGCCCCACGCGGGCGGT

LiSIDER2-20-38171r ------------------------------------------------------------

LiSIDER2-20-517565r ------------------------------------------------------------

LiSIDER2-20-575257d ------------------------------------------------------------

LiSIDER2-20-510191r ------------------------------------------------------------

LiSIDER2-20-483061r ------------------------------------------------------------

LiSIDER2-20-112062r ------------------------------------------------------------

LiSIDER2-20-121152d ------------------------------------------------------------

LiSIDER2-20-104446d ------------------------------------------------------------

LiSIDER2-20-133376d ------------------------------------------------------------

LiSIDER2-20-172973d ------------------------------------------------------------

LiSIDER2-20-248078r ------------------------------------------------------------

LiSIDER2-20-543749d ------------------------------------------------------------

LiSIDER2-20-550589d ------------------------------------------------------------

LiSIDER2-20-520085d ------------------------------------------------------------

LiSIDER2-20-512317d ------------------------------------------------------------

LiSIDER2-20-334521d ------------------------------------------------------------

LiSIDER2-20-418908d ------------------------------------------------------------

LiSIDER2-20-441039d ------------------------------------------------------------

LiSIDER2-20-449285d ------------------------------------------------------------

LiSIDER2-20-432767r ------------------------------------------------------------

LiSIDER2-20-716639r ------------------------------------------------------------

LiSIDER2-20-208609r ------------------------------------------------------------

LiSIDER2-20-315r ----------------------------------------

LiSIDER2-20-9815r ----------------------------------------

LiSIDER2-20-26129r ----------------------------------------

LiSIDER2-20-609548d GCGGTGCACCATCATCCCTACCCGCAGTCGCACCAGCAGC

LiSIDER2-20-616384d GCGGTGCACCATCATCCCTACCCGCAGTCGCACCAGCAAC

LiSIDER2-20-38171r ----------------------------------------

LiSIDER2-20-517565r ----------------------------------------

LiSIDER2-20-575257d ----------------------------------------

LiSIDER2-20-510191r ----------------------------------------

LiSIDER2-20-483061r ----------------------------------------

LiSIDER2-20-112062r ----------------------------------------

LiSIDER2-20-121152d ----------------------------------------

LiSIDER2-20-104446d ----------------------------------------

LiSIDER2-20-133376d ----------------------------------------

LiSIDER2-20-172973d ----------------------------------------

LiSIDER2-20-248078r ----------------------------------------

LiSIDER2-20-543749d ----------------------------------------

LiSIDER2-20-550589d ----------------------------------------

LiSIDER2-20-520085d ----------------------------------------

LiSIDER2-20-512317d ----------------------------------------

LiSIDER2-20-334521d ----------------------------------------

LiSIDER2-20-418908d ----------------------------------------

LiSIDER2-20-441039d ----------------------------------------

LiSIDER2-20-449285d ----------------------------------------

LiSIDER2-20-432767r ----------------------------------------

LiSIDER2-20-716639r ----------------------------------------

LiSIDER2-20-208609r ----------------------------------------
